# Supplementary material for: 4-Substituted Thieno[3,2-d]pyrimidines as Dual-Stage Antiplasmodial Derivatives
Source: Pharmaceuticals (Basel). 2022 Jul 1;15(7):820. doi: 10.3390/ph15070820 (PMC9323797; doi:10.3390/ph15070820)
Supplement: Supplementary file 1 [file pharmaceuticals-15-00820-s001.zip › pharmaceuticals-1786122-supplementary.pdf]

SUPPORTING INFORMATION

4-Substituted Thieno[3,2-*d*]pyrimidines as Dual-Stage Antiplasmodial Derivatives

Prisca Lagardère,[a] Romain Mustière,[b] Nadia Amanzougaghene,[c] Sébastien Hutter,[d] Jean-François Franetich,[c] Nadine Azas,[d] Patrice Vanelle,[b,g] Pierre Verhaeghe,[e, f] Nicolas Primas [b, g], Dominique Mazier,[c] Nicolas Masurier[a*] and Vincent Lisowski[a*]

a. Institut des Biomolécules Max Mousseron, UMR 5247, CNRS, Université de Montpellier, ENSCM, UFR des Sciences Pharmaceutiques et Biologiques, Montpellier, France.
b. Aix Marseille Université, CNRS, ICR UMR 7273, Equipe Pharmaco-Chimie Radicalaire, Faculté de Pharmacie, Marseille, France.
c. Centre d'Immunologie et des Maladies Infectieuses (CIMI), INSERM, CNRS, Sorbonne Université, Paris, France.
d. Aix Marseille Université, IRD, AP-HM, SSA, VITROME, Marseille, France.
e. LCC-CNRS, Université de Toulouse, CNRS UPR 8241, UPS, Toulouse, France.
f. CHU de Nîmes, service de pharmacie, Nîmes, France.
g. Service Central de la Qualité et de l'Information Pharmaceutiques, AP-HM, Hôpital Conception, Marseille, France.

*Correspondence: nicolas.masurier@umontpellier.fr; Tel.: +33-4117-59642; vincent.lisowski@umontpellier.fr; Tel. +33-411 7-59599

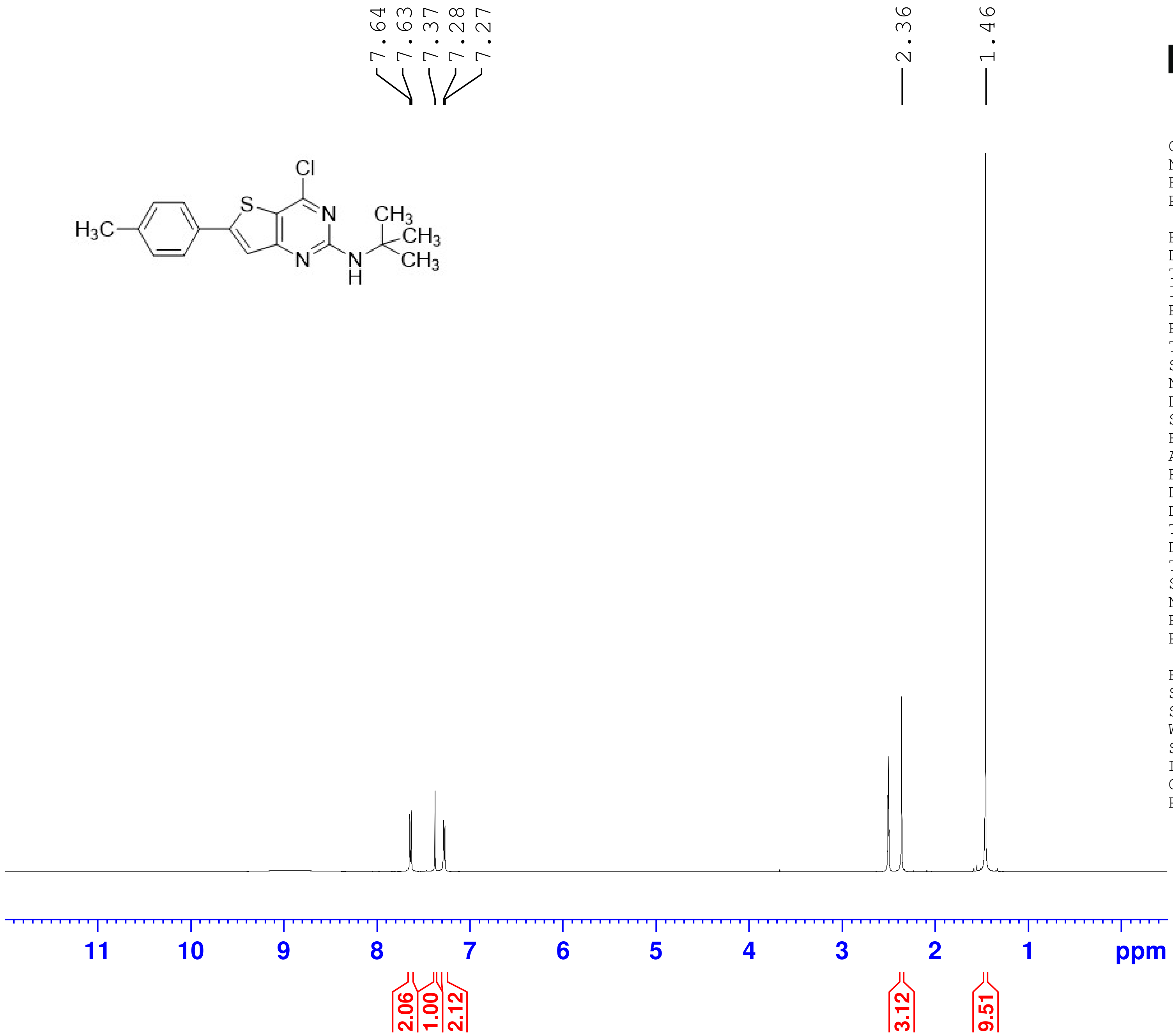

BRUKER

Current Data Parameters
NAME
EXPNO 20
PROCNO 1

F2 - Acquisition Parameters

Date_	20190227
Time	20.17 h
INSTRUM	Spect
PROBHD	Z136122_0002 (
PULPROG	zg30
TD	65536
SOLVENT	DMSO
NS	32
DS	2
SWH	8012.820 Hz
FIDRES	0.244532 Hz
AQ	4.0894465 sec
RG	10
DW	62.400 usec
DE	10.00 usec
TE	298.0 K
D1	1.50000000 sec
TD0	1
SFO1	500.1735012 MHz
NUC1	1H
P1	12.90 usec
PLW1	7.00000000 W

F2 - Processing parameters

SI	65536
SF	500.1700087 MHz
WDW	EM
SSB	0
LB	0.10 Hz
GB	0
PC	1.00

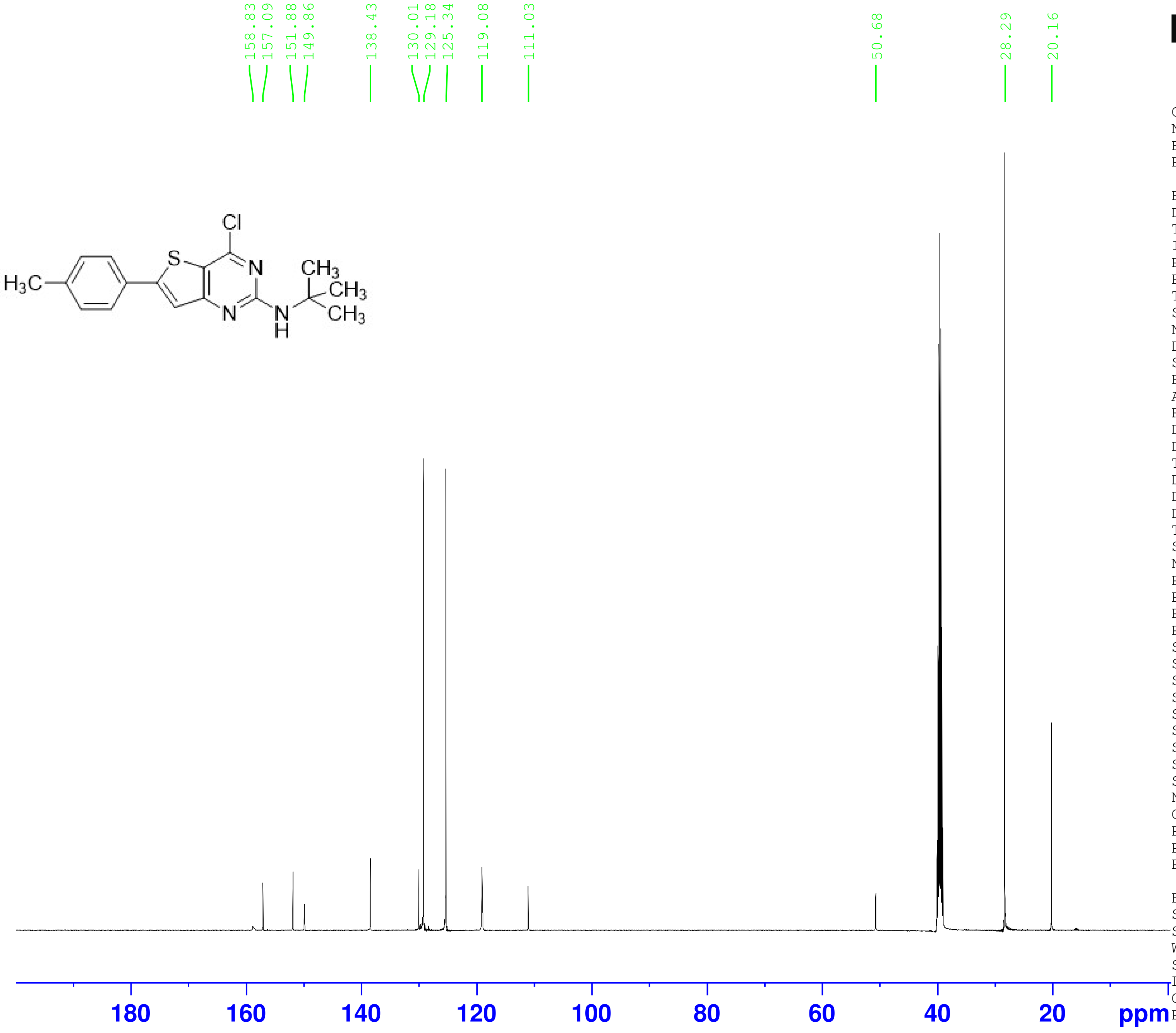

Current Data Parameters
NAME LP0014
EXPNO 21
PROCNO 1

F2 - Acquisition Parameters
Date_ 20190228
Time 0.40 h
INSTRUM Spect
PROBHD Z136122_0002 (
PULPROG udeft
TD 20586
SOLVENT DMSO
NS 4096
DS 0
SWH 28846.154 Hz
FIDRES 2.802502 Hz
AQ 0.3568240 sec
RG 575
DW 17.333 usec
DE 18.00 usec
TE 298.0 K
D1 3.00000000 sec
D12 0.00002000 sec
D20 200.00000000 sec
TD0 1
SFO1 125.7810526 MHz
NUC1 13C
P1 10.00 usec
P13 2000.00 usec
P26 500.00 usec
PLW1 26.00000000 W
SPNAM[5] Crp60comp.4
SPOAL5 0.500
SPOFFS5 0 Hz
SPW5 3.97250009 W
SPNAM[8] Crp60,0.5,20.1
SPOAL8 0.500
SPOFFS8 0 Hz
SPW8 3.97250009 W
SFO2 500.1720007 MHz
NUC2 1H
CPDPRG[2 waltz16
PCPD2 80.00 usec
PLW2 7.00000000 W
PLW12 0.18200999 W

F2 - Processing parameters
SI 32768
SF 125.7679719 MHz
WDW EM
SSB 0
LB 2.00 Hz
GB 0
PC 1.40

LP0233

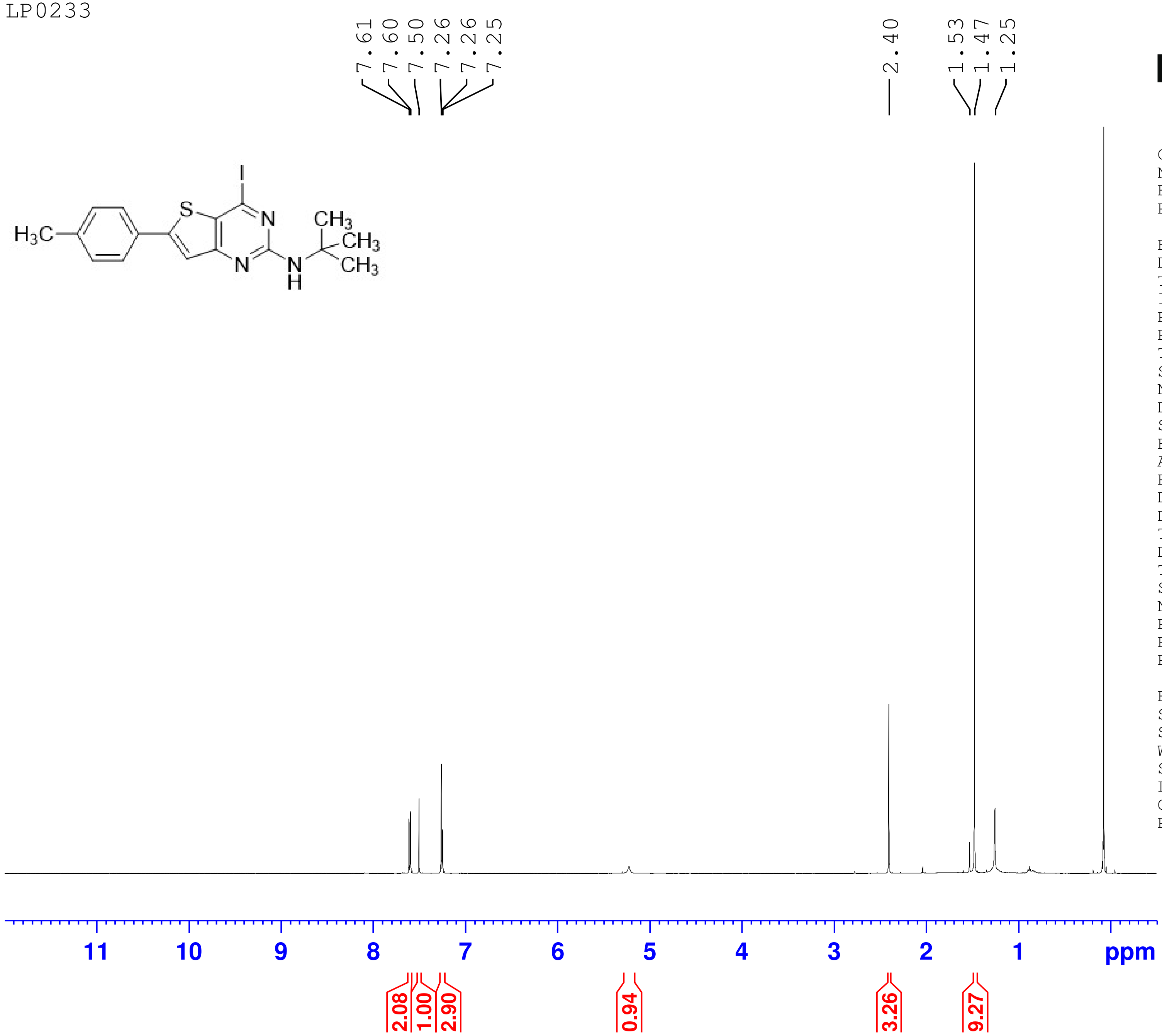

Current Data Parameters
NAME LAG-43-LP0233
EXPNO 11
PROCNO 1

F2 - Acquisition Parameters
Date_ 20210112
Time 20.13 h
INSTRUM Spect
PROBHD Z136122_0002 (
PULPROG zg30
TD 65536
SOLVENT CDCl3
NS 16
DS 2
SWH 10000.000 Hz
FIDRES 0.305176 Hz
AQ 3.2767999 sec
RG 10
DW 50.000 usec
DE 10.00 usec
TE 298.0 K
D1 1.50000000 sec
TD0 1
SFO1 500.1730885 MHz
NUC1 1H
P0 4.30 usec
P1 12.90 usec
PLW1 7.00000000 W

F2 - Processing parameters
SI 65536
SF 500.1700130 MHz
WDW EM
SSB 0
LB 0.10 Hz
GB 0
PC 2.00

LP0233 / CDCl3

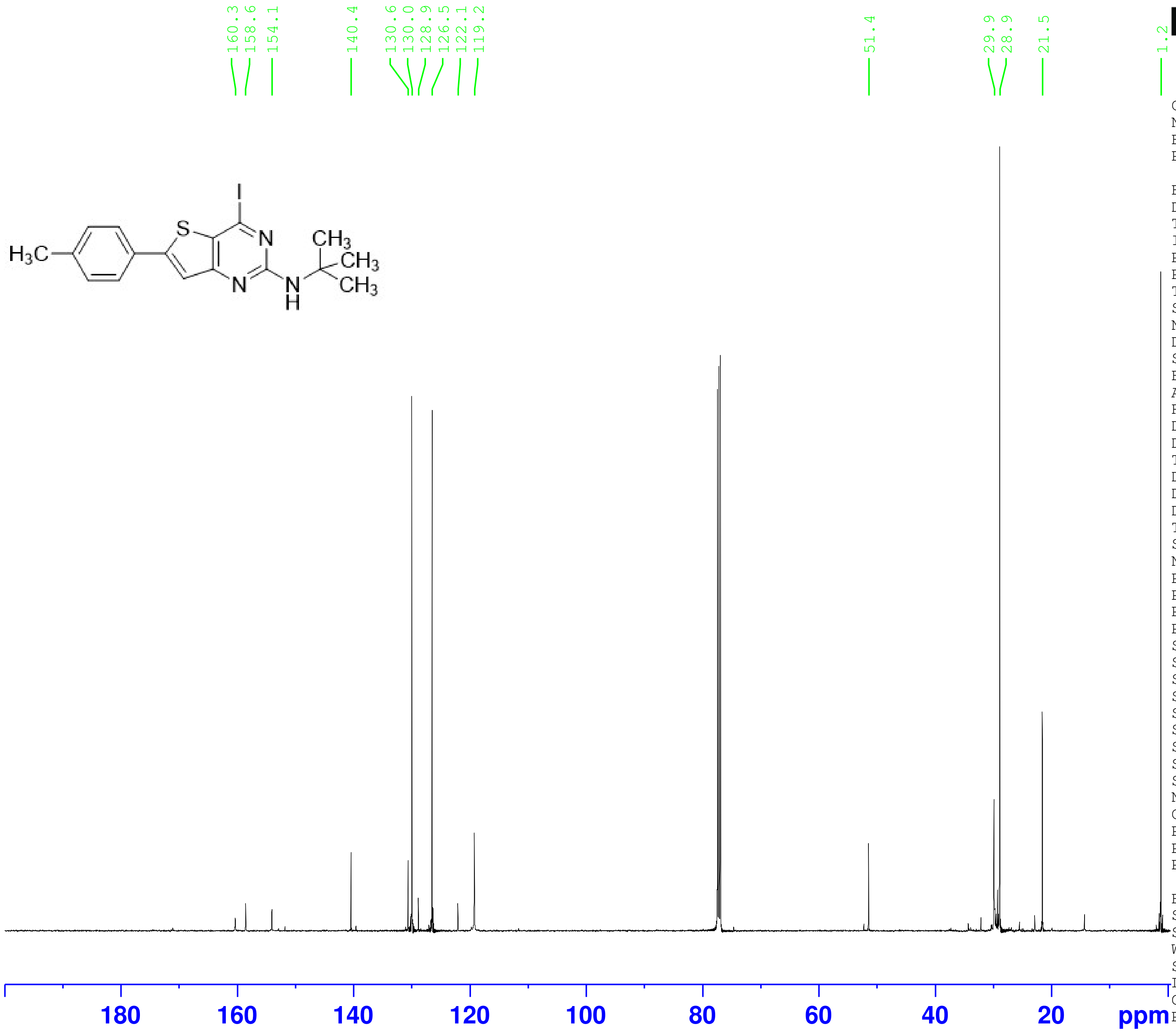

Current Data Parameters

NAME	LAG-43-LP0233
EXPNO	12
PROCNO	1

F2 - Acquisition Parameters

Date_	20210112
Time	22.29 h
INSTRUM	Spect
PROBHD	Z136122_0002 (
PULPROG	udeft
TD	16384
SOLVENT	CDCl3
NS	2048
DS	0
SWH	28846.154 Hz
FIDRES	3.521259 Hz
AQ	0.2839893 sec
RG	812
DW	17.333 usec
DE	18.00 usec
TE	298.0 K
D1	3.00000000 sec
D12	0.00002000 sec
D20	200.00000000 sec
TD0	1
SFO1	125.7810526 MHz
NUC1	13C
P1	10.00 usec
P13	2000.00 usec
P26	500.00 usec
PLW1	26.00000000 W
SPNAM[5]	Crp60comp.4
SPOAL5	0.500
SPOFFS5	0 Hz
SPW5	3.97250009 W
SPNAM[8]	Crp60,0.5,20.1
SPOAL8	0.500
SPOFFS8	0 Hz
SPW8	3.97250009 W
SFO2	500.1720007 MHz
NUC2	1H
CPDPRG[2	waltz16
PCPD2	80.00 usec
PLW2	7.00000000 W
PLW12	0.18200999 W

F2 - Processing parameters

SI	32768
SF	125.7678297 MHz
WDW	EM
SSB	0
LB	2.00 Hz
GB	0
PC	2.00

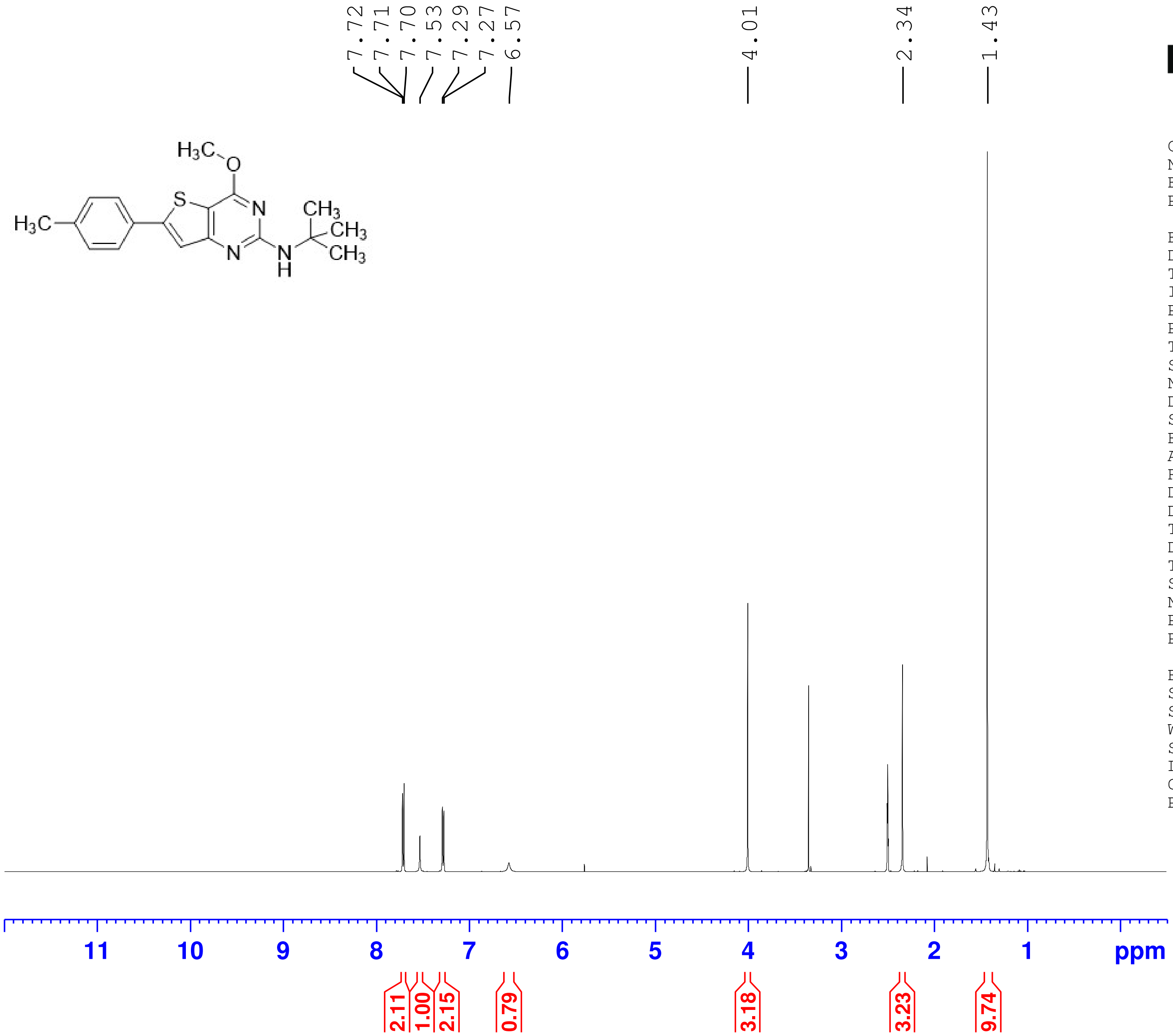

BRUKER

Current Data Parameters	
NAME	LP0062
EXPNO	10
PROCNO	1

F2 - Acquisition Parameters	
Date_	20190527
Time	17.40 h
INSTRUM	Spect
PROBHD	Z136122_0002 (
PULPROG	zg30
TD	65536
SOLVENT	DMSO
NS	16
DS	2
SWH	8012.820 Hz
FIDRES	0.244532 Hz
AQ	4.0894465 sec
RG	10
DW	62.400 usec
DE	10.00 usec
TE	298.0 K
D1	1.50000000 sec
TD0	1
SFO1	500.1735012 MHz
NUC1	1H
P1	12.90 usec
PLW1	7.00000000 W

F2 - Processing parameters	
SI	65536
SF	500.1700095 MHz
WDW	EM
SSB	0
LB	0.10 Hz
GB	0
PC	1.00

LP0062 / DMSO

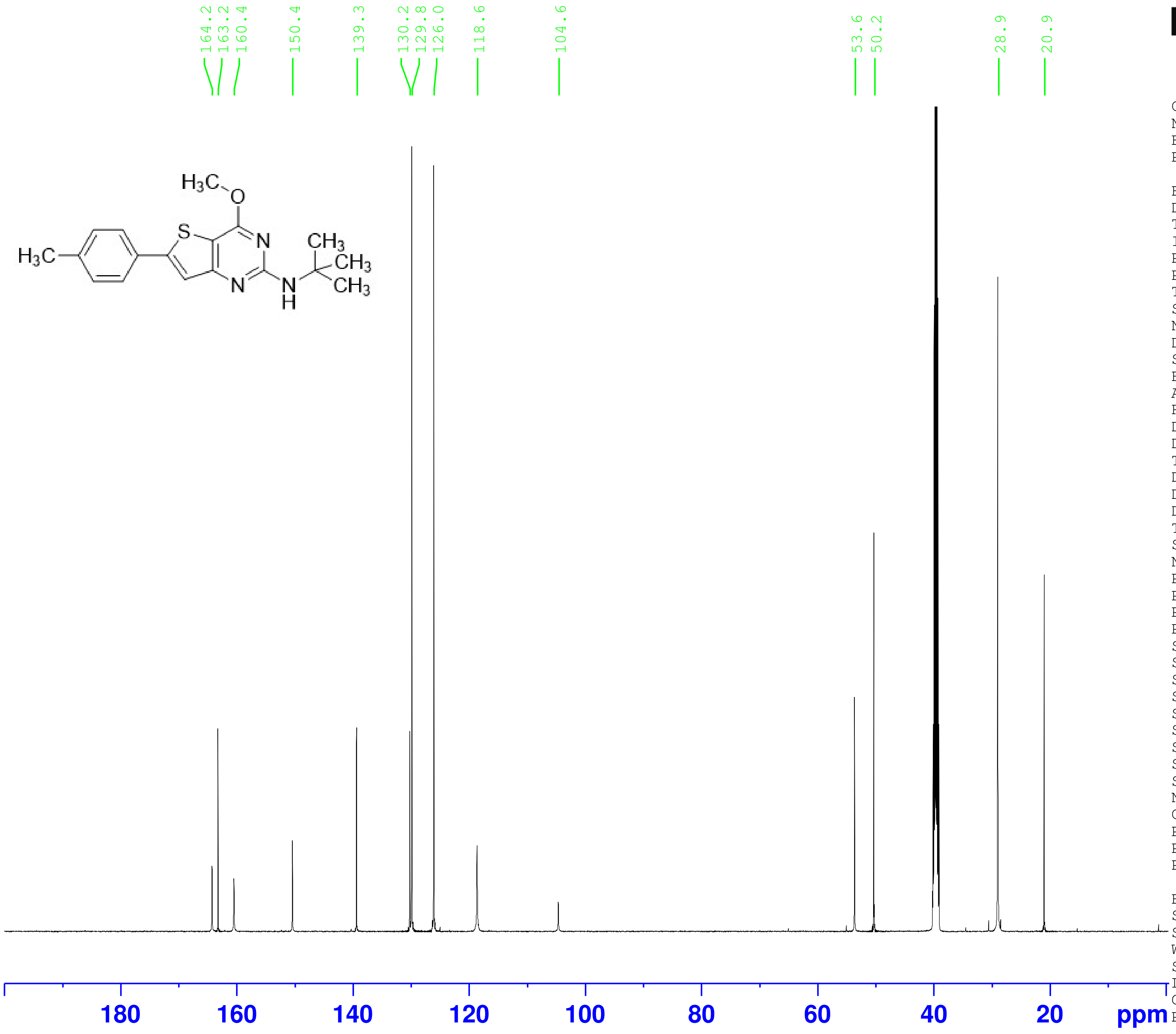

Current Data Parameters
NAME LP0062
EXPNO 11
PROCNO 1

F2 - Acquisition Parameters
Date_ 20190527
Time 20.30 h
INSTRUM Spect
PROBHD Z136122_0002 (
PULPROG udeft
TD 20586
SOLVENT DMSO
NS 2048
DS 0
SWH 28846.154 Hz
FIDRES 2.802502 Hz
AQ 0.3568240 sec
RG 456
DW 17.333 usec
DE 18.00 usec
TE 298.0 K
D1 4.00000000 sec
D12 0.00002000 sec
D20 200.00000000 sec
TD0 1
SFO1 125.7810526 MHz
NUC1 13C
P1 10.00 usec
P13 2000.00 usec
P26 500.00 usec
PLW1 26.00000000 W
SPNAM[5] Crp60comp.4
SPOAL5 0.500
SPOFFS5 0 Hz
SPW5 3.97250009 W
SPNAM[8] Crp60,0.5,20.1
SPOAL8 0.500
SPOFFS8 0 Hz
SPW8 3.97250009 W
SFO2 500.1720007 MHz
NUC2 1H
CPDPRG[2 waltz16
PCPD2 80.00 usec
PLW2 7.00000000 W
PLW12 0.18200999 W

F2 - Processing parameters
SI 32768
SF 125.7679028 MHz
WDW EM
SSB 0
LB 2.00 Hz
GB 0
PC 1.40

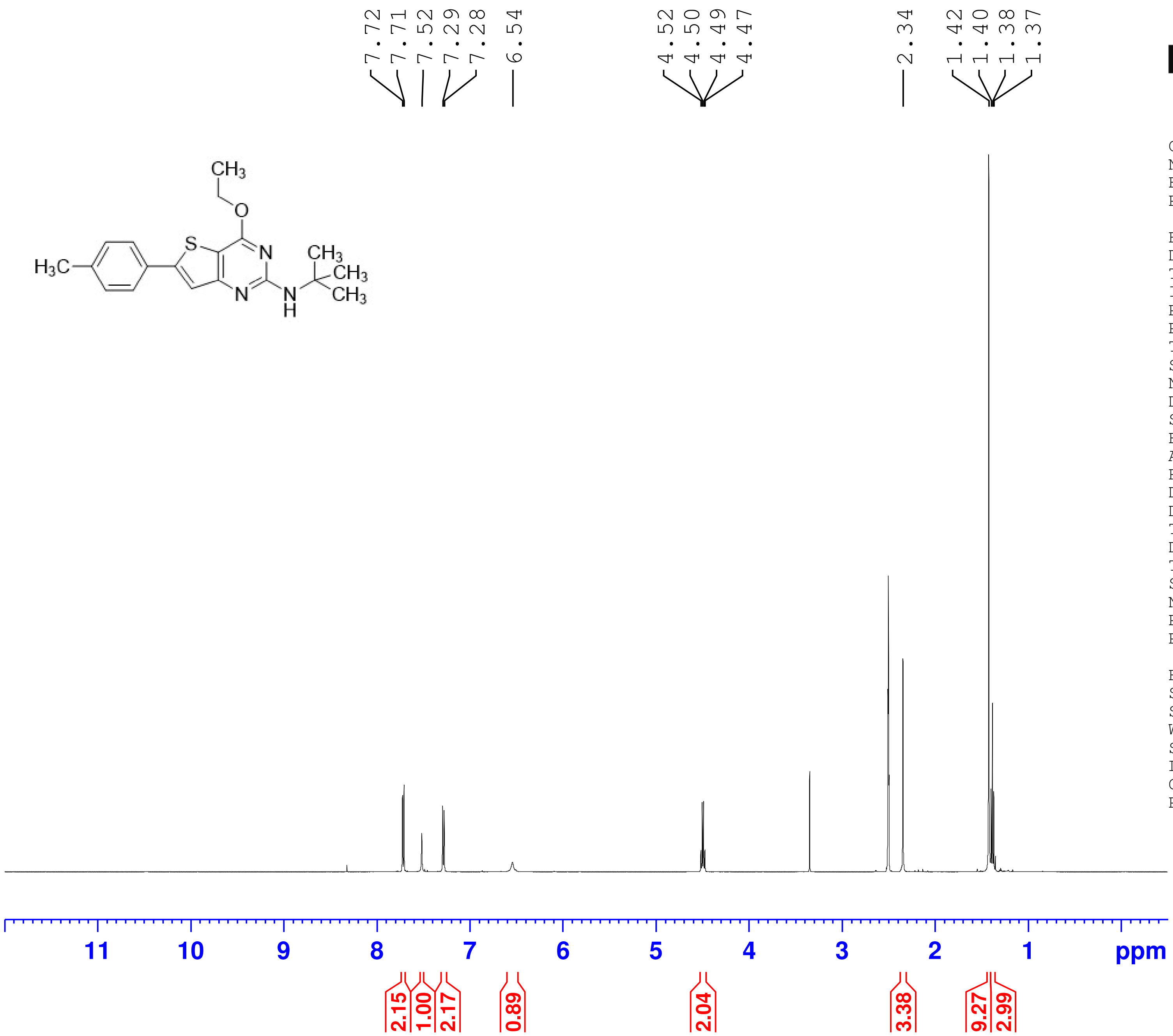

Current Data Parameters

NAME	LP0045
EXPNO	10
PROCNO	1

F2 - Acquisition Parameters

Date_	20190507
Time	16.15 h
INSTRUM	Spect
PROBHD	Z136122_0002 (
PULPROG	zg30
TD	65536
SOLVENT	DMSO
NS	16
DS	2
SWH	8012.820 Hz
FIDRES	0.244532 Hz
AQ	4.0894465 sec
RG	9
DW	62.400 usec
DE	10.00 usec
TE	298.0 K
D1	1.50000000 sec
TD0	1
SFO1	500.1735012 MHz
NUC1	1H
P1	12.90 usec
PLW1	7.00000000 W

F2 - Processing parameters

SI	65536
SF	500.1700076 MHz
WDW	EM
SSB	0
LB	0.10 Hz
GB	0
PC	1.00

LP0045 / DMSO

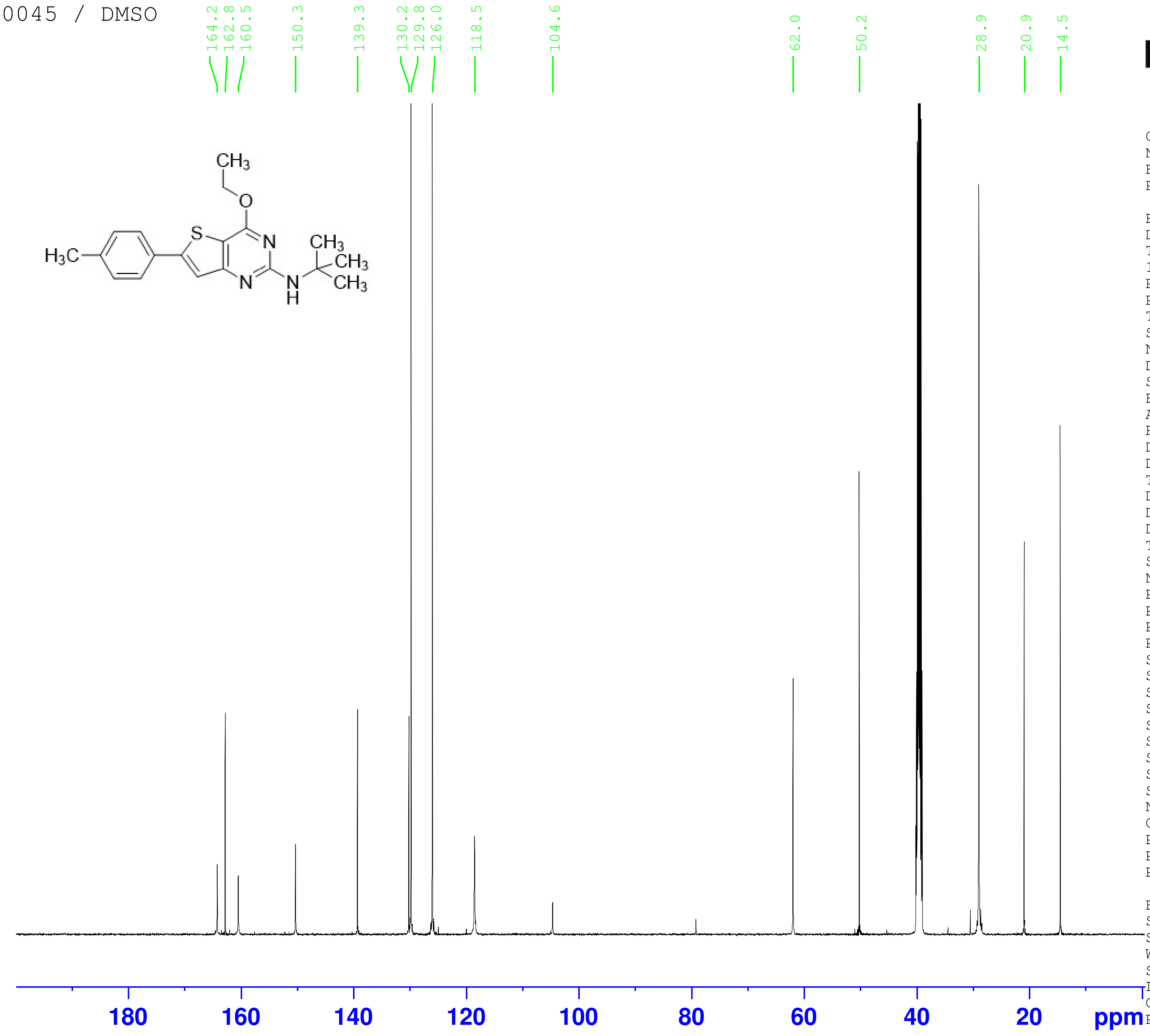

BRUKER

Current Data Parameters
NAME LP0045
EXPNO 11
PROCNO 1

F2 - Acquisition Parameters
Date_ 20190507
Time 18.31 h
INSTRUM Spect
PROBHD Z136122_0002 (
PULPROG udeft
TD 20586
SOLVENT DMSO
NS 2048
DS 0
SWH 28846.154 Hz
FIDRES 2.802502 Hz
AQ 0.3568240 sec
RG 322
DW 17.333 usec
DE 18.00 usec
TE 298.0 K
D1 3.00000000 sec
D12 0.00002000 sec
D20 200.00000000 sec
TD0 1
SFO1 125.7810526 MHz
NUC1 13C
P1 10.00 usec
P13 2000.00 usec
P26 500.00 usec
PLW1 26.00000000 W
SPNAM[5] Crp60comp.4
SPOAL5 0.500
SPOFFS5 0 Hz
SPW5 3.97250009 W
SPNAM[8] Crp60,0.5,20.1
SPOAL8 0.500
SPOFFS8 0 Hz
SPW8 3.97250009 W
SFO2 500.1720007 MHz
NUC2 1H
CPDPRG[2 waltz16
PCPD2 80.00 usec
PLW2 7.00000000 W
PLW12 0.18200999 W

F2 - Processing parameters
SI 32768
SF 125.7679024 MHz
WDW EM
SSB 0
LB 2.00 Hz
GB 0
PC 1.40

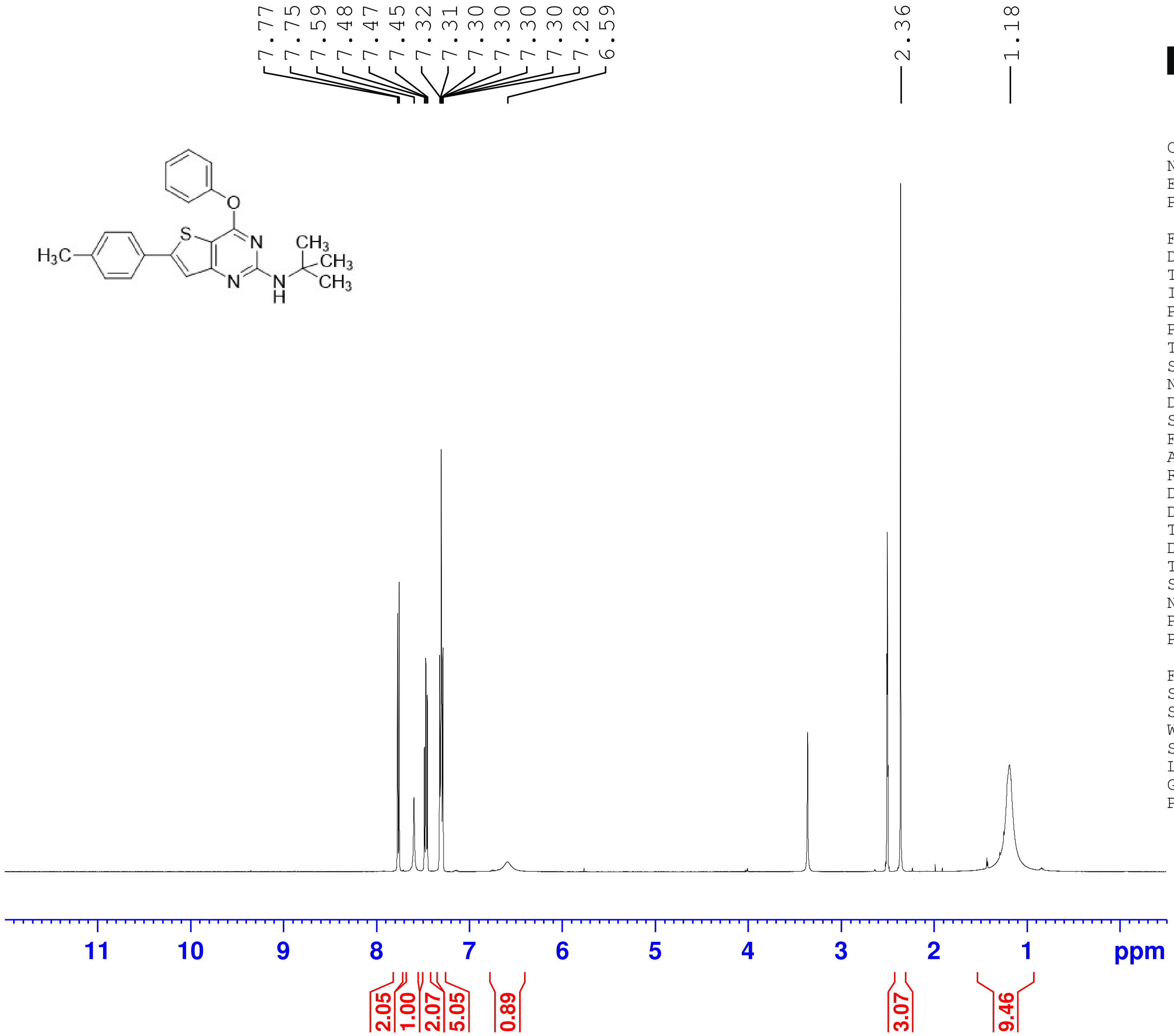


```
Current Data Parameters
NAME                LP0169
EXPNO                   12
PROCNO                   1

F2 - Acquisition Parameters
Date_             20200220
Time                  6.24 h
INSTRUM              Spect
PROBHD    Z136122_0002 (
PULPROG               zg30
TD                   65536
SOLVENT               DMSO
NS                      16
DS                       2
SWH              10000.000 Hz
FIDRES            0.305176 Hz
AQ               3.2767999 sec
RG                      10
DW                  50.000 usec
DE                   10.00 usec
TE                   298.0 K
D1              1.50000000 sec
TD0                      1
SFO1           500.1730885 MHz
NUC1                    1H
P1                   12.90 usec
PLW1            7.00000000 W

F2 - Processing parameters
SI                   65536
SF             500.1700074 MHz
WDW                     EM
SSB                      0
LB                    0.10 Hz
GB                       0
PC                    2.00
```

LP0169 / DMSO

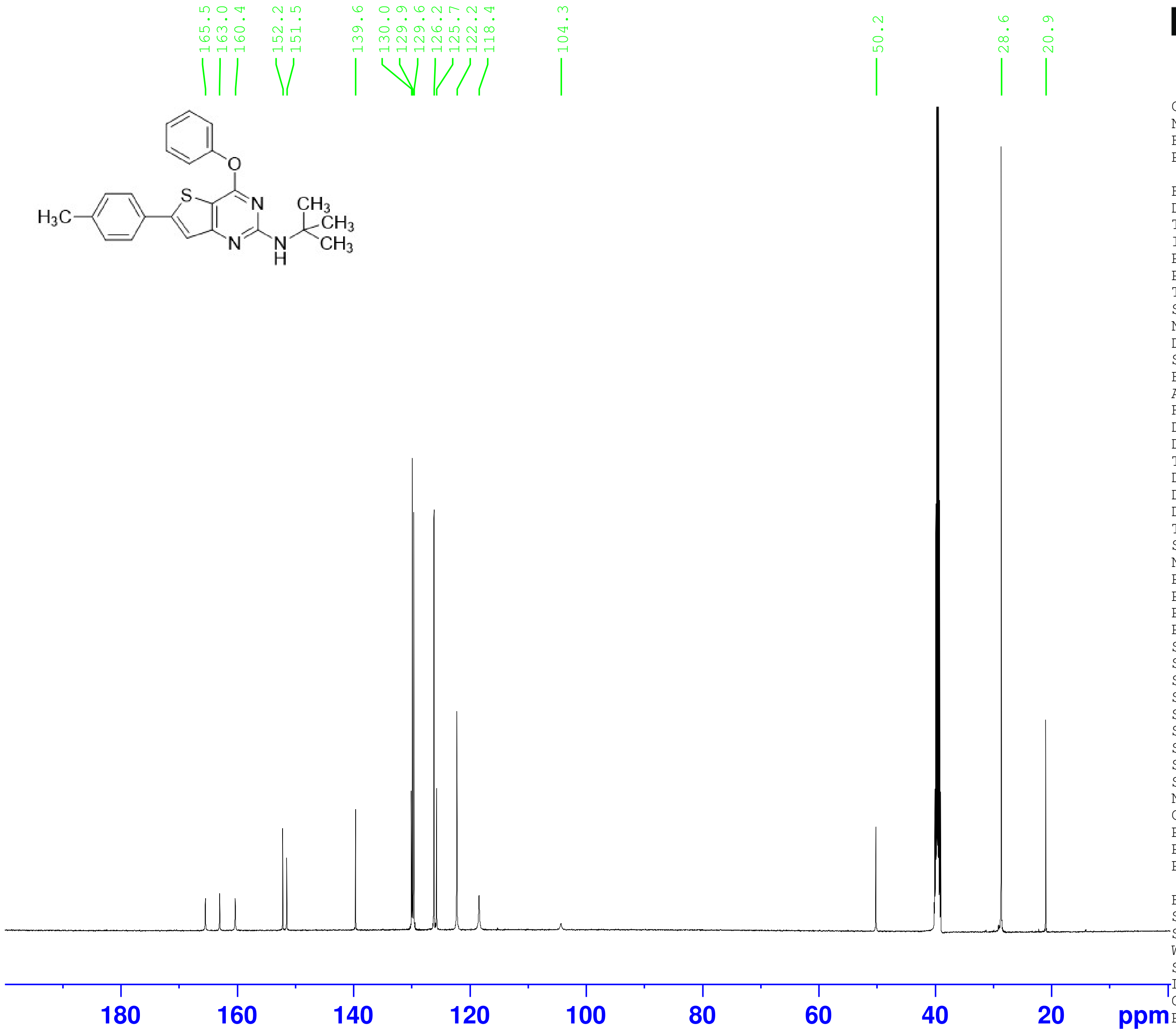

BRUKER

Current Data Parameters
NAME LP0169
EXPNO 13
PROCNO 1

F2 - Acquisition Parameters
Date_ 20200220
Time 8.13 h
INSTRUM Spect
PROBHD Z136122_0002 (
PULPROG udeft
TD 20586
SOLVENT DMSO
NS 1611
DS 0
SWH 28846.154 Hz
FIDRES 2.802502 Hz
AQ 0.3568240 sec
RG 724
DW 17.333 usec
DE 18.00 usec
TE 298.0 K
D1 3.00000000 sec
D12 0.00002000 sec
D20 200.00000000 sec
TD0 1
SFO1 125.7810526 MHz
NUC1 13C
P1 10.00 usec
P13 2000.00 usec
P26 500.00 usec
PLW1 26.00000000 W
SPNAM[5] Crp60comp.4
SPOAL5 0.500
SPOFFS5 0 Hz
SPW5 3.97250009 W
SPNAM[8] Crp60,0.5,20.1
SPOAL8 0.500
SPOFFS8 0 Hz
SPW8 3.97250009 W
SFO2 500.1720007 MHz
NUC2 1H
CPDPRG[2 waltz16
PCPD2 80.00 usec
PLW2 7.00000000 W
PLW12 0.18200999 W

F2 - Processing parameters
SI 32768
SF 125.7679006 MHz
WDW EM
SSB 0
LB 2.00 Hz
GB 0
PC 2.00

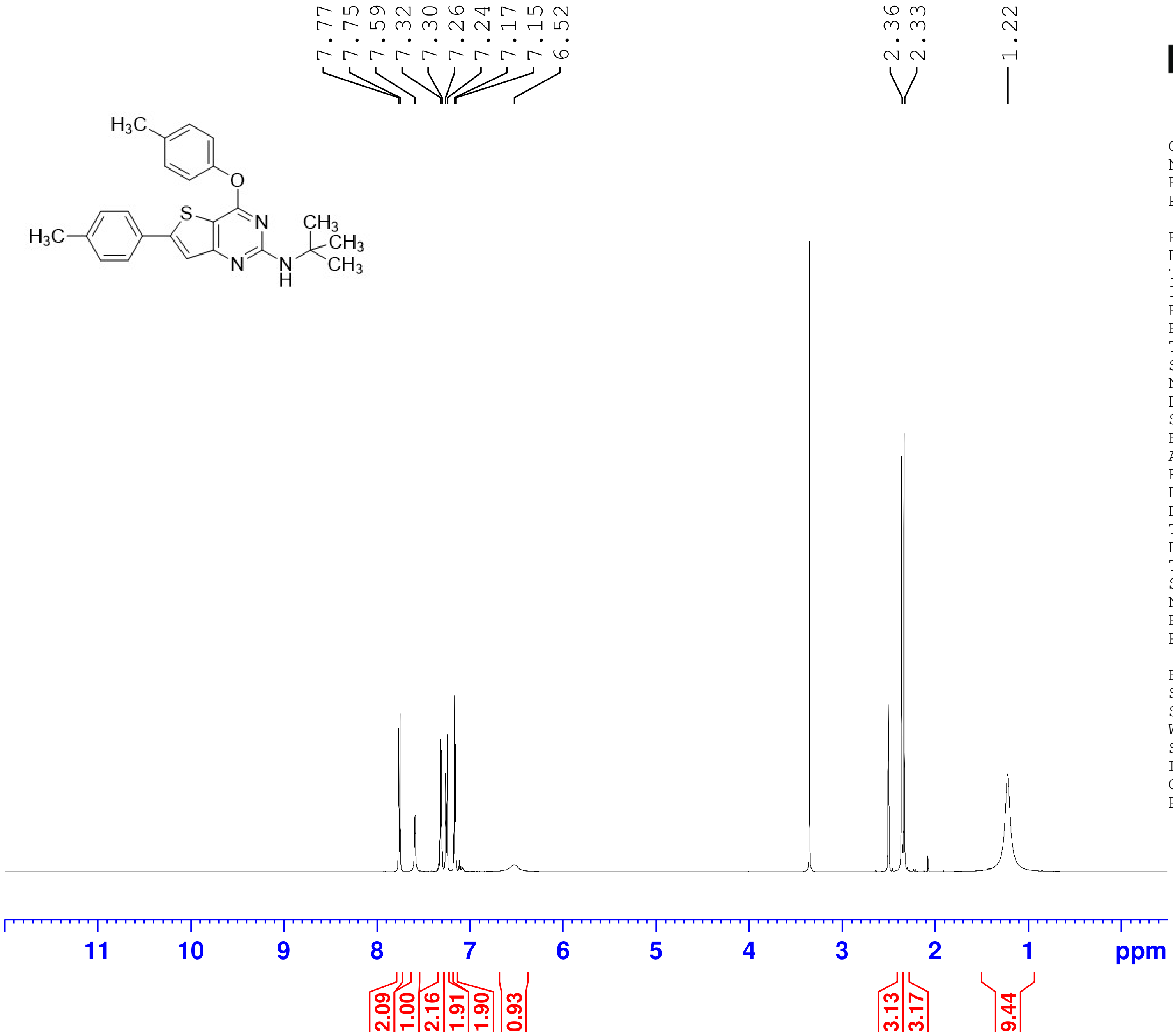

BRUKER

Current Data Parameters

NAME	LP0168
EXPNO	10
PROCNO	1

F2 - Acquisition Parameters

Date_	20200608
Time	9.32 h
INSTRUM	Spect
PROBHD	Z136122_0002 (
PULPROG	zg30
TD	65536
SOLVENT	DMSO
NS	16
DS	2
SWH	10000.000 Hz
FIDRES	0.305176 Hz
AQ	3.2767999 sec
RG	18
DW	50.000 usec
DE	10.00 usec
TE	298.0 K
D1	1.50000000 sec
TD0	1
SFO1	500.1730885 MHz
NUC1	1H
P1	12.90 usec
PLW1	7.00000000 W

F2 - Processing parameters

SI	65536
SF	500.1700096 MHz
WDW	EM
SSB	0
LB	0.10 Hz
GB	0
PC	2.00

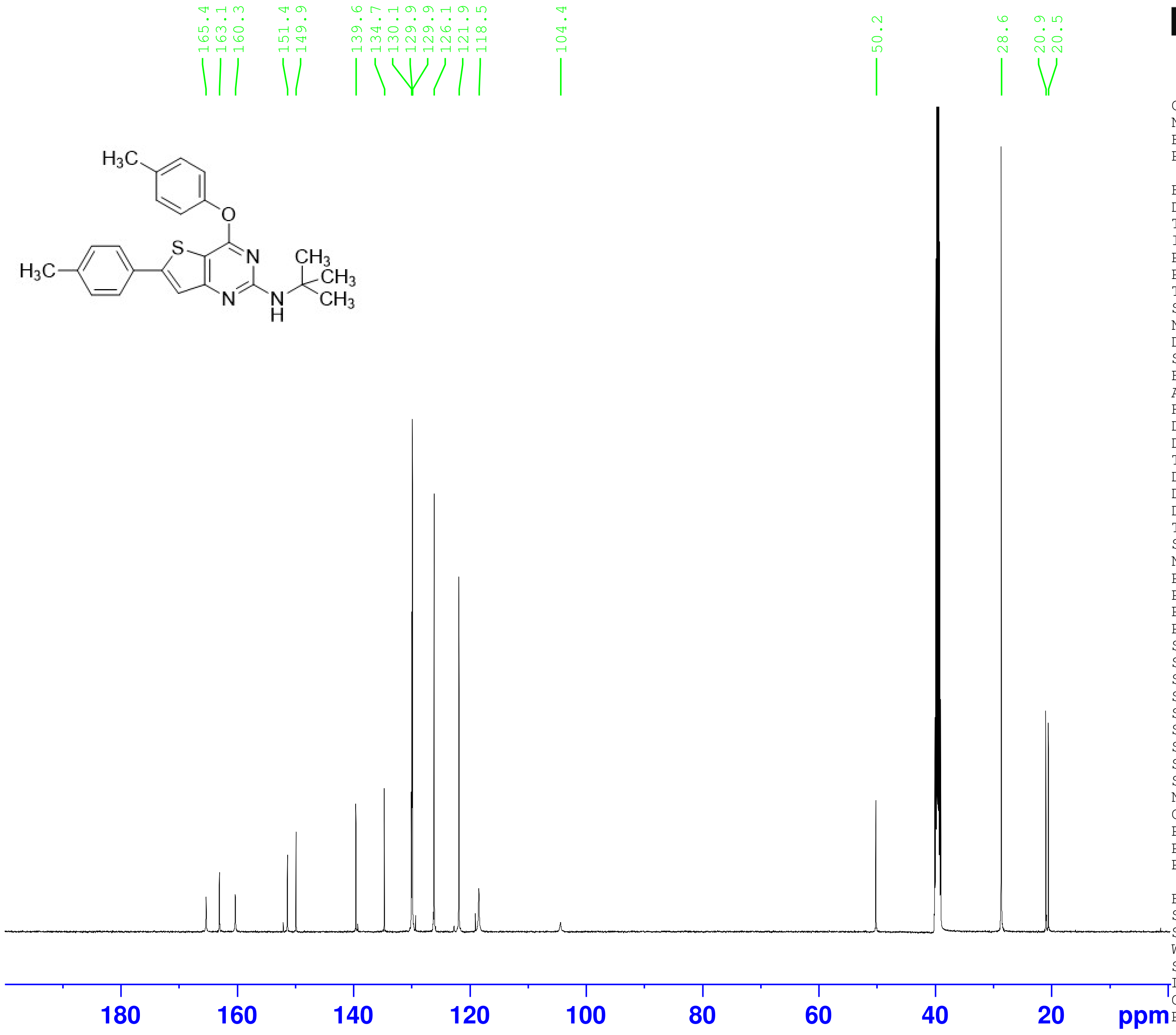

BRUKER

Current Data Parameters
NAME LP0168
EXPNO 11
PROCNO 1

F2 - Acquisition Parameters
Date_ 20200608
Time 11.48 h
INSTRUM Spect
PROBHD Z136122_0002 (
PULPROG udeft
TD 20586
SOLVENT DMSO
NS 2048
DS 0
SWH 28846.154 Hz
FIDRES 2.802502 Hz
AQ 0.3568240 sec
RG 912
DW 17.333 usec
DE 18.00 usec
TE 298.0 K
D1 3.00000000 sec
D12 0.00002000 sec
D20 200.00000000 sec
TD0 1
SFO1 125.7810526 MHz
NUC1 13C
P1 10.00 usec
P13 2000.00 usec
P26 500.00 usec
PLW1 26.00000000 W
SPNAM[5] Crp60comp.4
SPOAL5 0.500
SPOFFS5 0 Hz
SPW5 3.97250009 W
SPNAM[8] Crp60,0.5,20.1
SPOAL8 0.500
SPOFFS8 0 Hz
SPW8 3.97250009 W
SFO2 500.1720007 MHz
NUC2 1H
CPDPRG[2 waltz16
PCPD2 80.00 usec
PLW2 7.00000000 W
PLW12 0.18200999 W

F2 - Processing parameters
SI 32768
SF 125.7679025 MHz
WDW EM
SSB 0
LB 2.00 Hz
GB 0
PC 2.00

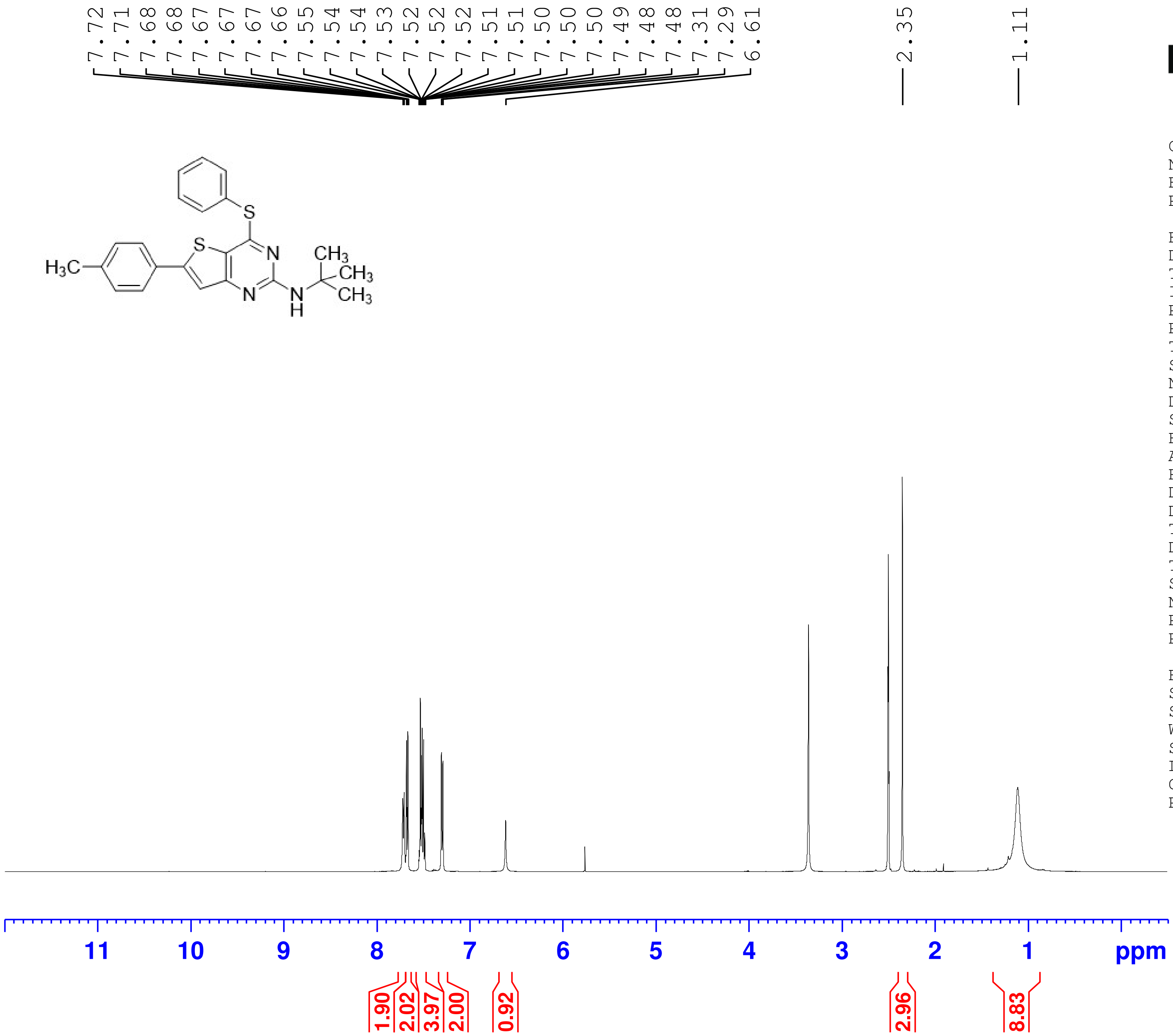

Current Data Parameters

NAME	LP0091
EXPNO	10
PROCNO	1

F2 - Acquisition Parameters

Date_	20190828
Time	16.28 h
INSTRUM	Spect
PROBHD	Z136122_0002 (
PULPROG	zg30
TD	65536
SOLVENT	DMSO
NS	16
DS	2
SWH	8012.820 Hz
FIDRES	0.244532 Hz
AQ	4.0894465 sec
RG	10
DW	62.400 usec
DE	10.00 usec
TE	298.0 K
D1	1.50000000 sec
TD0	1
SFO1	500.1735012 MHz
NUC1	1H
P1	12.90 usec
PLW1	7.00000000 W

F2 - Processing parameters

SI	65536
SF	500.1700077 MHz
WDW	EM
SSB	0
LB	0.10 Hz
GB	0
PC	1.00

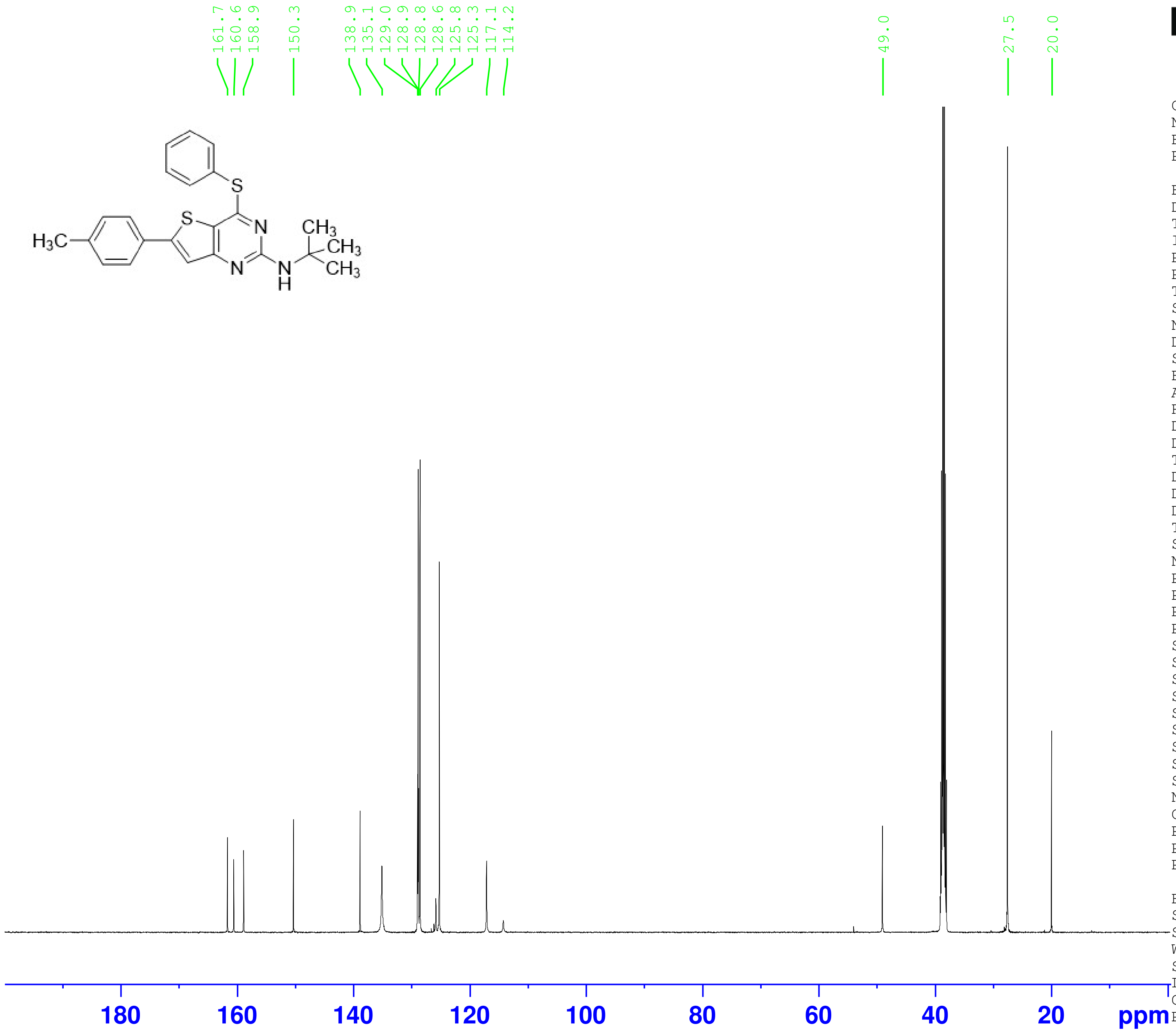

Current Data Parameters
NAME LP0091
EXPNO 11
PROCNO 1

F2 - Acquisition Parameters
Date_ 20190828
Time 18.44 h
INSTRUM Spect
PROBHD Z136122_0002 (
PULPROG udeft
TD 20586
SOLVENT DMSO
NS 2048
DS 0
SWH 28846.154 Hz
FIDRES 2.802502 Hz
AQ 0.3568240 sec
RG 912
DW 17.333 usec
DE 18.00 usec
TE 298.0 K
D1 3.00000000 sec
D12 0.00002000 sec
D20 200.00000000 sec
TD0 1
SFO1 125.7810526 MHz
NUC1 13C
P1 10.00 usec
P13 2000.00 usec
P26 500.00 usec
PLW1 26.00000000 W
SPNAM[5] Crp60comp.4
SPOAL5 0.500
SPOFFS5 0 Hz
SPW5 3.97250009 W
SPNAM[8] Crp60,0.5,20.1
SPOAL8 0.500
SPOFFS8 0 Hz
SPW8 3.97250009 W
SFO2 500.1720007 MHz
NUC2 1H
CPDPRG[2 waltz16
PCPD2 80.00 usec
PLW2 7.00000000 W
PLW12 0.18200999 W

F2 - Processing parameters
SI 32768
SF 125.7680264 MHz
WDW EM
SSB 0
LB 2.00 Hz
GB 0
PC 1.40

LP0090/ DMSO

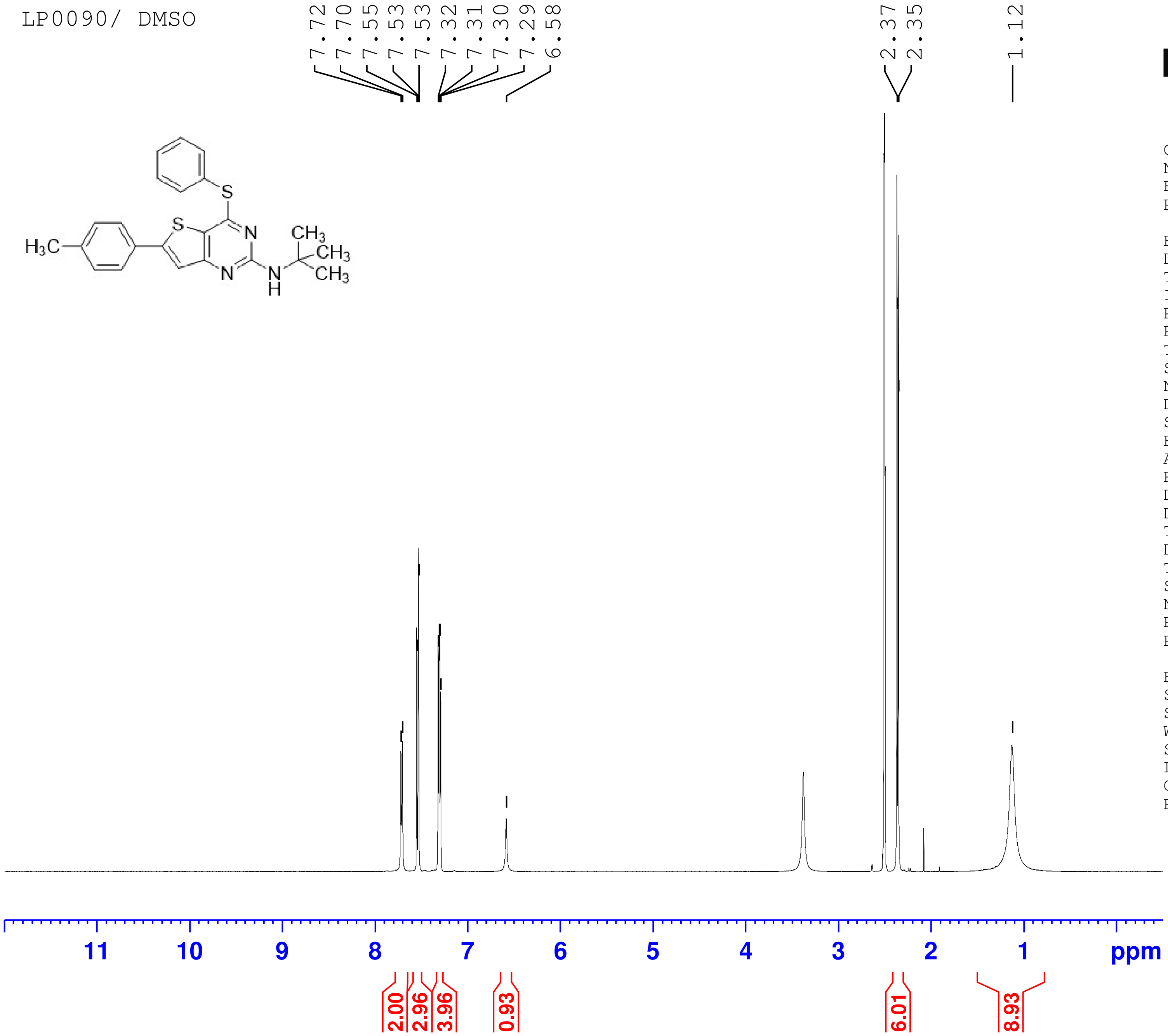

Current Data Parameters
NAME LP0090-recrist
EXPNO 10
PROCNO 1

F2 - Acquisition Parameters
Date_ 20190925
Time 17.36 h
INSTRUM Spect
PROBHD Z136122_0002 (
PULPROG zg30
TD 65536
SOLVENT DMSO
NS 16
DS 2
SWH 8012.820 Hz
FIDRES 0.244532 Hz
AQ 4.0894465 sec
RG 10
DW 62.400 usec
DE 10.00 usec
TE 298.0 K
D1 1.50000000 sec
TD0 1
SFO1 500.1735012 MHz
NUC1 1H
P1 12.90 usec
PLW1 7.00000000 W

F2 - Processing parameters
SI 65536
SF 500.1700073 MHz
WDW EM
SSB 0
LB 0.10 Hz
GB 0
PC 1.00

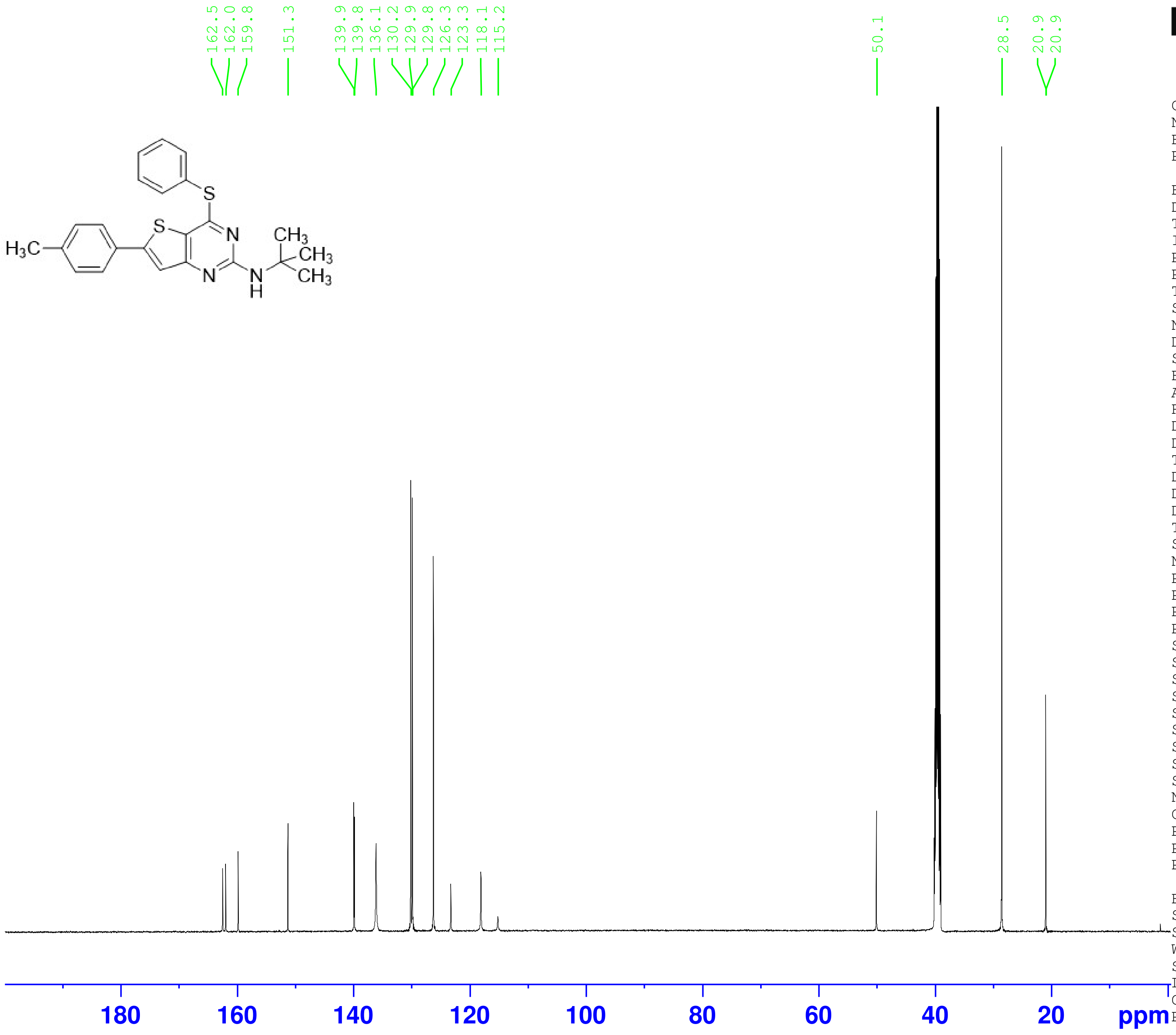

BRUKER

Current Data Parameters

NAME	LP0090-recrist
EXPNO	11
PROCNO	1

F2 - Acquisition Parameters

Date_	20190925
Time	19.53 h
INSTRUM	Spect
PROBHD	Z136122_0002 (
PULPROG	udeft
TD	20586
SOLVENT	DMSO
NS	2048
DS	0
SWH	28846.154 Hz
FIDRES	2.802502 Hz
AQ	0.3568240 sec
RG	912
DW	17.333 usec
DE	18.00 usec
TE	298.0 K
D1	3.00000000 sec
D12	0.00002000 sec
D20	200.00000000 sec
TD0	1
SFO1	125.7810526 MHz
NUC1	13C
P1	10.00 usec
P13	2000.00 usec
P26	500.00 usec
PLW1	26.00000000 W
SPNAM[5]	Crp60comp.4
SPOAL5	0.500
SPOFFS5	0 Hz
SPW5	3.97250009 W
SPNAM[8]	Crp60,0.5,20.1
SPOAL8	0.500
SPOFFS8	0 Hz
SPW8	3.97250009 W
SFO2	500.1720007 MHz
NUC2	1H
CPDPRG[2	waltz16
PCPD2	80.00 usec
PLW2	7.00000000 W
PLW12	0.18200999 W

F2 - Processing parameters

SI	32768
SF	125.7679006 MHz
WDW	EM
SSB	0
LB	2.00 Hz
GB	0
PC	1.40

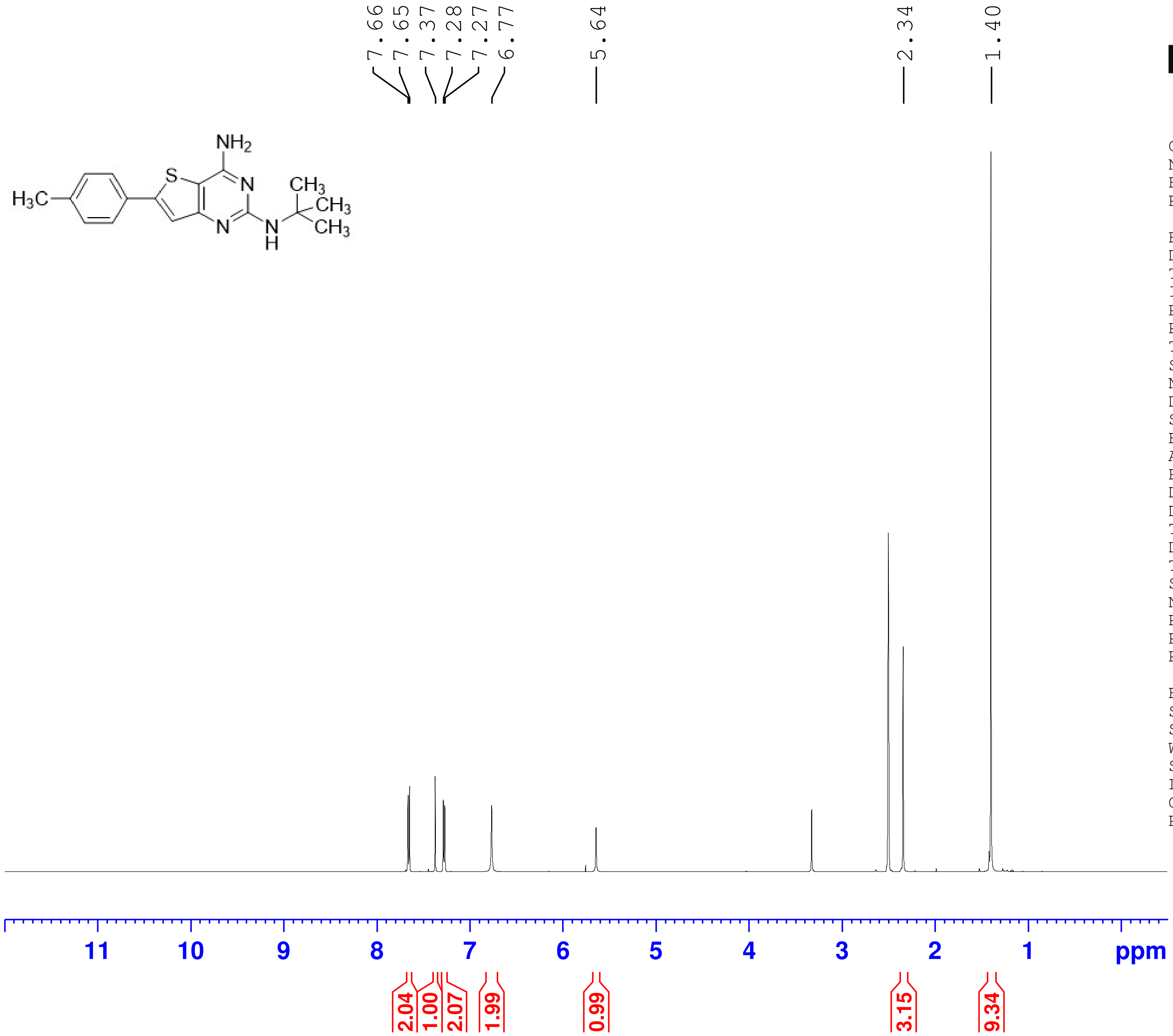

BRUKER

Current Data Parameters
NAME LAG-28-LP0248-F1
EXPNO 10
PROCNO 1

F2 - Acquisition Parameters

Date_	20201127	
Time	19.34	h
INSTRUM	Spect	
PROBHD	Z136122_0002 (	
PULPROG	zg30	
TD	65536	
SOLVENT	DMSO	
NS	16	
DS	2	
SWH	10000.000	Hz
FIDRES	0.305176	Hz
AQ	3.2767999	sec
RG	10	
DW	50.000	usec
DE	10.00	usec
TE	298.0	K
D1	1.50000000	sec
TD0	1	
SFO1	500.1730885	MHz
NUC1	1H	
P0	4.30	usec
P1	12.90	usec
PLW1	7.00000000	W

F2 - Processing parameters

SI	65536	
SF	500.1700073	MHz
WDW	EM	
SSB	0	
LB	0.10	Hz
GB	0	
PC	2.00	

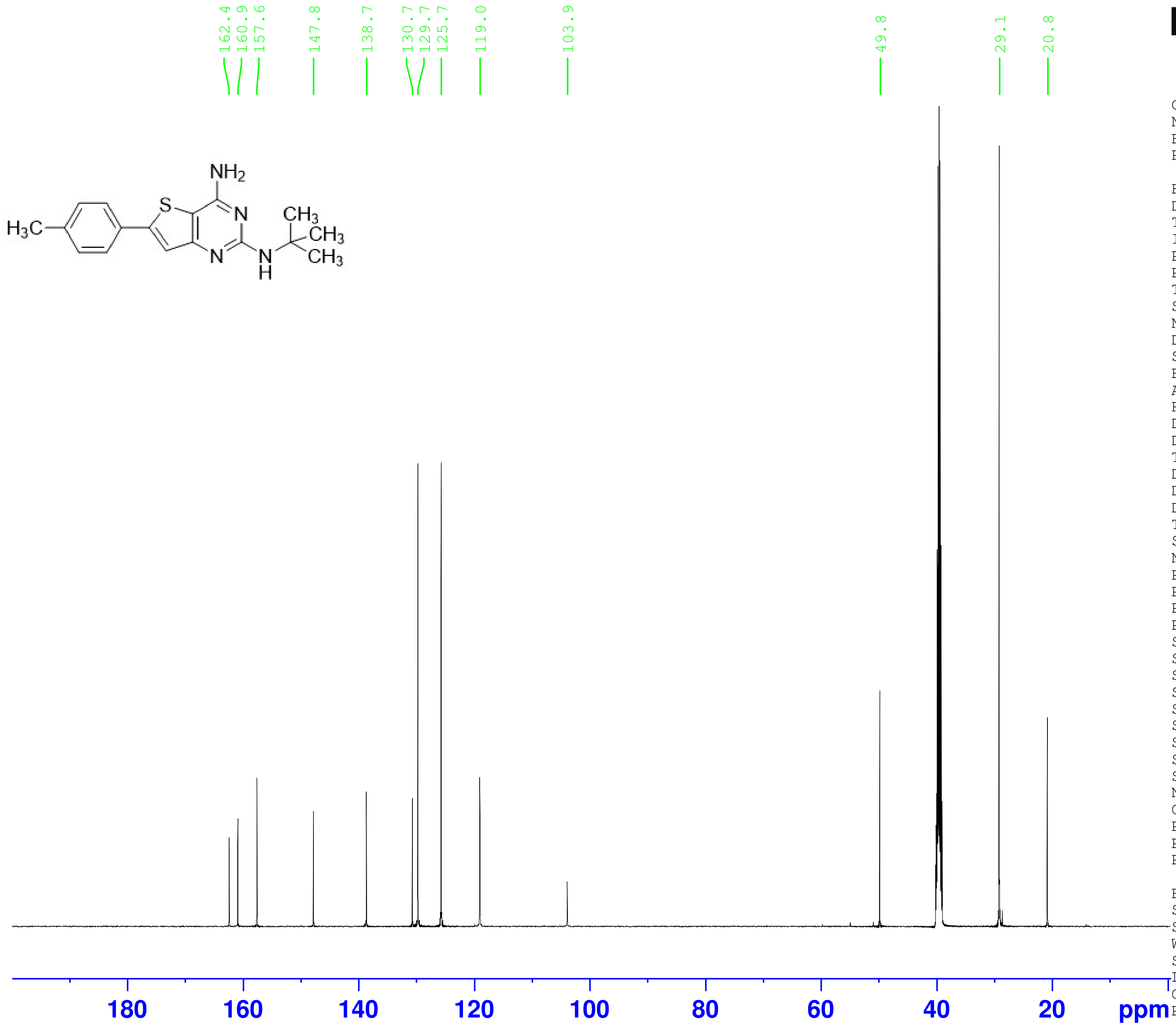

Current Data Parameters
NAME LAG-28-LP0248-F1
EXPNO 11
PROCNO 1

F2 - Acquisition Parameters

Date_	20201127
Time	21.50 h
INSTRUM	Spect
PROBHD	Z136122_0002 (
PULPROG	udeft
TD	16384
SOLVENT	DMSO
NS	2048
DS	0
SWH	28846.154 Hz
FIDRES	3.521259 Hz
AQ	0.2839893 sec
RG	645
DW	17.333 usec
DE	18.00 usec
TE	298.0 K
D1	3.00000000 sec
D12	0.00002000 sec
D20	200.00000000 sec
TD0	1
SFO1	125.7810526 MHz
NUC1	13C
P1	10.00 usec
P13	2000.00 usec
P26	500.00 usec
PLW1	26.00000000 W
SPNAM[5]	Crp60comp.4
SPOAL5	0.500
SPOFFS5	0 Hz
SPW5	3.97250009 W
SPNAM[8]	Crp60,0.5,20.1
SPOAL8	0.500
SPOFFS8	0 Hz
SPW8	3.97250009 W
SFO2	500.1720007 MHz
NUC2	1H
CPDPRG[2	waltz16
PCPD2	80.00 usec
PLW2	7.00000000 W
PLW12	0.18200999 W

F2 - Processing parameters

SI	32768
SF	125.7679077 MHz
WDW	EM
SSB	0
LB	2.00 Hz
GB	0
PC	2.00

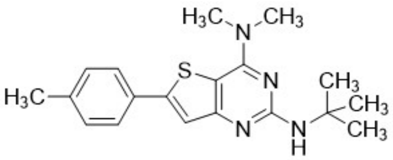

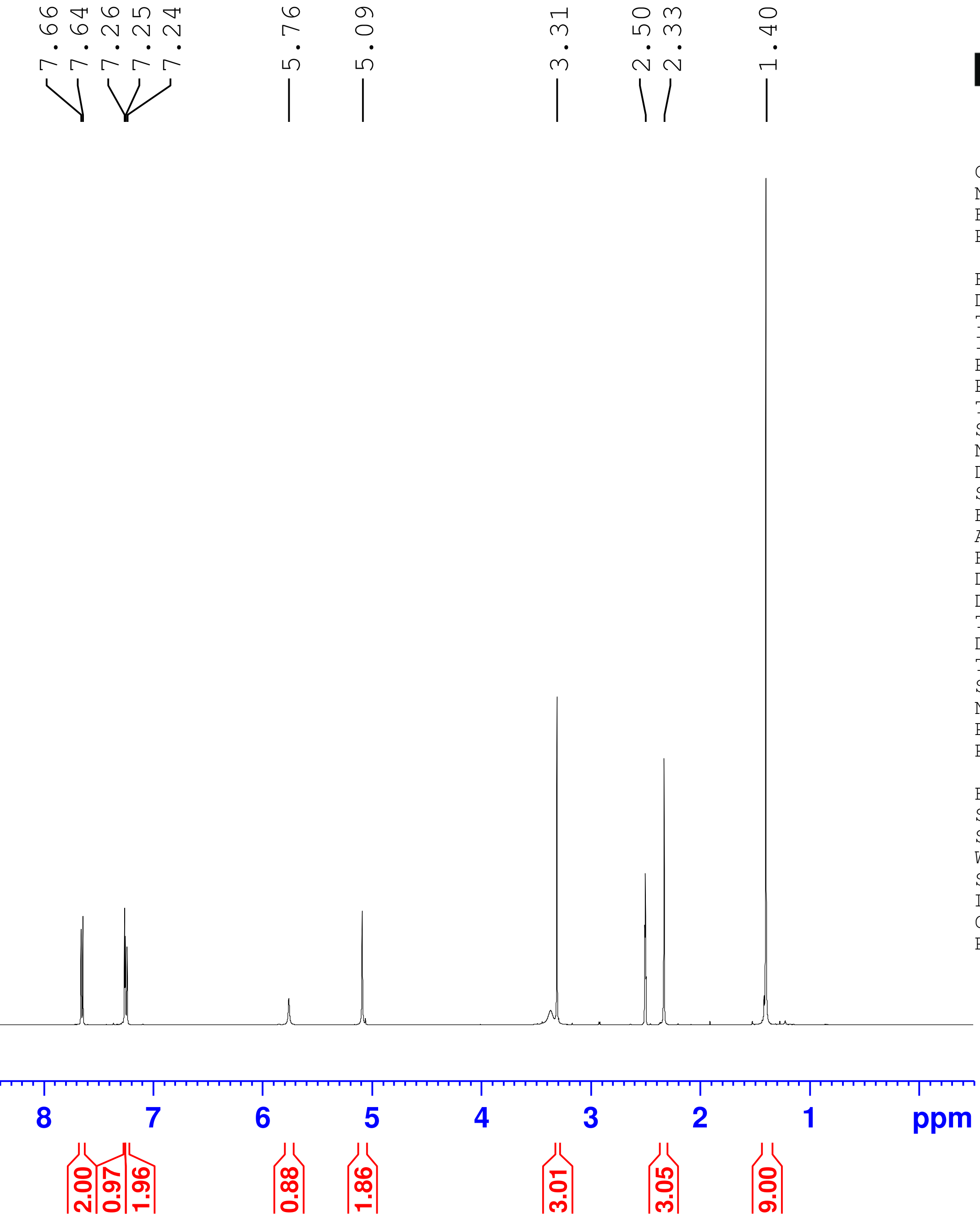

Current Data Parameters
NAME LP0081
EXPNO 10
PROCNO 1

F2 - Acquisition Parameters
Date_ 20190724
Time 11.03 h
INSTRUM Spect
PROBHD Z136122_0002 (
PULPROG zg30
TD 65536
SOLVENT DMSO
NS 16
DS 2
SWH 8012.820 Hz
FIDRES 0.244532 Hz
AQ 4.0894465 sec
RG 9
DW 62.400 usec
DE 10.00 usec
TE 298.0 K
D1 1.50000000 sec
TD0 1
SFO1 500.1735012 MHz
NUC1 1H
P1 12.90 usec
PLW1 7.00000000 W

F2 - Processing parameters
SI 65536
SF 500.1700093 MHz
WDW EM
SSB 0
LB 0.10 Hz
GB 0
PC 1.00

LP0081 / DMSO

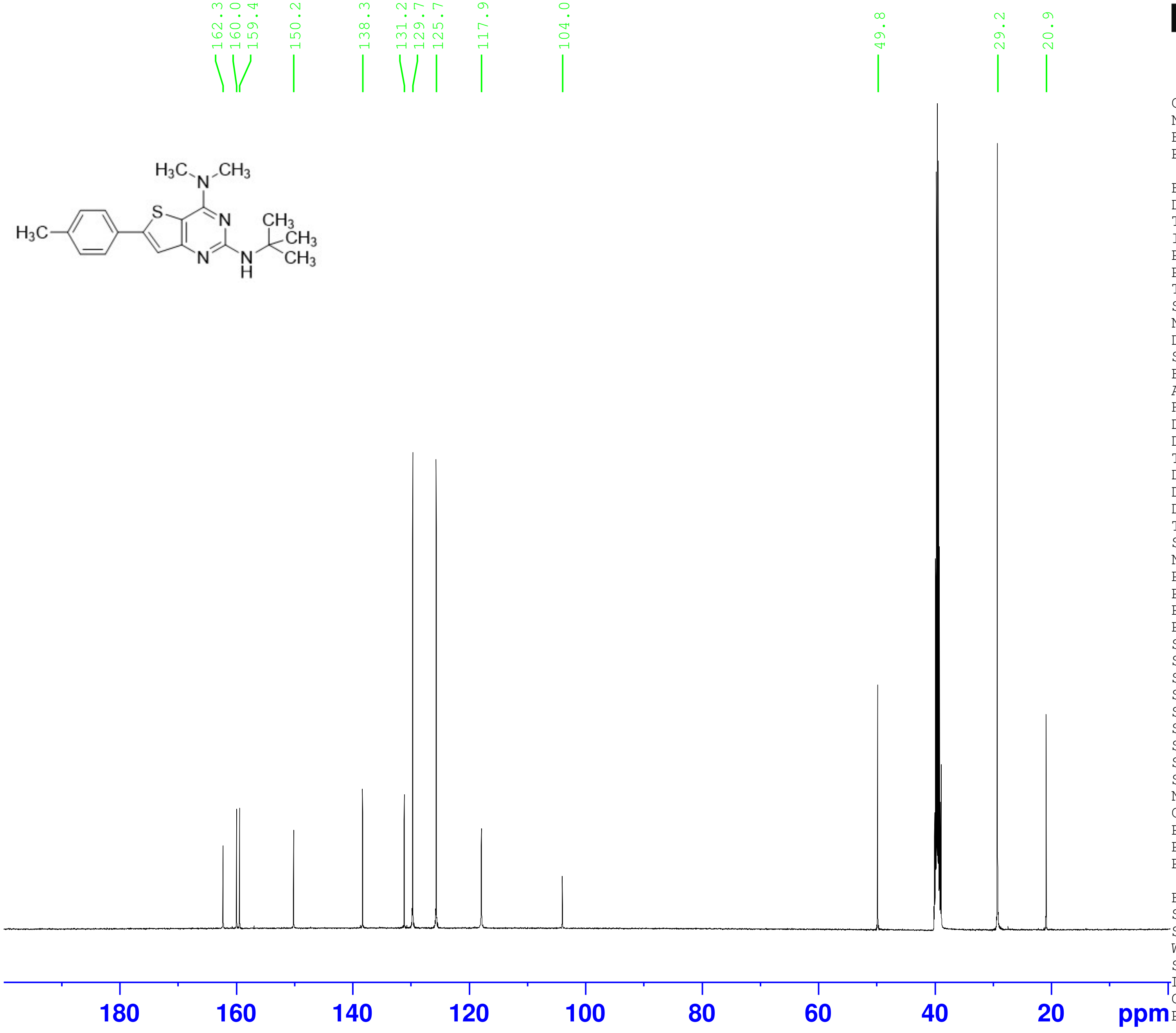

Current Data Parameters
NAME LP0081
EXPNO 11
PROCNO 1

F2 - Acquisition Parameters
Date_ 20190724
Time 12.04 h
INSTRUM Spect
PROBHD Z136122_0002 (
PULPROG udeft
TD 20586
SOLVENT DMSO
NS 842
DS 0
SWH 28846.154 Hz
FIDRES 2.802502 Hz
AQ 0.3568240 sec
RG 406
DW 17.333 usec
DE 18.00 usec
TE 298.0 K
D1 3.00000000 sec
D12 0.00002000 sec
D20 200.00000000 sec
TD0 1
SFO1 125.7810526 MHz
NUC1 13C
P1 10.00 usec
P13 2000.00 usec
P26 500.00 usec
PLW1 26.00000000 W
SPNAM[5] Crp60comp.4
SPOAL5 0.500
SPOFFS5 0 Hz
SPW5 3.97250009 W
SPNAM[8] Crp60,0.5,20.1
SPOAL8 0.500
SPOFFS8 0 Hz
SPW8 3.97250009 W
SFO2 500.1720007 MHz
NUC2 1H
CPDPRG[2 waltz16
PCPD2 80.00 usec
PLW2 7.00000000 W
PLW12 0.18200999 W

F2 - Processing parameters
SI 32768
SF 125.7679015 MHz
WDW EM
SSB 0
LB 2.00 Hz
GB 0
PC 1.40

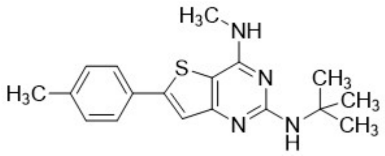

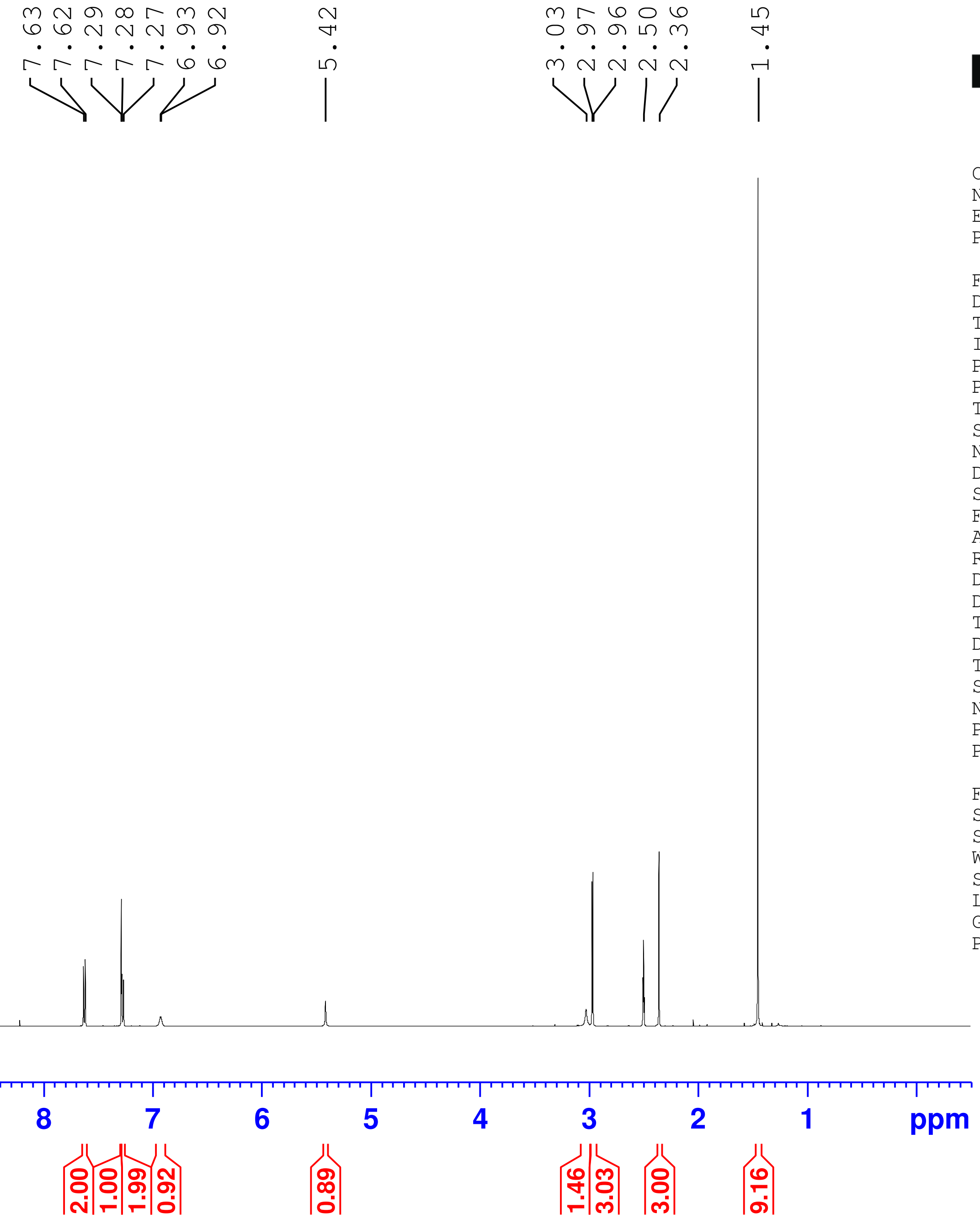

Current Data Parameters

NAME	LP0037
EXPNO	10
PROCNO	1

F2 - Acquisition Parameters

Date_	20190605
Time	11.29 h
INSTRUM	Spect
PROBHD	Z136122_0002 (
PULPROG	zg30
TD	65536
SOLVENT	DMSO
NS	16
DS	2
SWH	8012.820 Hz
FIDRES	0.244532 Hz
AQ	4.0894465 sec
RG	12.7
DW	62.400 usec
DE	10.00 usec
TE	298.0 K
D1	1.50000000 sec
TD0	1
SFO1	500.1735012 MHz
NUC1	1H
P1	12.90 usec
PLW1	7.00000000 W

F2 - Processing parameters

SI	65536
SF	500.1700079 MHz
WDW	EM
SSB	0
LB	0.10 Hz
GB	0
PC	1.00

LP0037 / DMSO

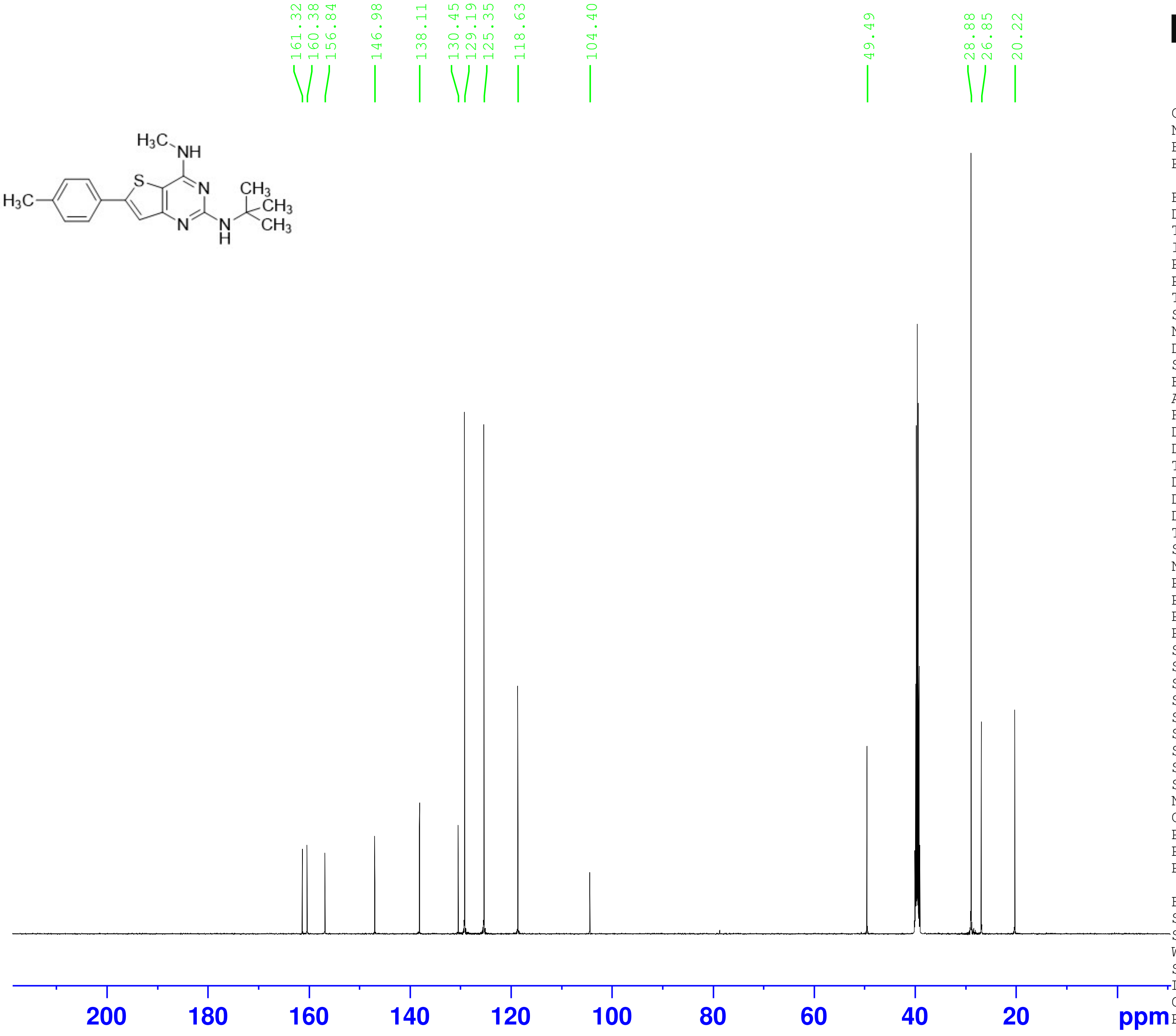

Current Data Parameters

NAME	LP0037
EXPNO	13
PROCNO	1

F2 - Acquisition Parameters

Date_	20190605
Time	12.59 h
INSTRUM	Spect
PROBHD	Z136122_0002 (
PULPROG	udeft
TD	20586
SOLVENT	DMSO
NS	1024
DS	0
SWH	28846.154 Hz
FIDRES	2.802502 Hz
AQ	0.3568240 sec
RG	645
DW	17.333 usec
DE	18.00 usec
TE	298.0 K
D1	4.00000000 sec
D12	0.00002000 sec
D20	200.00000000 sec
TD0	1
SFO1	125.7810526 MHz
NUC1	13C
P1	10.00 usec
P13	2000.00 usec
P26	500.00 usec
PLW1	26.00000000 W
SPNAM[5]	Crp60comp.4
SPOAL5	0.500
SPOFFS5	0 Hz
SPW5	3.97250009 W
SPNAM[8]	Crp60,0.5,20.1
SPOAL8	0.500
SPOFFS8	0 Hz
SPW8	3.97250009 W
SFO2	500.1720007 MHz
NUC2	1H
CPDPRG[2	waltz16
PCPD2	80.00 usec
PLW2	7.00000000 W
PLW12	0.18200999 W

F2 - Processing parameters

SI	32768
SF	125.7679658 MHz
WDW	EM
SSB	0
LB	2.00 Hz
GB	0
PC	1.40

LP0201

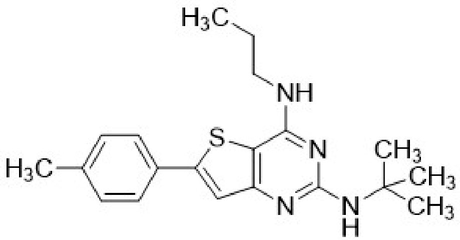

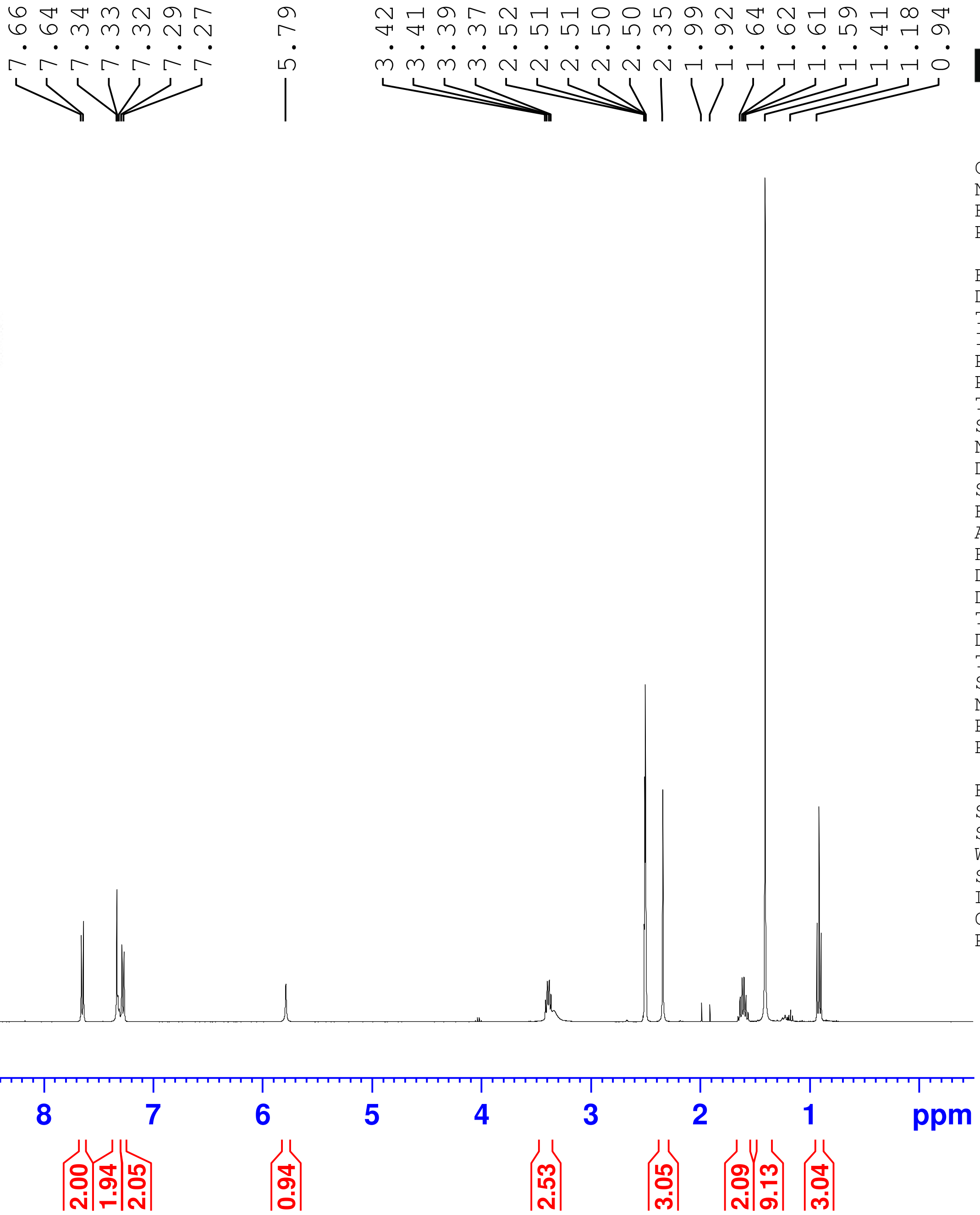

Current Data Parameters

NAME	LP0201
EXPNO	10
PROCNO	1

F2 - Acquisition Parameters

Date_	20200907
Time	22.26 h
INSTRUM	spect
PROBHD	Z104450_0260 (
PULPROG	zg30
TD	65536
SOLVENT	DMSO
NS	16
DS	2
SWH	8012.820 Hz
FIDRES	0.244532 Hz
AQ	4.0894465 sec
RG	140.32
DW	62.400 usec
DE	6.50 usec
TE	298.0 K
D1	1.50000000 sec
TD0	1
SFO1	400.1324710 MHz
NUC1	1H
P1	15.00 usec
PLW1	7.97049999 W

F2 - Processing parameters

SI	65536
SF	400.1300032 MHz
WDW	EM
SSB	0
LB	0.10 Hz
GB	0
PC	4.00

LP0201

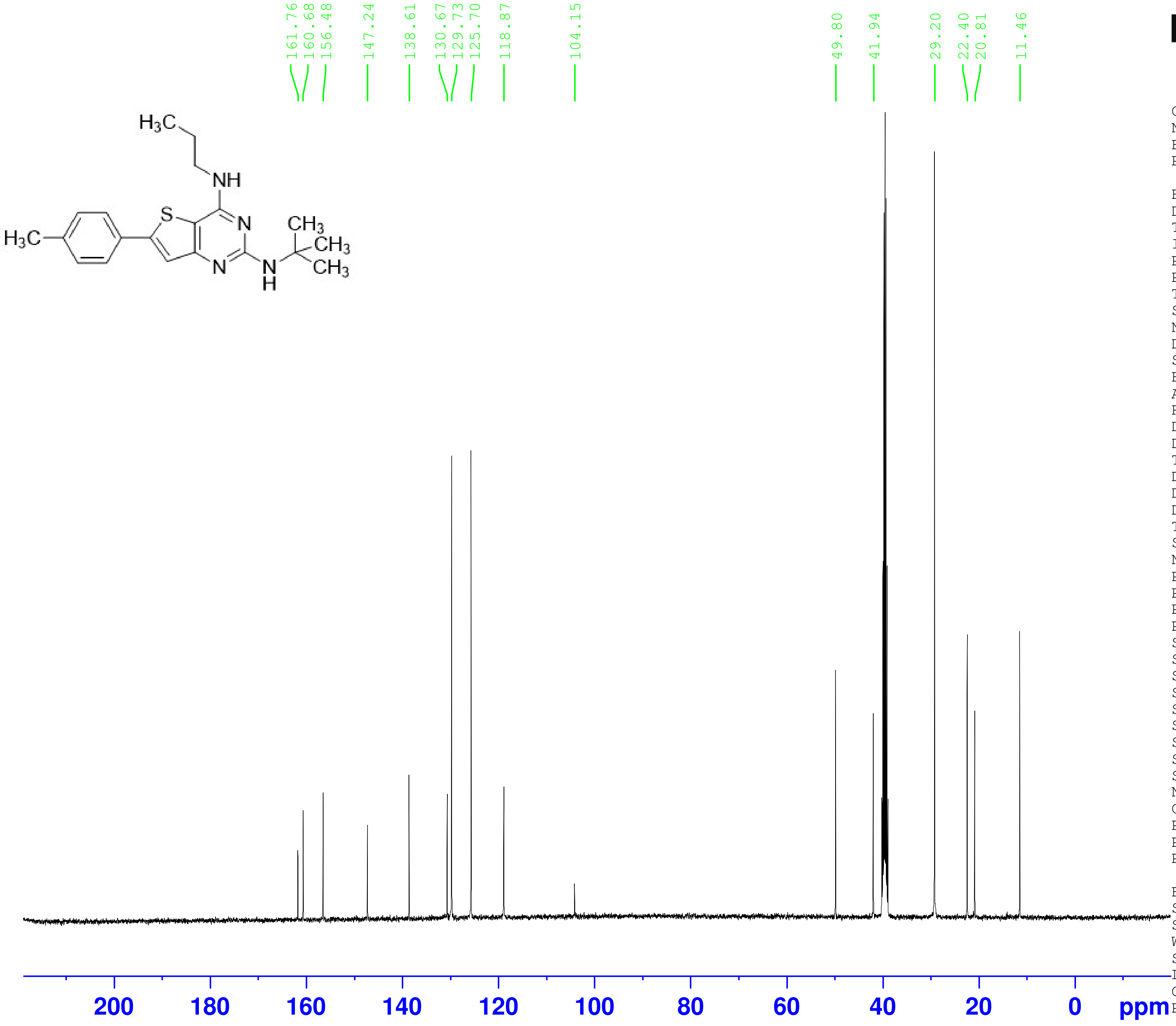

Current Data Parameters	
NAME	LP0201
EXPNO	15
PROCNO	1
F2 - Acquisition Parameters	
Date_	20200908
Time	5.37 h
INSTRUM	spect
PROBHD	Z104450_0260 (
PULPROG	udeft
TD	17304
SOLVENT	DMSO
NS	6144
DS	0
SWH	24038.461 Hz
FIDRES	2.778370 Hz
AQ	0.3599232 sec
RG	196.32
DW	20.800 usec
DE	6.50 usec
TE	298.1 K
D1	3.00000000 sec
D12	0.00002000 sec
D20	200.00000000 sec
TD0	1
SFO1	100.6228293 MHz
NUC1	13C
P1	10.00 usec
P13	2000.00 usec
P26	500.00 usec
PLW1	54.66600037 W
SPNAM[5]	Crp60comp.4
SPOAL5	0.500
SPOFFS5	0 Hz
SPW5	8.35229969 W
SPNAM[8]	Crp60,0.5,20.1
SPOAL8	0.500
SPOFFS8	0 Hz
SPW8	8.35229969 W
SFO2	400.1316005 MHz
NUC2	1H
CPDPRG[2	waltz16
PCPD2	90.00 usec
PLW2	7.97049999 W
PLW12	0.22119720 W
F2 - Processing parameters	
SI	32768
SF	100.6128171 MHz
WDW	EM
SSB	0
LB	2.00 Hz
GB	0
PC	1.40

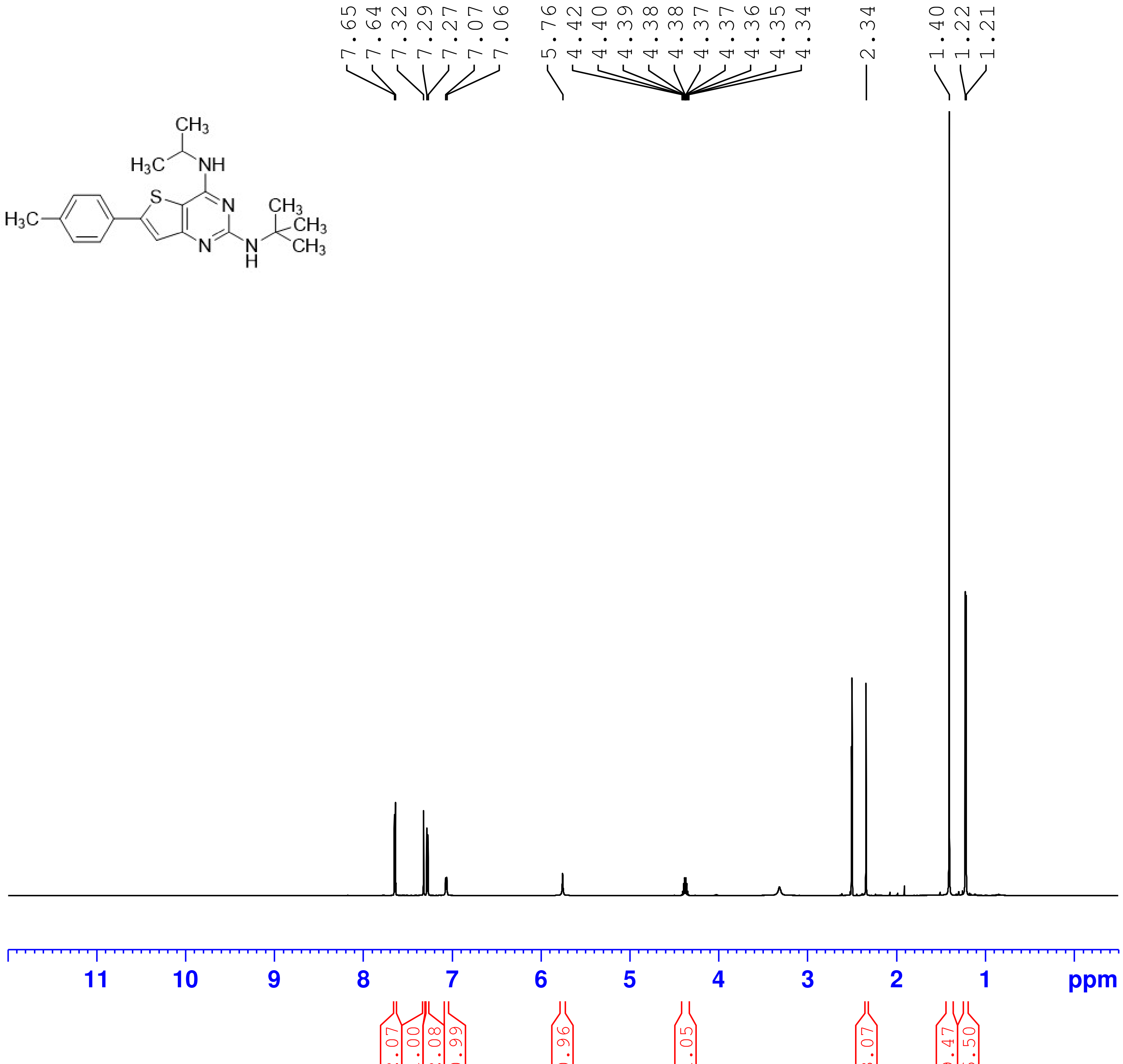

Current Data Parameters
NAME LP0202
EXPNO 10
PROCNO 1

F2 - Acquisition Parameters
Date_ 20200904
Time 3.36 h
INSTRUM spect
PROBHD Z129773_0037 (
PULPROG zg30
TD 65536
SOLVENT DMSO
NS 16
DS 2
SWH 9615.385 Hz
FIDRES 0.293438 Hz
AQ 3.4078720 sec
RG 64
DW 52.000 usec
DE 21.36 usec
TE 298.0 K
D1 1.50000000 sec
TD0 1
SFO1 600.2742019 MHz
NUC1 1H
P0 2.67 usec
P1 8.00 usec
PLW1 13.31200027 W

F2 - Processing parameters
SI 32768
SF 600.2700048 MHz
WDW EM
SSB 0
LB 0 Hz
GB 0
PC 1.00

LP0202 / DMSO

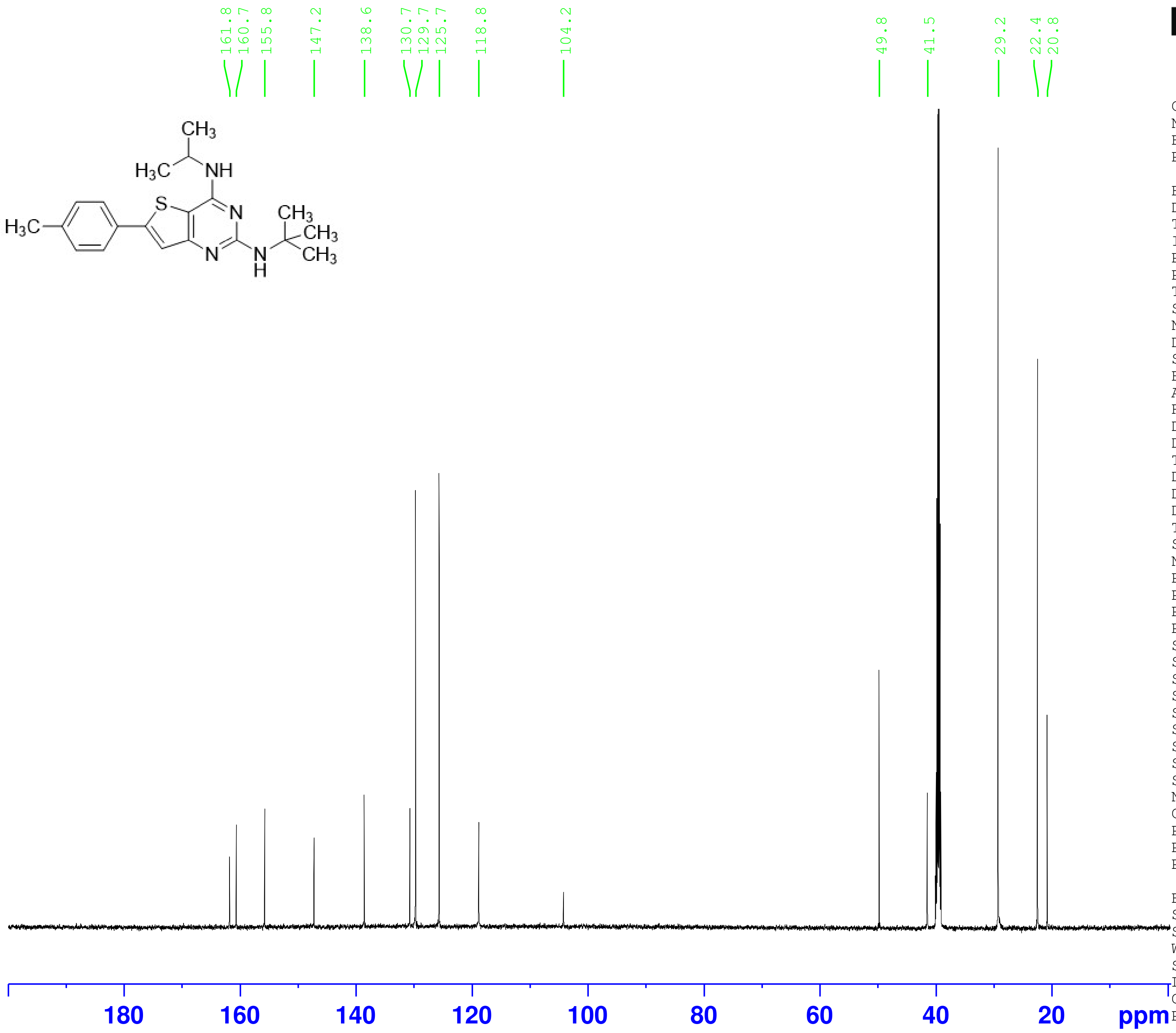


```
Current Data Parameters
NAME              LP0202
EXPNO                 12
PROCNO                 1

F2 - Acquisition Parameters
Date_           20200904
Time                4.52 h
INSTRUM            spect
PROBHD     Z129773_0037 (
PULPROG            udeft
TD                 23816
SOLVENT             DMSO
NS                  1024
DS                     0
SWH            33333.332 Hz
FIDRES          2.799239 Hz
AQ             0.3572400 sec
RG                  2050
DW                15.000 usec
DE                 18.00 usec
TE                 298.0 K
D1            3.00000000 sec
D12           0.00002000 sec
D20         200.00000000 sec
TD0                    1
SFO1         150.9531058 MHz
NUC1                 13C
P1                 12.00 usec
P13              2000.00 usec
P26               500.00 usec
PLW1        185.00000000 W
SPNAM[5]     Crp60comp.4
SPOAL5             0.500
SPOFFS5   0 Hz
SPW5         40.70299911 W
SPNAM[8] Crp60,0.5,20.1
SPOAL8             0.500
SPOFFS8   0 Hz
SPW8         40.70299911 W
SFO2         600.2724011 MHz
NUC2                  1H
CPDPRG[2         waltz16
PCPD2              70.00 usec
PLW2         13.31200027 W
PLW12         0.17387000 W

F2 - Processing parameters
SI                 32768
SF           150.9380872 MHz
WDW                   EM
SSB       0
LB                  2.00 Hz
GB        0
PC                  1.40
```

LP0113 /DMSO

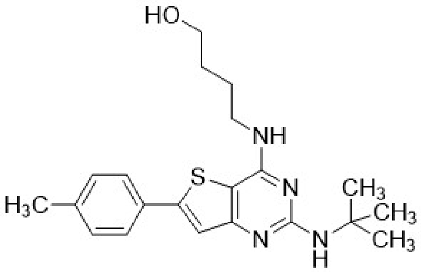

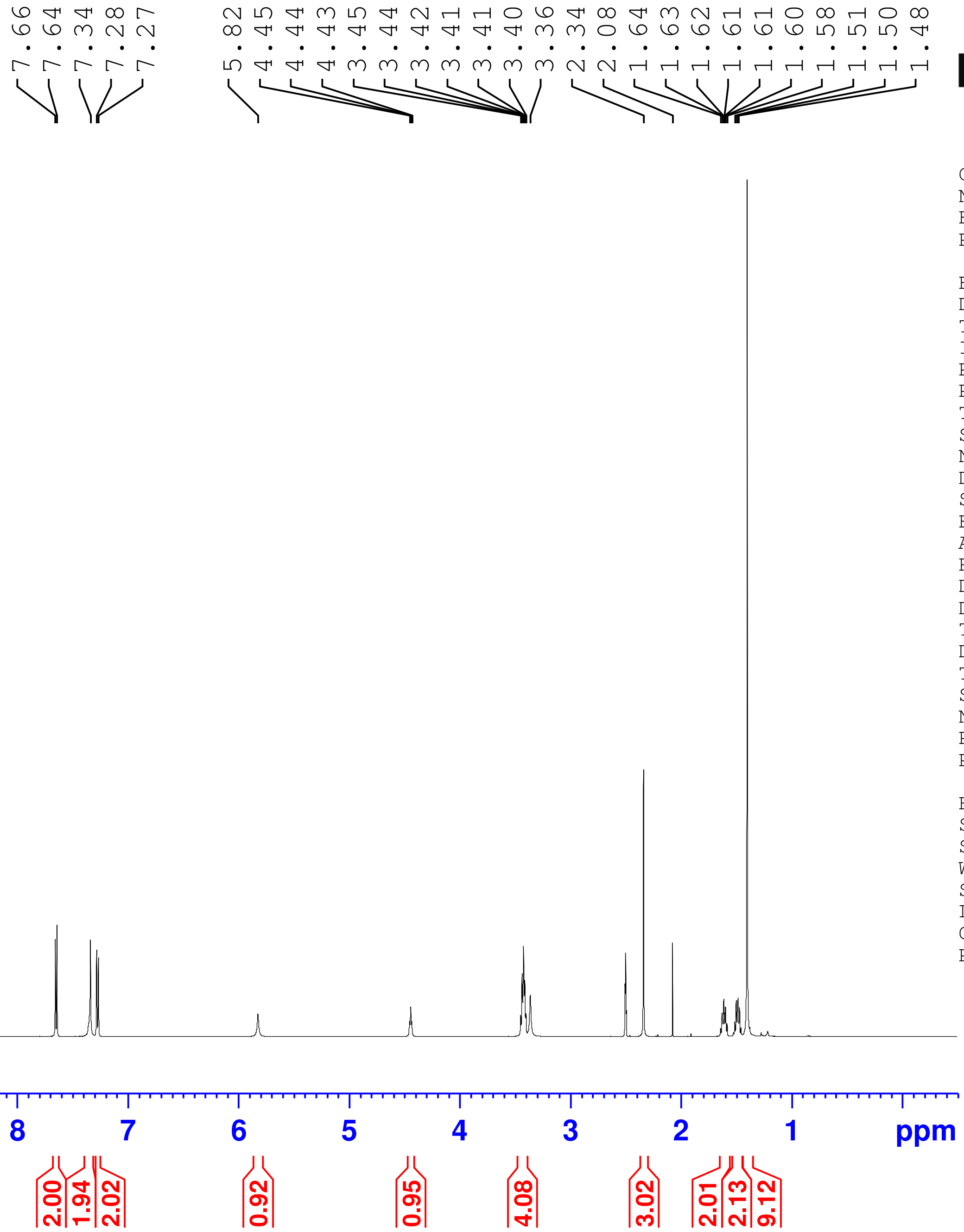

**Current Data Parameters**		
NAME	LP0113	
EXPNO	10	
PROCNO	1	
**F2 - Acquisition Parameters**		
Date_	20191010	
Time	17.38	h
INSTRUM	Spect	
PROBHD	Z136122_0002 (	
PULPROG	zg30	
TD	65536	
SOLVENT	DMSO	
NS	16	
DS	2	
SWH	10000.000	Hz
FIDRES	0.305176	Hz
AQ	3.2767999	sec
RG	10	
DW	50.000	usec
DE	10.00	usec
TE	298.0	K
D1	1.50000000	sec
TD0	1	
SFO1	500.1730885	MHz
NUC1	1H	
P1	12.90	usec
PLW1	7.00000000	W
**F2 - Processing parameters**		
SI	65536	
SF	500.1700097	MHz
WDW	EM	
SSB	0	
LB	0.10	Hz
GB	0	
PC	2.00	

LP0113 / DMSO

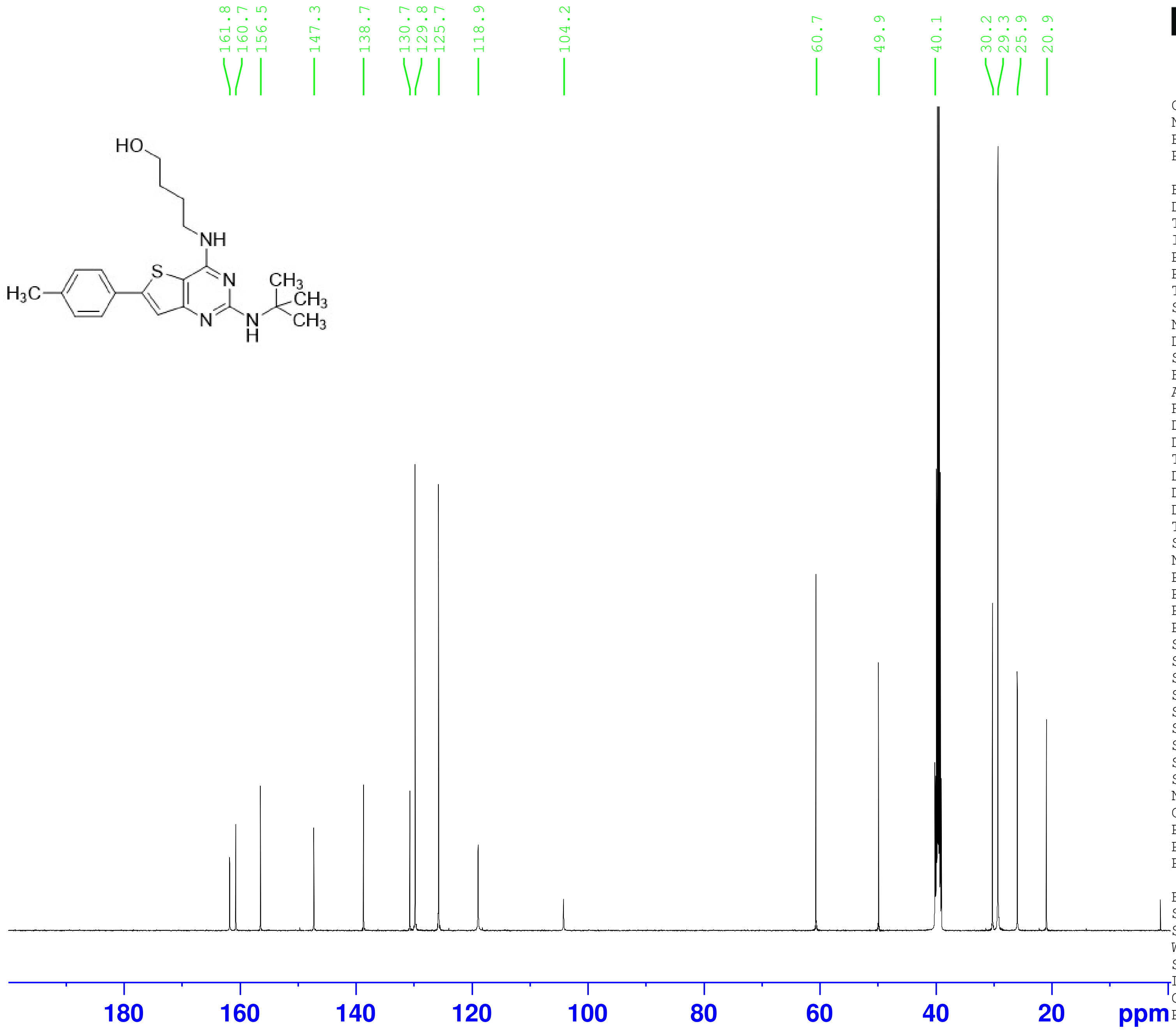

BRUKER

Current Data Parameters

NAME	LP0113
EXPNO	12
PROCNO	1

F2 - Acquisition Parameters

Date_	20191010
Time	20.01 h
INSTRUM	Spect
PROBHD	Z136122_0002 (
PULPROG	udeft
TD	20586
SOLVENT	DMSO
NS	2048
DS	0
SWH	28846.154 Hz
FIDRES	2.802502 Hz
AQ	0.3568240 sec
RG	645
DW	17.333 usec
DE	18.00 usec
TE	298.0 K
D1	3.00000000 sec
D12	0.00002000 sec
D20	200.00000000 sec
TD0	1
SFO1	125.7810526 MHz
NUC1	13C
P1	10.00 usec
P13	2000.00 usec
P26	500.00 usec
PLW1	26.00000000 W
SPNAM[5]	Crp60comp.4
SPOAL5	0.500
SPOFFS5	0 Hz
SPW5	3.97250009 W
SPNAM[8]	Crp60,0.5,20.1
SPOAL8	0.500
SPOFFS8	0 Hz
SPW8	3.97250009 W
SFO2	500.1720007 MHz
NUC2	1H
CPDPRG[2	waltz16
PCPD2	80.00 usec
PLW2	7.00000000 W
PLW12	0.18200999 W

F2 - Processing parameters

SI	32768
SF	125.7679006 MHz
WDW	EM
SSB	0
LB	2.00 Hz
GB	0
PC	2.00

LP0115 / DMSO

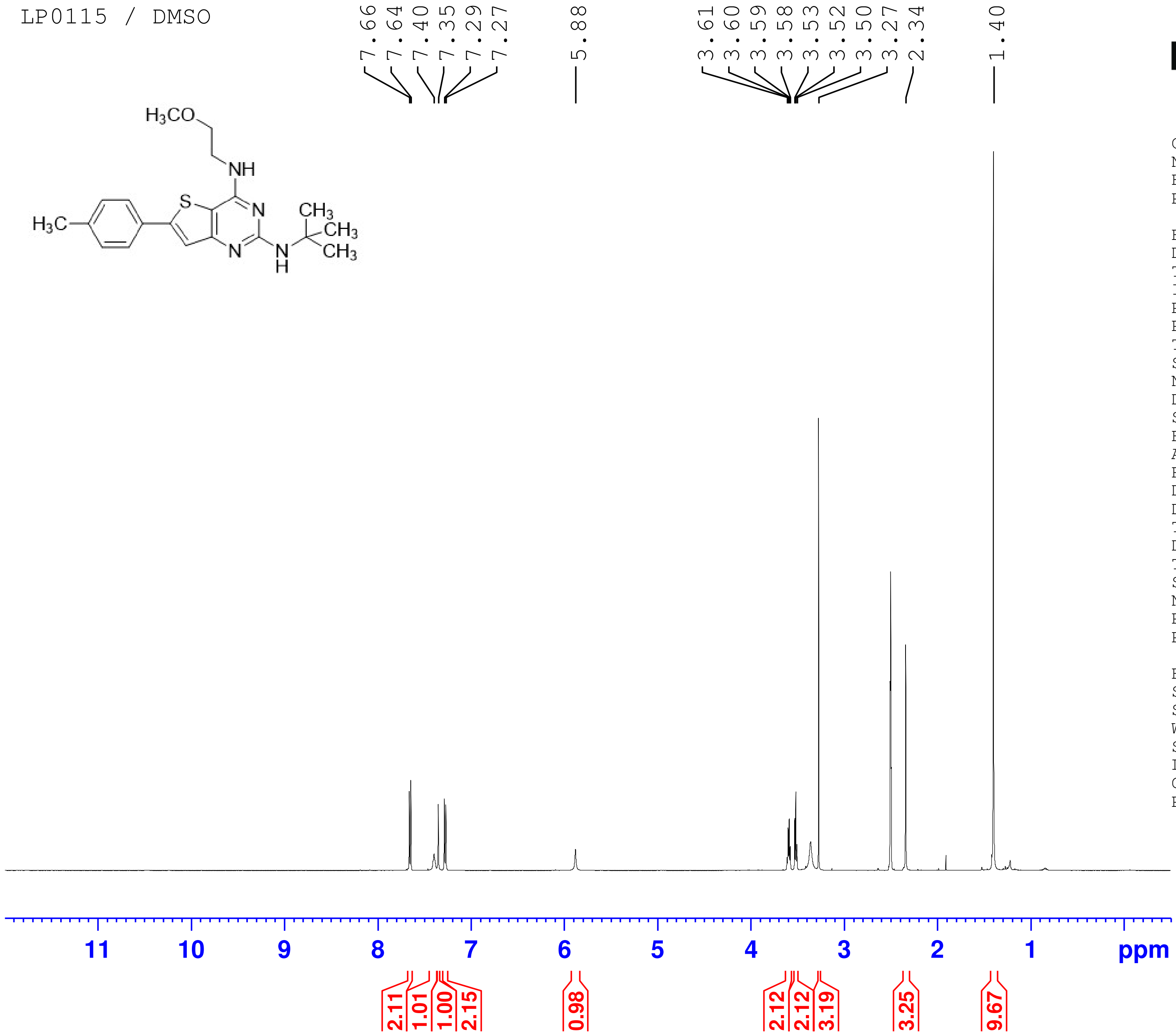

Current Data Parameters

NAME	LP0115
EXPNO	10
PROCNO	1

F2 - Acquisition Parameters

Date_	20191025
Time	23.27 h
INSTRUM	Spect
PROBHD	Z136122_0002 (
PULPROG	zg30
TD	65536
SOLVENT	DMSO
NS	16
DS	2
SWH	10000.000 Hz
FIDRES	0.305176 Hz
AQ	3.2767999 sec
RG	10
DW	50.000 usec
DE	10.00 usec
TE	298.0 K
D1	1.50000000 sec
TD0	1
SFO1	500.1730885 MHz
NUC1	1H
P1	12.90 usec
PLW1	7.00000000 W

F2 - Processing parameters

SI		65536
SF		500.1700077 MHz
WDW		EM
SSB	0	
LB		0.10 Hz
GB	0	
PC		2.00

LP0115 / DMSO

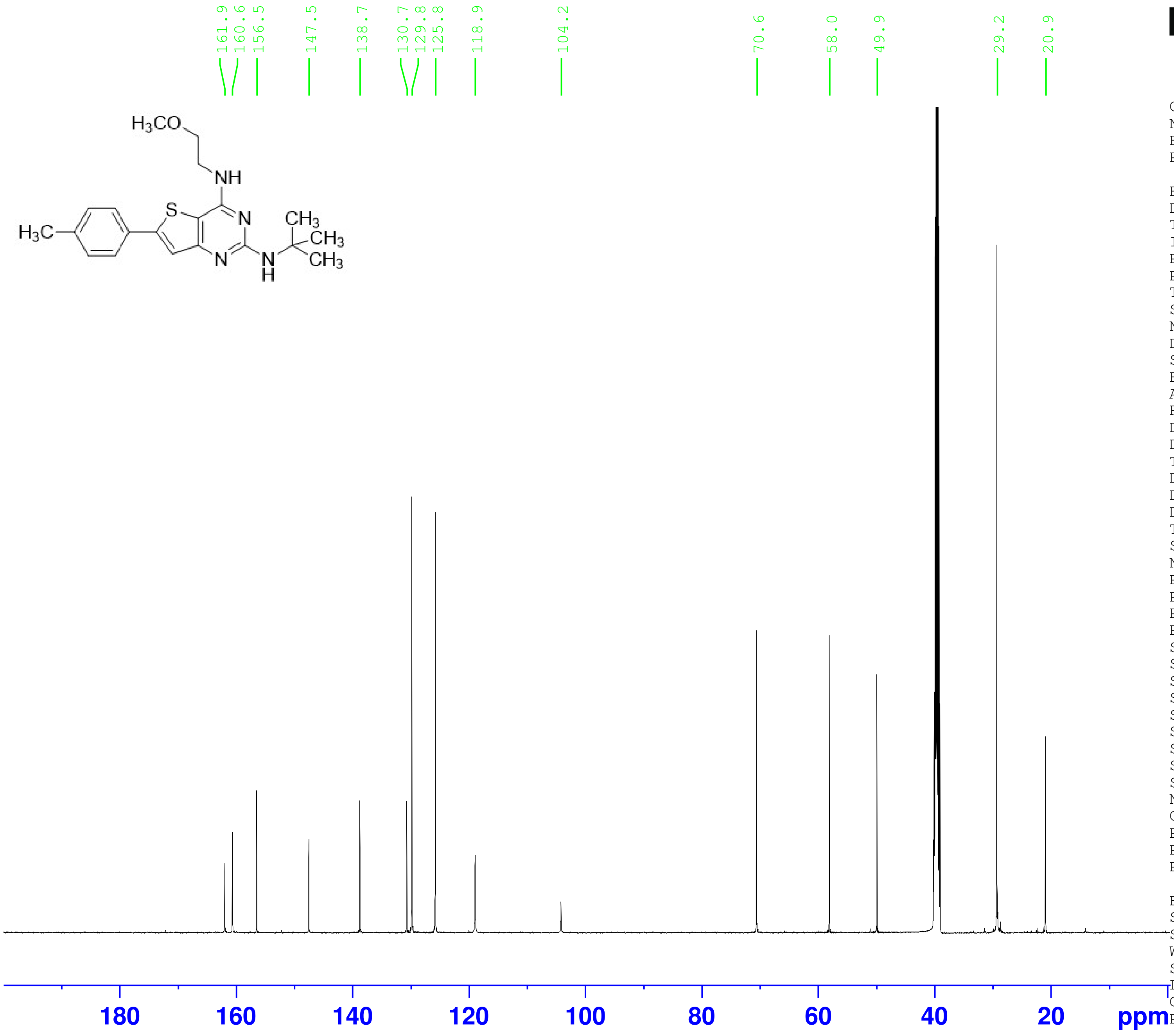

Current Data Parameters
NAME LP0115
EXPNO 11
PROCNO 1

F2 - Acquisition Parameters
Date_ 20191026
Time 3.38 h
INSTRUM Spect
PROBHD Z136122_0002 (
PULPROG udeft
TD 20586
SOLVENT DMSO
NS 3072
DS 0
SWH 28846.154 Hz
FIDRES 2.802502 Hz
AQ 0.3568240 sec
RG 406
DW 17.333 usec
DE 18.00 usec
TE 298.0 K
D1 4.00000000 sec
D12 0.00002000 sec
D20 200.00000000 sec
TD0 1
SFO1 125.7810526 MHz
NUC1 13C
P1 10.00 usec
P13 2000.00 usec
P26 500.00 usec
PLW1 26.00000000 W
SPNAM[5] Crp60comp.4
SPOAL5 0.500
SPOFFS5 0 Hz
SPW5 3.97250009 W
SPNAM[8] Crp60,0.5,20.1
SPOAL8 0.500
SPOFFS8 0 Hz
SPW8 3.97250009 W
SFO2 500.1720007 MHz
NUC2 1H
CPDPRG[2 waltz16
PCPD2 80.00 usec
PLW2 7.00000000 W
PLW12 0.18200999 W

F2 - Processing parameters
SI 32768
SF 125.7679006 MHz
WDW EM
SSB 0
LB 2.00 Hz
GB 0
PC 2.00

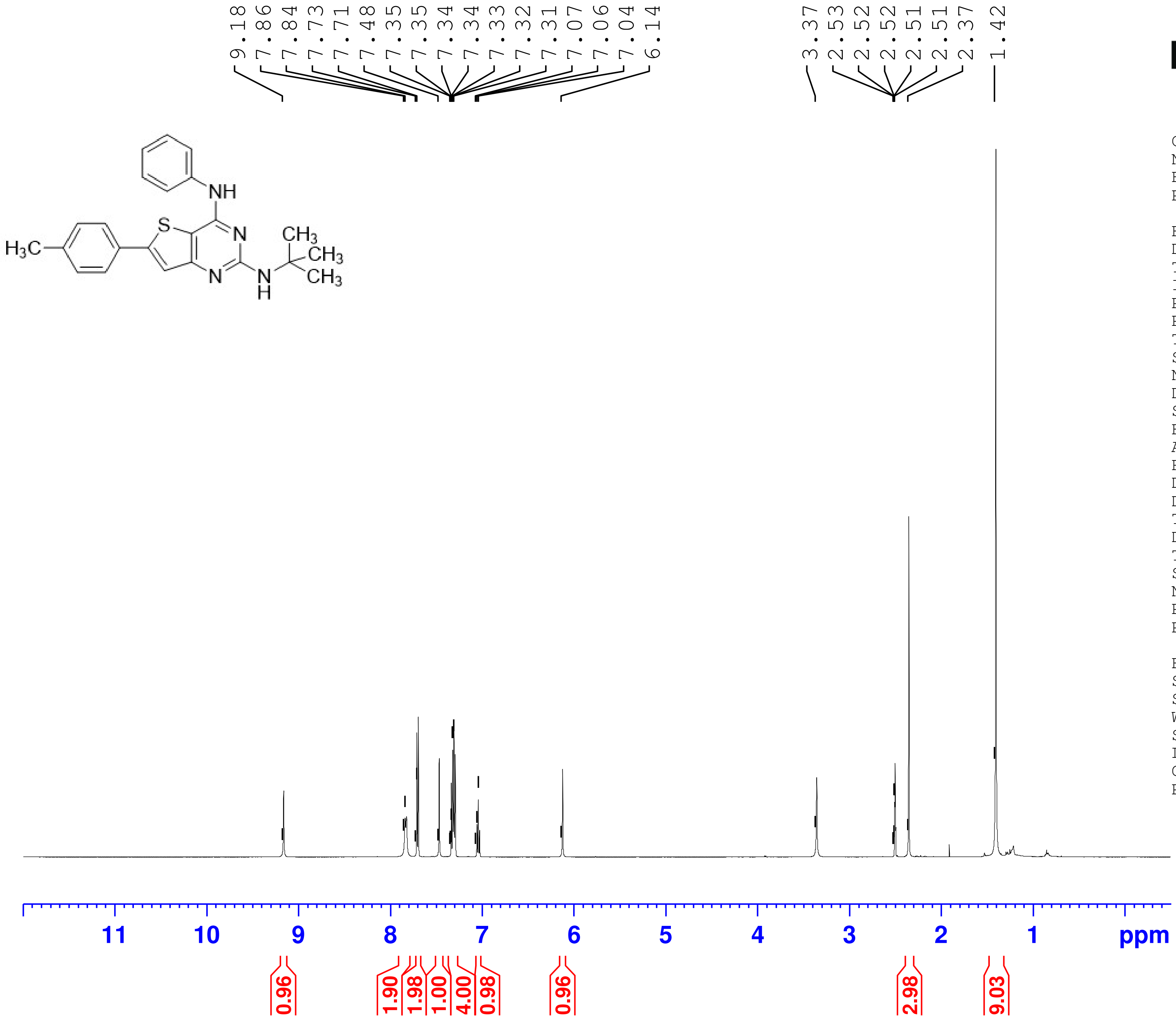

BRUKER

Current Data Parameters	
NAME	LP0176
EXPNO	10
PROCNO	1

F2 - Acquisition Parameters	
Date_	20200623
Time	14.27 h
INSTRUM	Spect
PROBHD	Z136122_0002 (
PULPROG	zg30
TD	65536
SOLVENT	DMSO
NS	16
DS	2
SWH	10000.000 Hz
FIDRES	0.305176 Hz
AQ	3.2767999 sec
RG	20.2
DW	50.000 usec
DE	10.00 usec
TE	298.0 K
D1	1.50000000 sec
TD0	1
SFO1	500.1730885 MHz
NUC1	1H
P1	12.90 usec
PLW1	7.00000000 W

F2 - Processing parameters	
SI	65536
SF	500.1700091 MHz
WDW	EM
SSB	0
LB	0.10 Hz
GB	0
PC	2.00

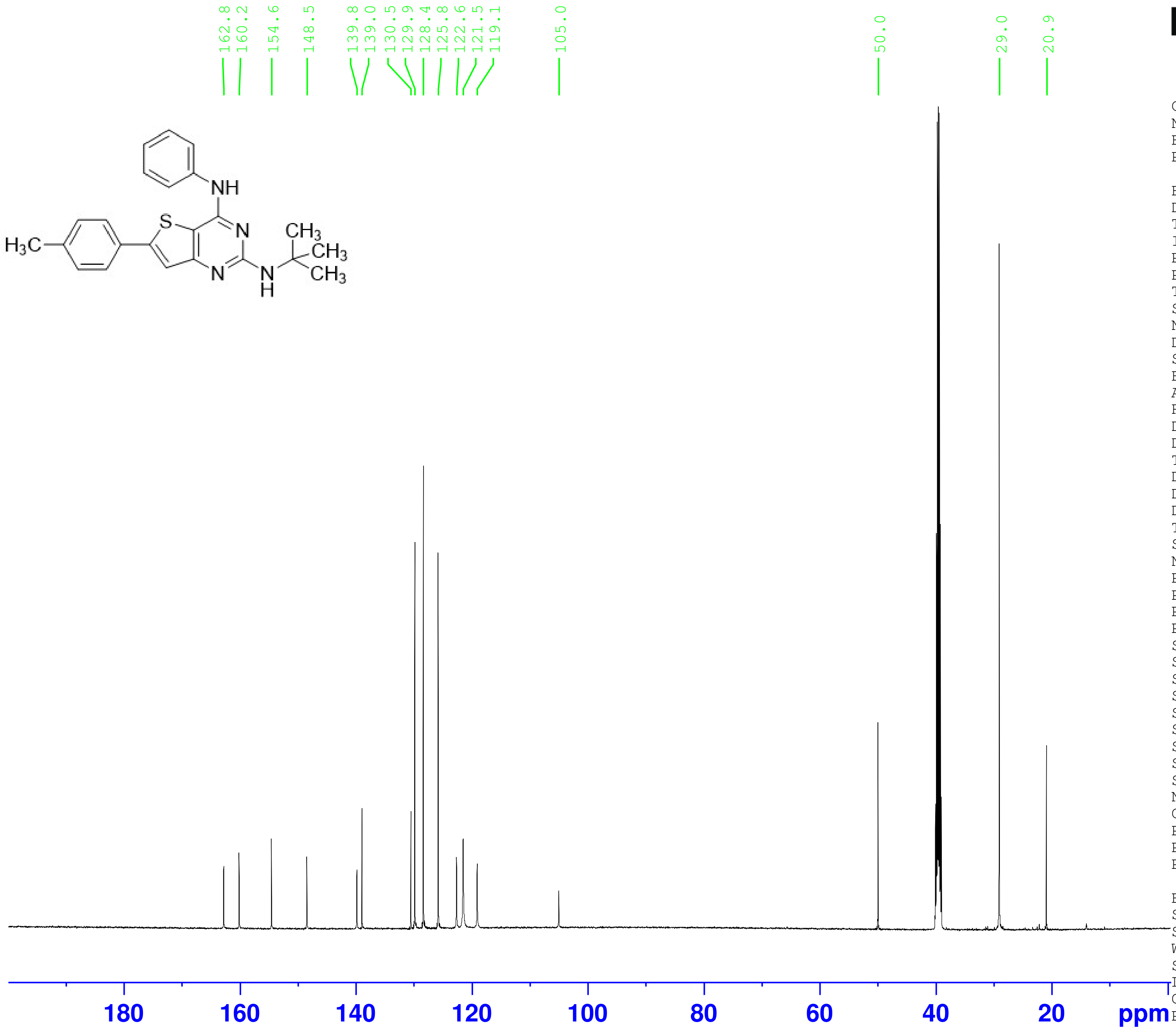

Current Data Parameters

NAME	LP0176
EXPNO	11
PROCNO	1

F2 - Acquisition Parameters

Date_	20200623
Time	15.17 h
INSTRUM	Spect
PROBHD	Z136122_0002 (
PULPROG	udeft
TD	20586
SOLVENT	DMSO
NS	653
DS	0
SWH	28846.154 Hz
FIDRES	2.802502 Hz
AQ	0.3568240 sec
RG	912
DW	17.333 usec
DE	18.00 usec
TE	298.0 K
D1	3.00000000 sec
D12	0.00002000 sec
D20	200.00000000 sec
TD0	1
SFO1	125.7810526 MHz
NUC1	13C
P1	10.00 usec
P13	2000.00 usec
P26	500.00 usec
PLW1	26.00000000 W
SPNAM[5]	Crp60comp.4
SPOAL5	0.500
SPOFFS5	0 Hz
SPW5	3.97250009 W
SPNAM[8]	Crp60,0.5,20.1
SPOAL8	0.500
SPOFFS8	0 Hz
SPW8	3.97250009 W
SFO2	500.1720007 MHz
NUC2	1H
CPDPRG[2	waltz16
PCPD2	80.00 usec
PLW2	7.00000000 W
PLW12	0.18200999 W

F2 - Processing parameters

SI	32768
SF	125.7679024 MHz
WDW	EM
SSB	0
LB	2.00 Hz
GB	0
PC	2.00

LP0083 / dmso
25/10/2019
TE 373K

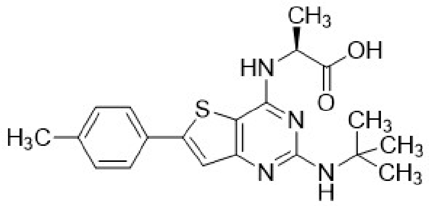

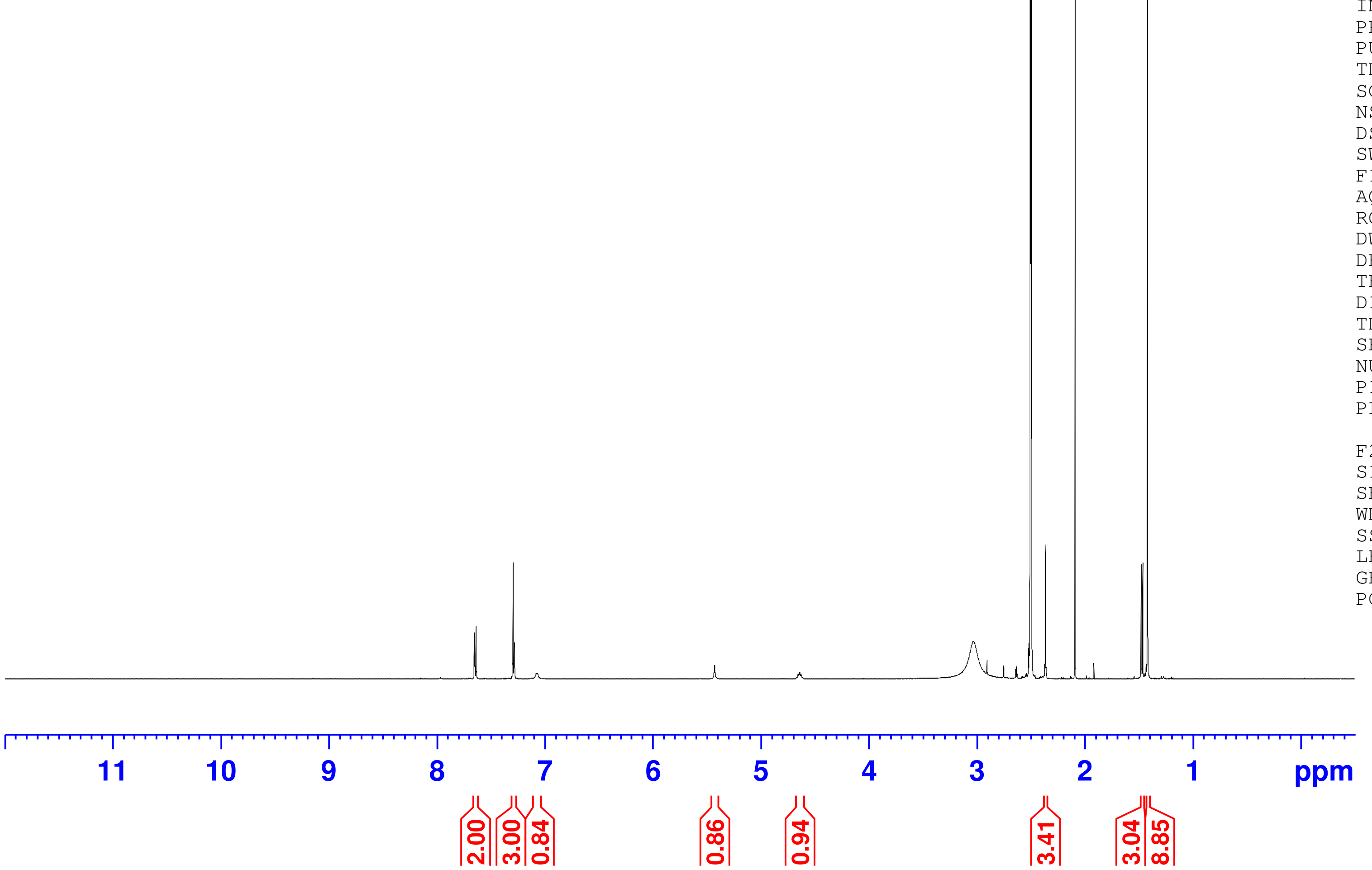

Current Data Parameters

NAME	LP0083-2
EXPNO	1
PROCNO	1

F2 - Acquisition Parameters

Date_	20191025
Time	10.25 h
INSTRUM	Spect
PROBHD	Z136122_0002 (
PULPROG	zg30
TD	65536
SOLVENT	DMSO
NS	32
DS	2
SWH	8012.820 Hz
FIDRES	0.244532 Hz
AQ	4.0894465 sec
RG	10
DW	62.400 usec
DE	10.00 usec
TE	373.0 K
D1	1.50000000 sec
TD0	1
SFO1	500.1735012 MHz
NUC1	1H
P1	12.90 usec
PLW1	7.00000000 W

F2 - Processing parameters

SI	65536
SF	500.1700085 MHz
WDW	EM
SSB	0
LB	0.10 Hz
GB	0
PC	1.00

LP0083 -2/ dmso
TE 373K

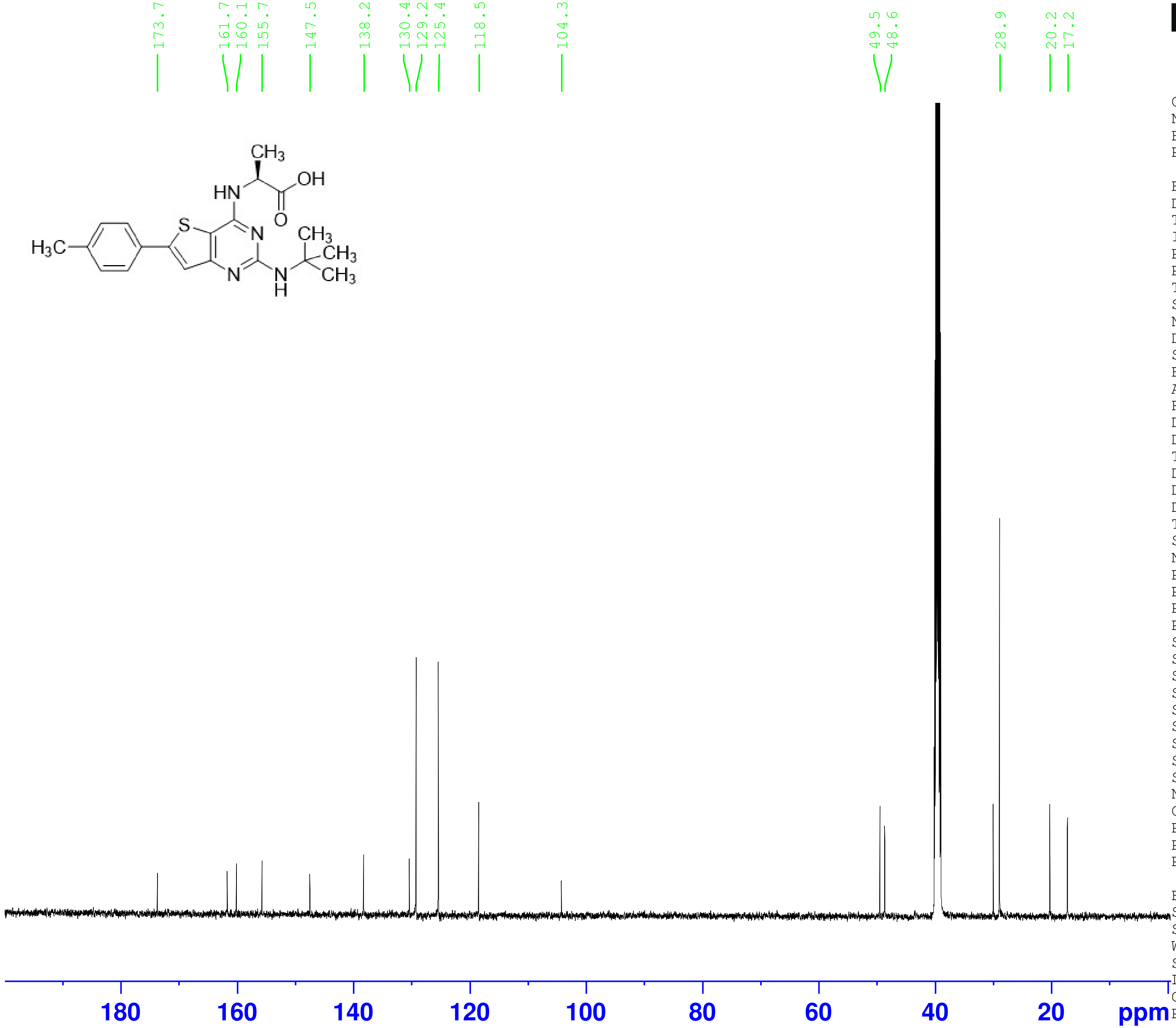

Current Data Parameters

NAME	LP0083-2
EXPNO	2
PROCNO	1

F2 - Acquisition Parameters

Date_	20191025
Time	12.06 h
INSTRUM	Spect
PROBHD	Z136122_0002 (
PULPROG	udeft
TD	20586
SOLVENT	DMSO
NS	1232
DS	0
SWH	28846.154 Hz
FIDRES	2.802502 Hz
AQ	0.3568240 sec
RG	575
DW	17.333 usec
DE	18.00 usec
TE	373.0 K
D1	4.00000000 sec
D12	0.00002000 sec
D20	200.00000000 sec
TD0	1
SFO1	125.7810526 MHz
NUC1	13C
P1	10.00 usec
P13	2000.00 usec
P26	500.00 usec
PLW1	26.00000000 W
SPNAM[5]	Crp60comp.4
SPOAL5	0.500
SPOFFS5	0 Hz
SPW5	3.97250009 W
SPNAM[8]	Crp60,0.5,20.1
SPOAL8	0.500
SPOFFS8	0 Hz
SPW8	3.97250009 W
SFO2	500.1720007 MHz
NUC2	1H
CPDPRG[2	waltz16
PCPD2	80.00 usec
PLW2	7.00000000 W
PLW12	0.18200999 W

F2 - Processing parameters

SI	32768
SF	125.7679649 MHz
WDW	EM
SSB	0
LB	2.00 Hz
GB	0
PC	1.40

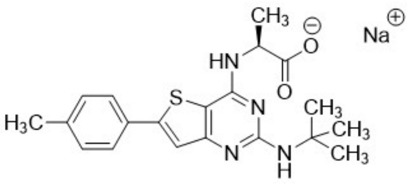

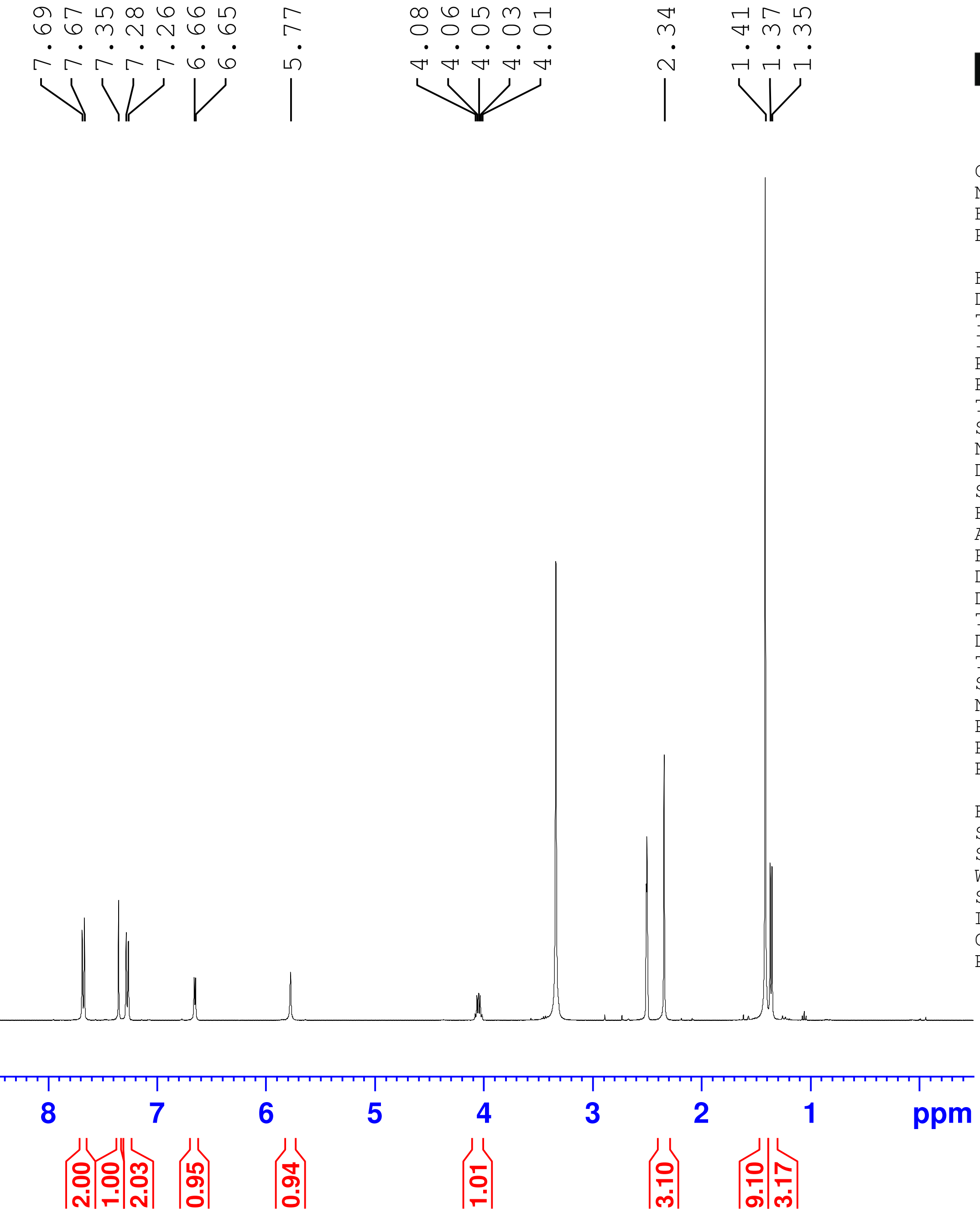

Current Data Parameters

NAME	LAG-202-LP0190
EXPNO	20
PROCNO	1

F2 - Acquisition Parameters

Date_	20220314
Time	9.40 h
INSTRUM	spect
PROBHD	Z104450_0260 (
PULPROG	zg30
TD	65536
SOLVENT	DMSO
NS	16
DS	2
SWH	8012.820 Hz
FIDRES	0.244532 Hz
AQ	4.0894465 sec
RG	174.69
DW	62.400 usec
DE	6.50 usec
TE	298.0 K
D1	1.50000000 sec
TD0	1
SFO1	400.1524710 MHz
NUC1	1H
P0	5.00 usec
P1	15.00 usec
PLW1	7.59549999 W

F2 - Processing parameters

SI	65536
SF	400.1500071 MHz
WDW	EM
SSB	0
LB	0.10 Hz
GB	0
PC	4.00

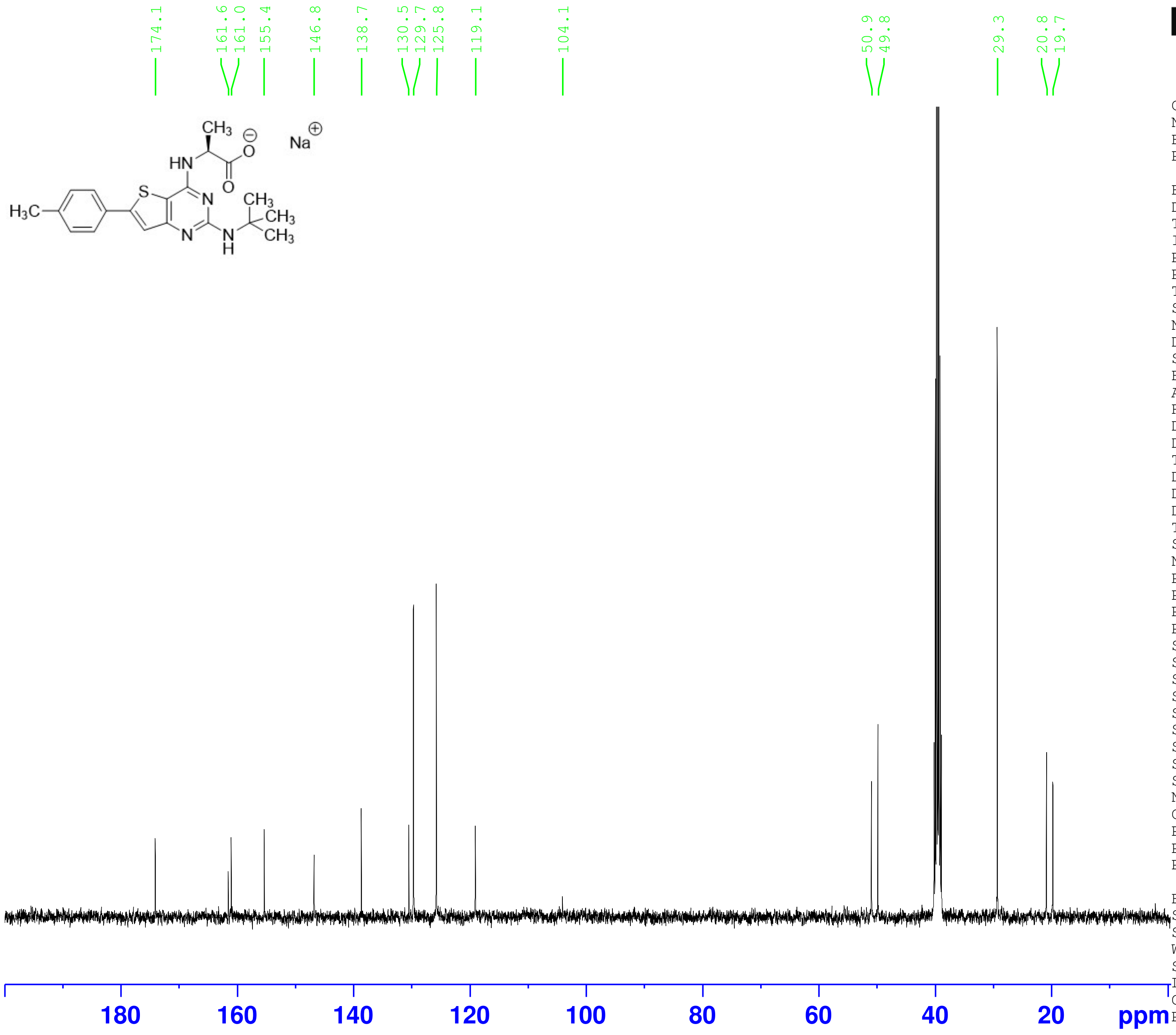

Current Data Parameters
NAME LAG-202-LP0190
EXPNO 25
PROCNO 1

F2 - Acquisition Parameters

Date_	20220314	
Time	13.41	h
INSTRUM	spect	
PROBHD	Z104450_0260 (	
PULPROG	udeft	
TD	17304	
SOLVENT	DMSO	
NS	2516	
DS	0	
SWH	24038.461	Hz
FIDRES	2.778370	Hz
AQ	0.3599232	sec
RG	196.32	
DW	20.800	usec
DE	6.50	usec
TE	298.0	K
D1	3.00000000	sec
D12	0.00002000	sec
D20	200.00000000	sec
TD0	1	
SFO1	100.6278588	MHz
NUC1	13C	
P1	10.00	usec
P13	2000.00	usec
P26	500.00	usec
PLW1	51.71699905	W
SPNAM[5]	Crp60comp.4	
SPOAL5	0.500	
SPOFFS5	0	Hz
SPW5	7.90180016	W
SPNAM[8]	Crp60,0.5,20.1	
SPOAL8	0.500	
SPOFFS8	0	Hz
SPW8	7.90180016	W
SFO2	400.1516006	MHz
NUC2	1H	
CPDPRG[2	waltz16	
PCPD2	70.00	usec
PLW2	7.59549999	W
PLW12	0.34495080	W

F2 - Processing parameters

SI	32768	
SF	100.6178471	MHz
WDW	EM	
SSB	0	
LB	2.00	Hz
GB	0	
PC	1.40	

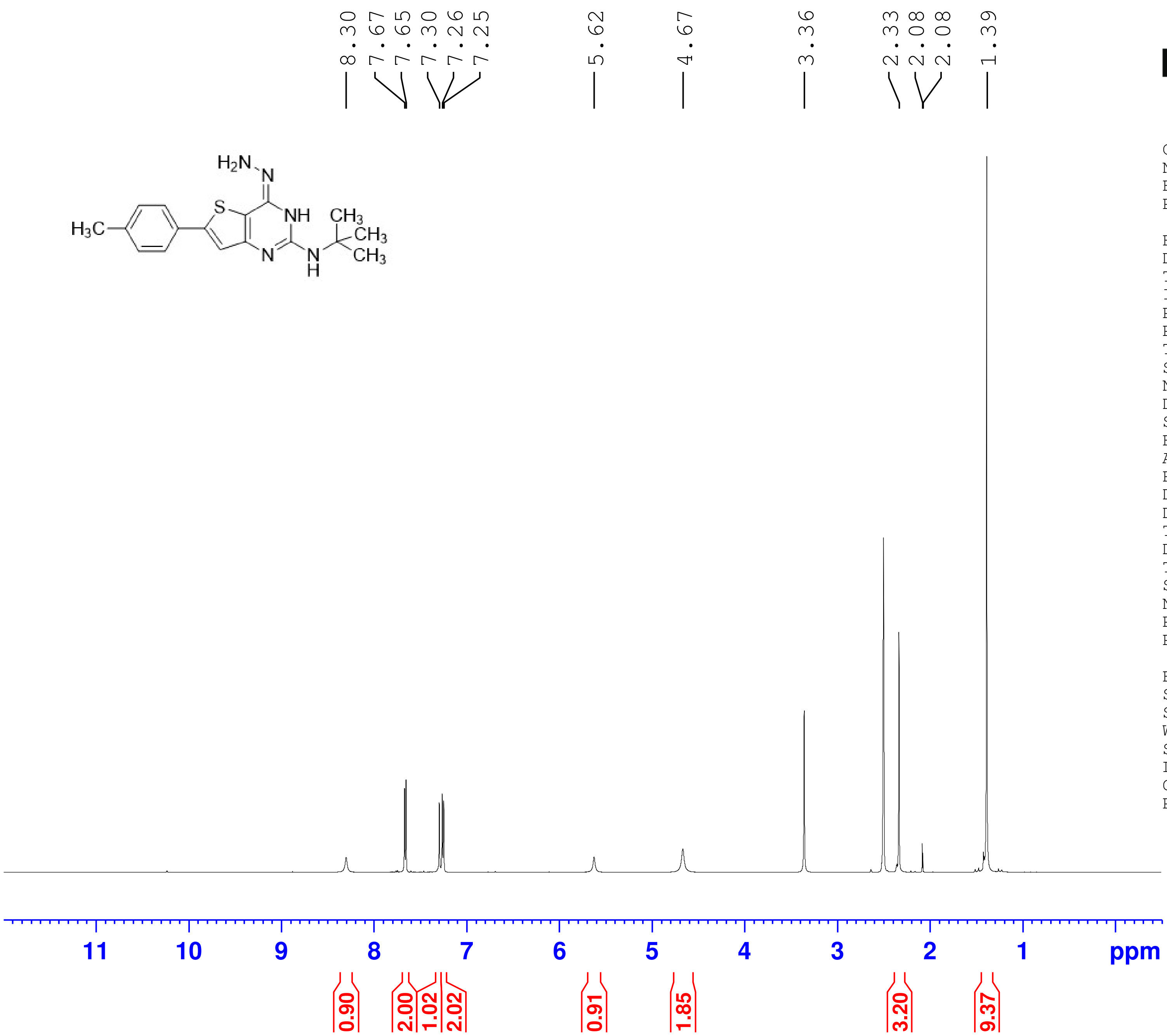

BRUKER

Current Data Parameters
NAME LP0080
EXPNO 10
PROCNO 1

F2 - Acquisition Parameters

Date_	20191025	
Time	17.38	h
INSTRUM	Spect	
PROBHD	Z136122_0002 (	
PULPROG	zg30	
TD	65536	
SOLVENT	DMSO	
NS	16	
DS	2	
SWH	10000.000	Hz
FIDRES	0.305176	Hz
AQ	3.2767999	sec
RG	10	
DW	50.000	usec
DE	10.00	usec
TE	298.0	K
D1	1.50000000	sec
TD0	1	
SFO1	500.1730885	MHz
NUC1	1H	
P1	12.90	usec
PLW1	7.00000000	W

F2 - Processing parameters

SI	65536	
SF	500.1700072	MHz
WDW	EM	
SSB	0	
LB	0.10	Hz
GB	0	
PC	2.00	

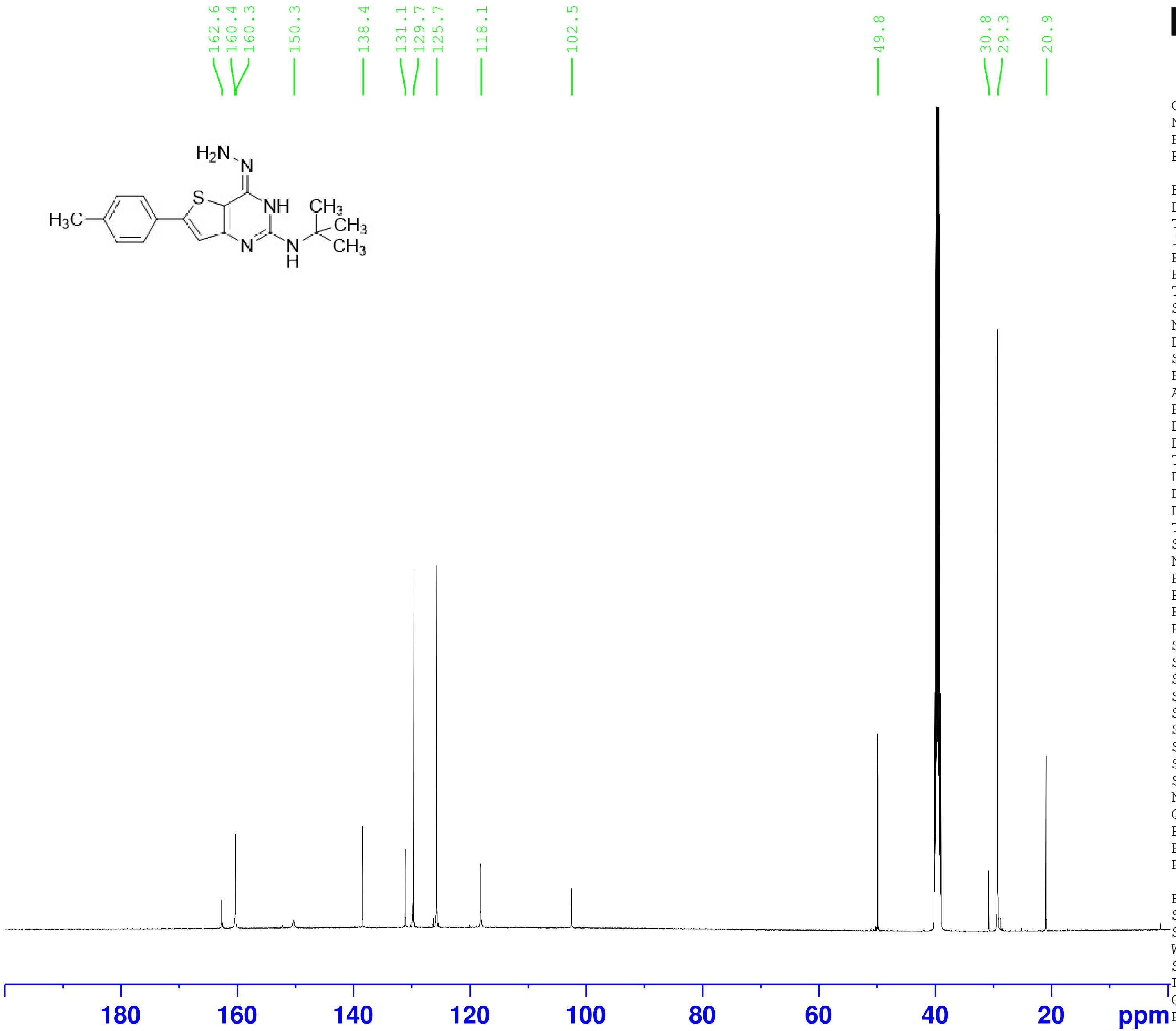

Current Data Parameters

NAME	LP0080
EXPNO	11
PROCNO	1

F2 - Acquisition Parameters

Date_	20191025
Time	23.10 h
INSTRUM	Spect
PROBHD	Z136122_0002 (
PULPROG	udeft
TD	20586
SOLVENT	DMSO
NS	4096
DS	0
SWH	28846.154 Hz
FIDRES	2.802502 Hz
AQ	0.3568240 sec
RG	912
DW	17.333 usec
DE	18.00 usec
TE	298.0 K
D1	4.00000000 sec
D12	0.00002000 sec
D20	200.00000000 sec
TD0	1
SFO1	125.7810526 MHz
NUC1	13C
P1	10.00 usec
P13	2000.00 usec
P26	500.00 usec
PLW1	26.00000000 W
SPNAM[5]	Crp60comp.4
SPOAL5	0.500
SPOFFS5	0 Hz
SPW5	3.97250009 W
SPNAM[8]	Crp60,0.5,20.1
SPOAL8	0.500
SPOFFS8	0 Hz
SPW8	3.97250009 W
SFO2	500.1720007 MHz
NUC2	1H
CPDPRG[2	waltz16
PCPD2	80.00 usec
PLW2	7.00000000 W
PLW12	0.18200999 W

F2 - Processing parameters

SI	32768
SF	125.7679001 MHz
WDW	EM
SSB	0
LB	2.00 Hz
GB	0
PC	2.00

LP0211-pur

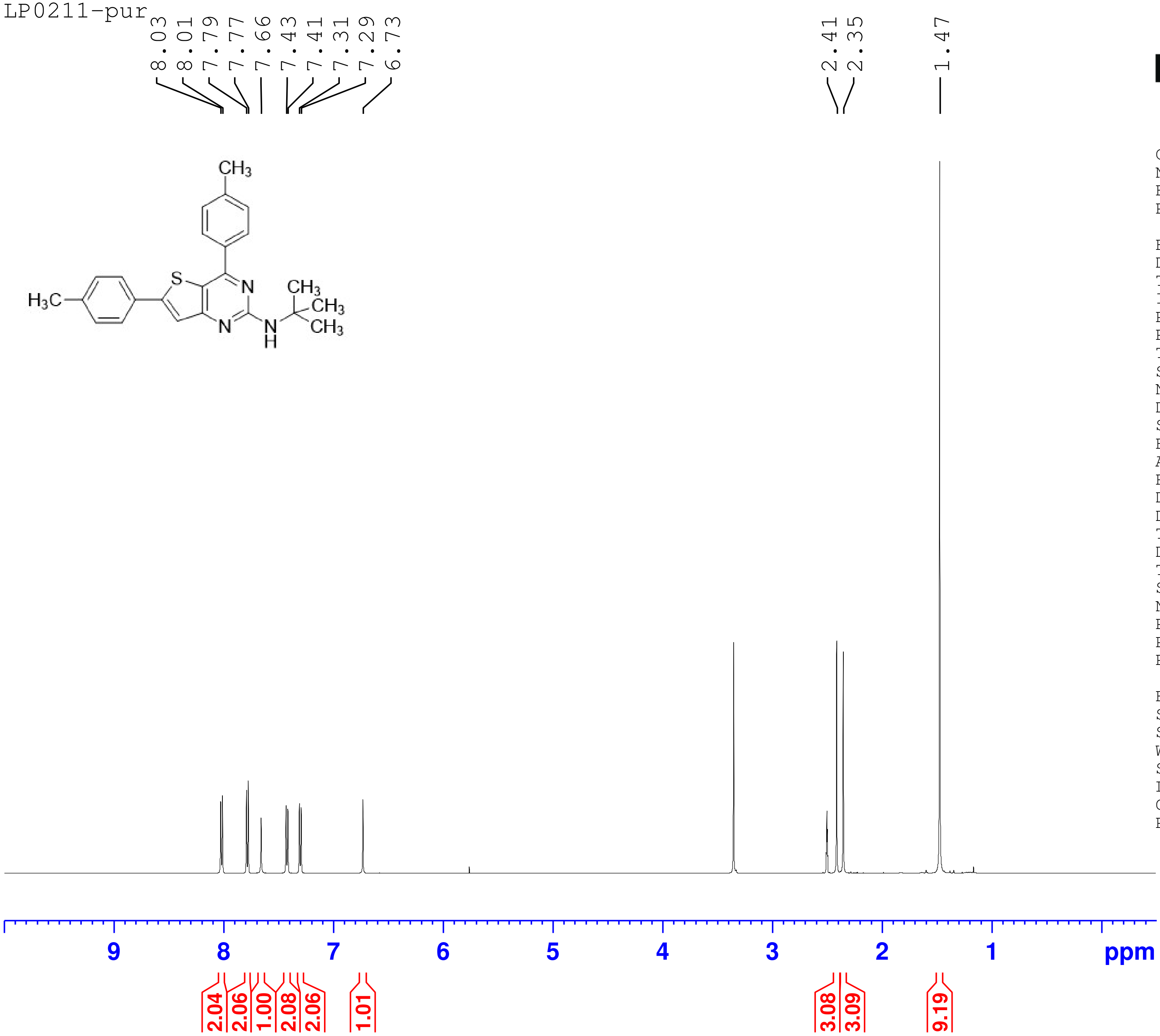

BRUKER

Current Data Parameters	
NAME	LAG-123
EXPNO	50
PROCNO	1
F2 - Acquisition Parameters	
Date_	20210617
Time	12.30 h
INSTRUM	Spect
PROBHD	Z136122_0002 (
PULPROG	zg30
TD	65536
SOLVENT	DMSO
NS	16
DS	2
SWH	10000.000 Hz
FIDRES	0.305176 Hz
AQ	3.2767999 sec
RG	10
DW	50.000 usec
DE	10.00 usec
TE	298.0 K
D1	1.50000000 sec
TD0	1
SFO1	500.1730885 MHz
NUC1	1H
P0	4.30 usec
P1	12.90 usec
PLW1	7.00000000 W
F2 - Processing parameters	
SI	65536
SF	500.1700061 MHz
WDW	EM
SSB	0
LB	0.10 Hz
GB	0
PC	2.00

LP0211-pur

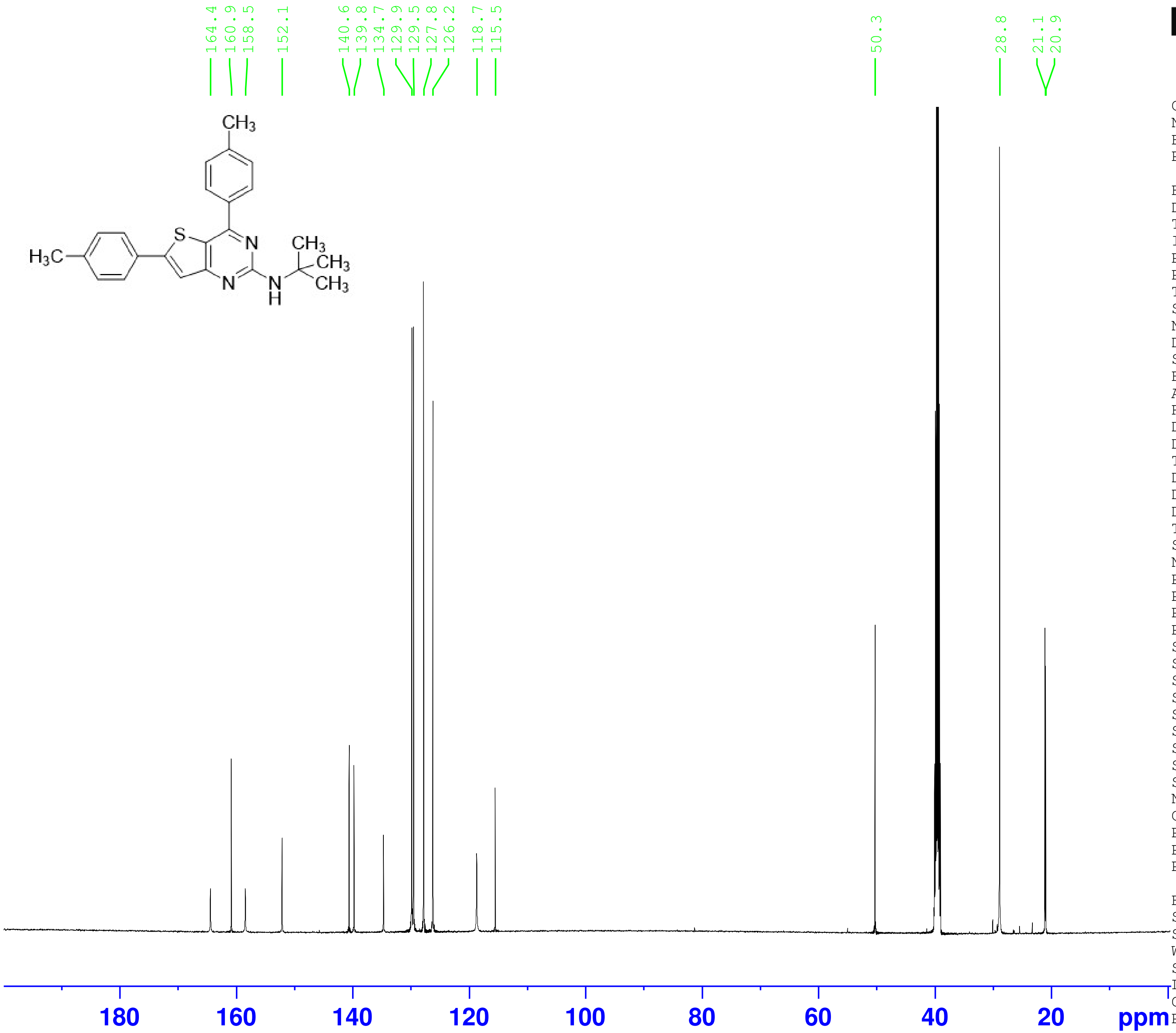

BRUKER

Current Data Parameters
NAME LAG-123
EXPNO 51
PROCNO 1

F2 - Acquisition Parameters
Date_ 20210617
Time 14.29 h
INSTRUM Spect
PROBHD Z136122_0002 (
PULPROG udeft
TD 16384
SOLVENT DMSO
NS 1774
DS 0
SWH 28846.154 Hz
FIDRES 3.521259 Hz
AQ 0.2839893 sec
RG 812
DW 17.333 usec
DE 18.00 usec
TE 298.0 K
D1 3.00000000 sec
D12 0.00002000 sec
D20 200.00000000 sec
TD0 1
SFO1 125.7810526 MHz
NUC1 13C
P1 10.00 usec
P13 2000.00 usec
P26 500.00 usec
PLW1 26.00000000 W
SPNAM[5] Crp60comp.4
SPOAL5 0.500
SPOFFS5 0 Hz
SPW5 3.97250009 W
SPNAM[8] Crp60,0.5,20.1
SPOAL8 0.500
SPOFFS8 0 Hz
SPW8 3.97250009 W
SFO2 500.1720007 MHz
NUC2 1H
CPDPRG[2 waltz16
PCPD2 80.00 usec
PLW2 7.00000000 W
PLW12 0.18200999 W

F2 - Processing parameters
SI 32768
SF 125.7679024 MHz
WDW EM
SSB 0
LB 2.00 Hz
GB 0
PC 2.00

LP0228-pur

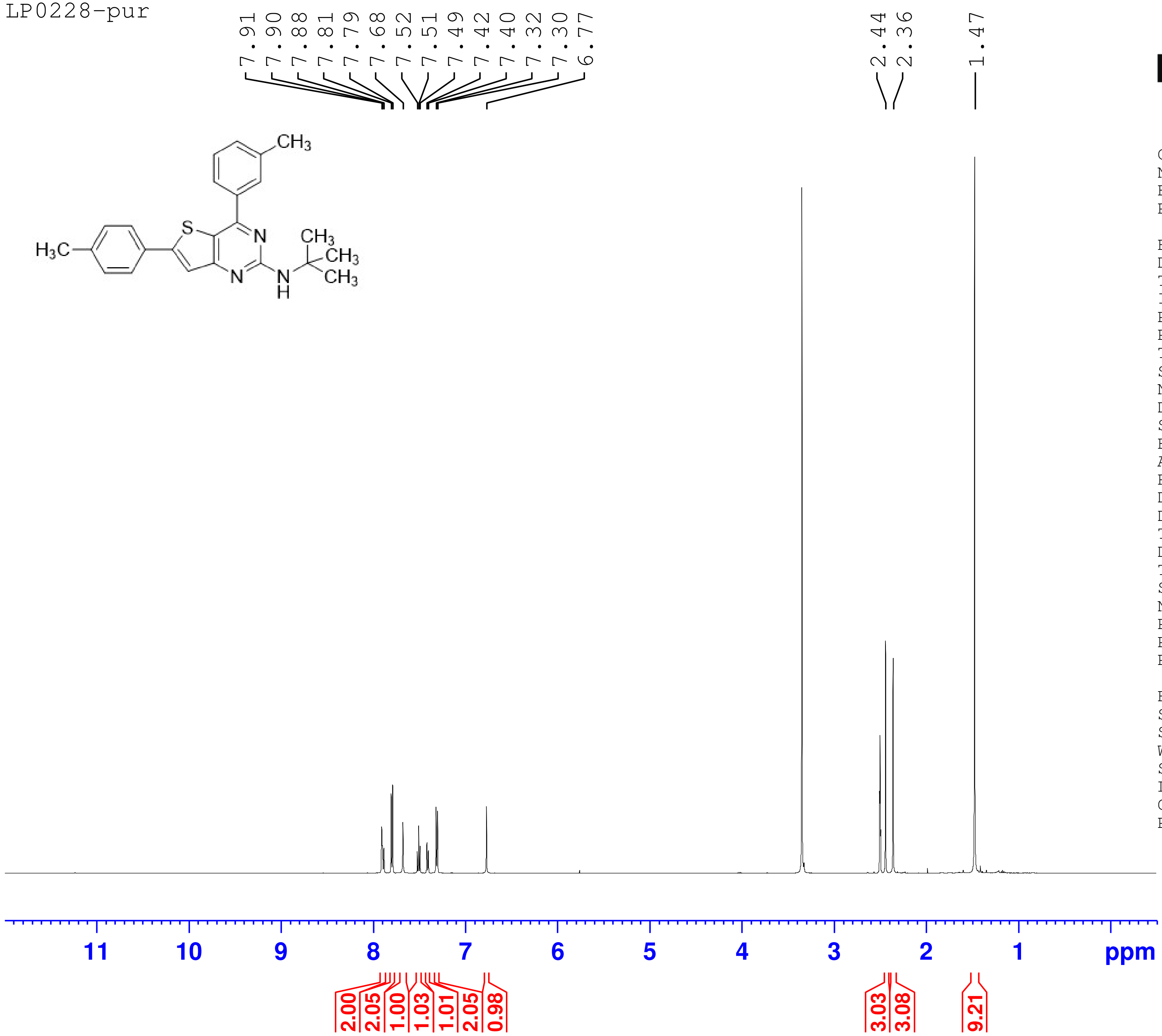

BRUKER

Current Data Parameters
NAME LAG-124-LP0228-pur
EXPNO 50
PROCNO 1

F2 - Acquisition Parameters

Date_	20210617	
Time	14.33	h
INSTRUM	Spect	
PROBHD	Z136122_0002 (	
PULPROG	zg30	
TD	65536	
SOLVENT	DMSO	
NS	16	
DS	2	
SWH	10000.000	Hz
FIDRES	0.305176	Hz
AQ	3.2767999	sec
RG	22.6	
DW	50.000	usec
DE	10.00	usec
TE	298.0	K
D1	1.50000000	sec
TD0	1	
SFO1	500.1730885	MHz
NUC1	1H	
P0	4.30	usec
P1	12.90	usec
PLW1	7.00000000	W

F2 - Processing parameters

SI	65536	
SF	500.1700061	MHz
WDW	EM	
SSB	0	
LB	0.10	Hz
GB	0	
PC	2.00	

LP0228-pur

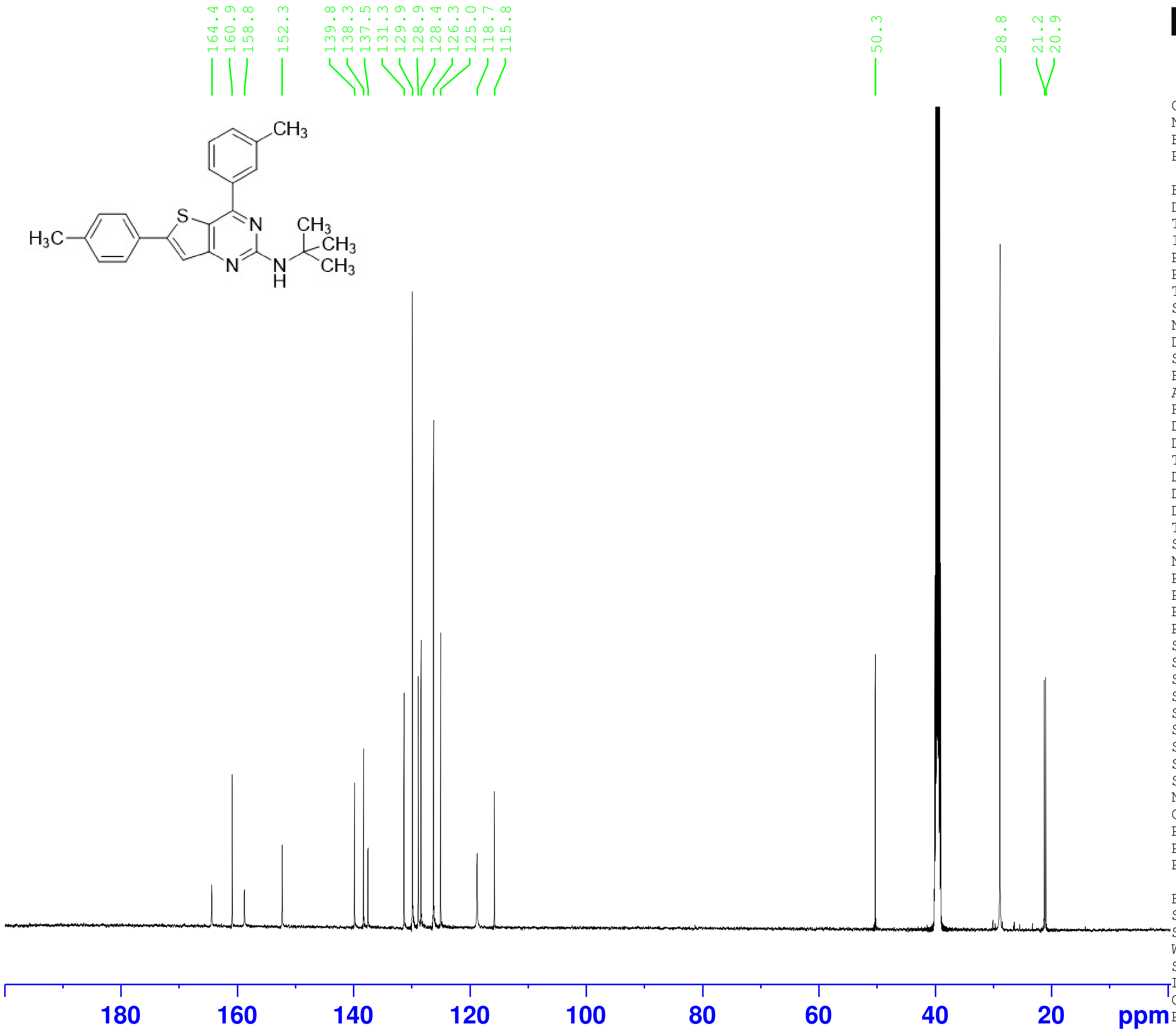

BRUKER

Current Data Parameters
NAME LAG-124
EXPNO 51
PROCNO 1

F2 - Acquisition Parameters
Date_ 20210617
Time 16.02 h
INSTRUM Spect
PROBHD Z136122_0002 (
PULPROG udeft
TD 16384
SOLVENT DMSO
NS 1289
DS 0
SWH 28846.154 Hz
FIDRES 3.521259 Hz
AQ 0.2839893 sec
RG 912
DW 17.333 usec
DE 18.00 usec
TE 298.0 K
D1 3.00000000 sec
D12 0.00002000 sec
D20 200.00000000 sec
TD0 1
SFO1 125.7810526 MHz
NUC1 13C
P1 10.00 usec
P13 2000.00 usec
P26 500.00 usec
PLW1 26.00000000 W
SPNAM[5] Crp60comp.4
SPOAL5 0.500
SPOFFS5 0 Hz
SPW5 3.97250009 W
SPNAM[8] Crp60,0.5,20.1
SPOAL8 0.500
SPOFFS8 0 Hz
SPW8 3.97250009 W
SFO2 500.1720007 MHz
NUC2 1H
CPDPRG[2 waltz16
PCPD2 80.00 usec
PLW2 7.00000000 W
PLW12 0.18200999 W

F2 - Processing parameters
SI 32768
SF 125.7679019 MHz
WDW EM
SSB 0
LB 2.00 Hz
GB 0
PC 2.00

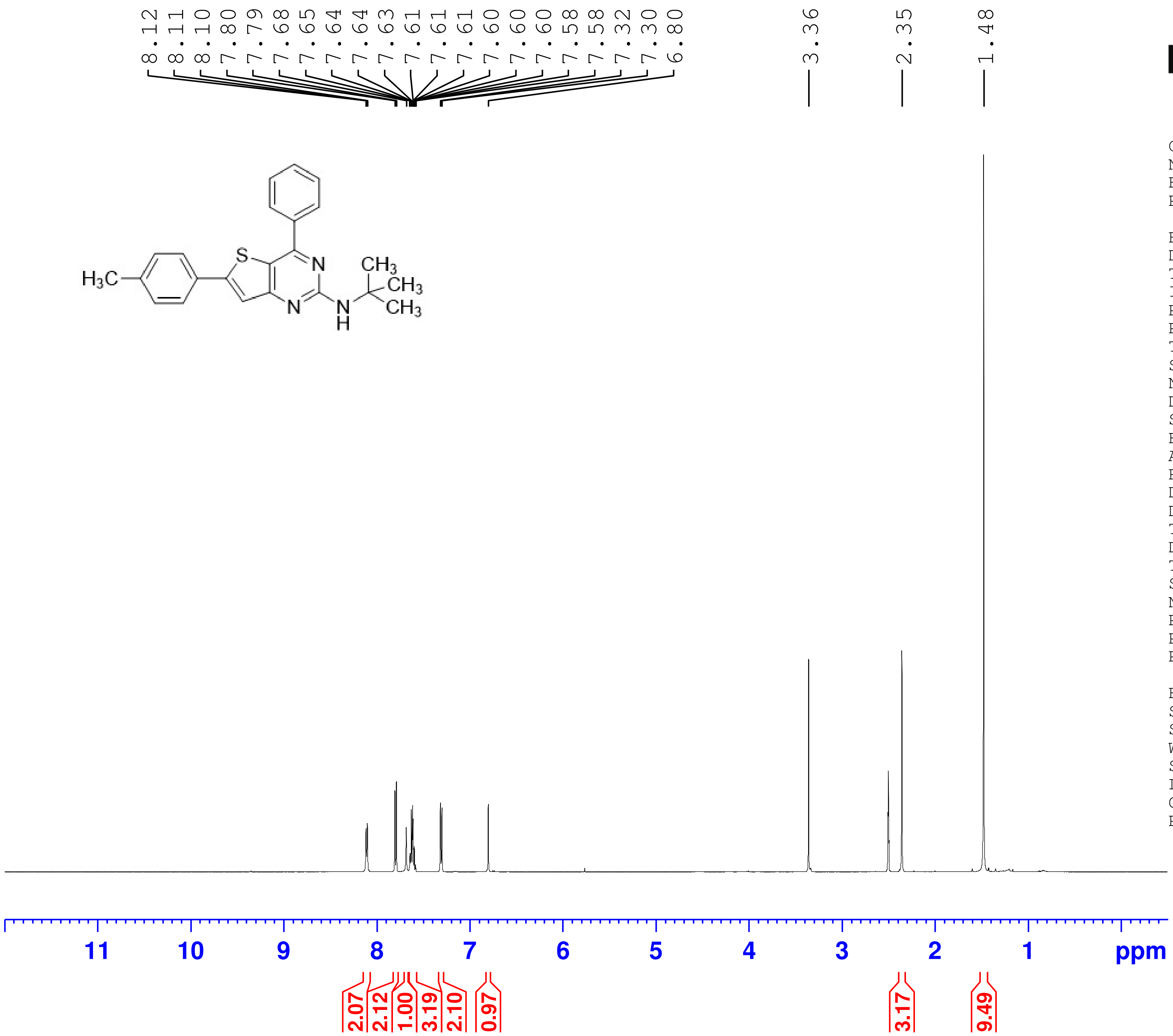

BRUKER

Current Data Parameters
NAME LAG-147-LP0220
EXPNO 50
PROCNO 1

F2 - Acquisition Parameters

Date_	20211015	
Time	14.11	h
INSTRUM	Spect	
PROBHD	Z136122_0002 (	
PULPROG	zg30	
TD	65536	
SOLVENT	DMSO	
NS	16	
DS	2	
SWH	10000.000	Hz
FIDRES	0.305176	Hz
AQ	3.2767999	sec
RG	20.2	
DW	50.000	usec
DE	10.00	usec
TE	298.0	K
D1	1.50000000	sec
TD0	1	
SFO1	500.1730885	MHz
NUC1	1H	
P0	4.30	usec
P1	12.90	usec
PLW1	7.00000000	W

F2 - Processing parameters

SI	65536	
SF	500.1700067	MHz
WDW	EM	
SSB	0	
LB	0.10	Hz
GB	0	
PC	2.00	

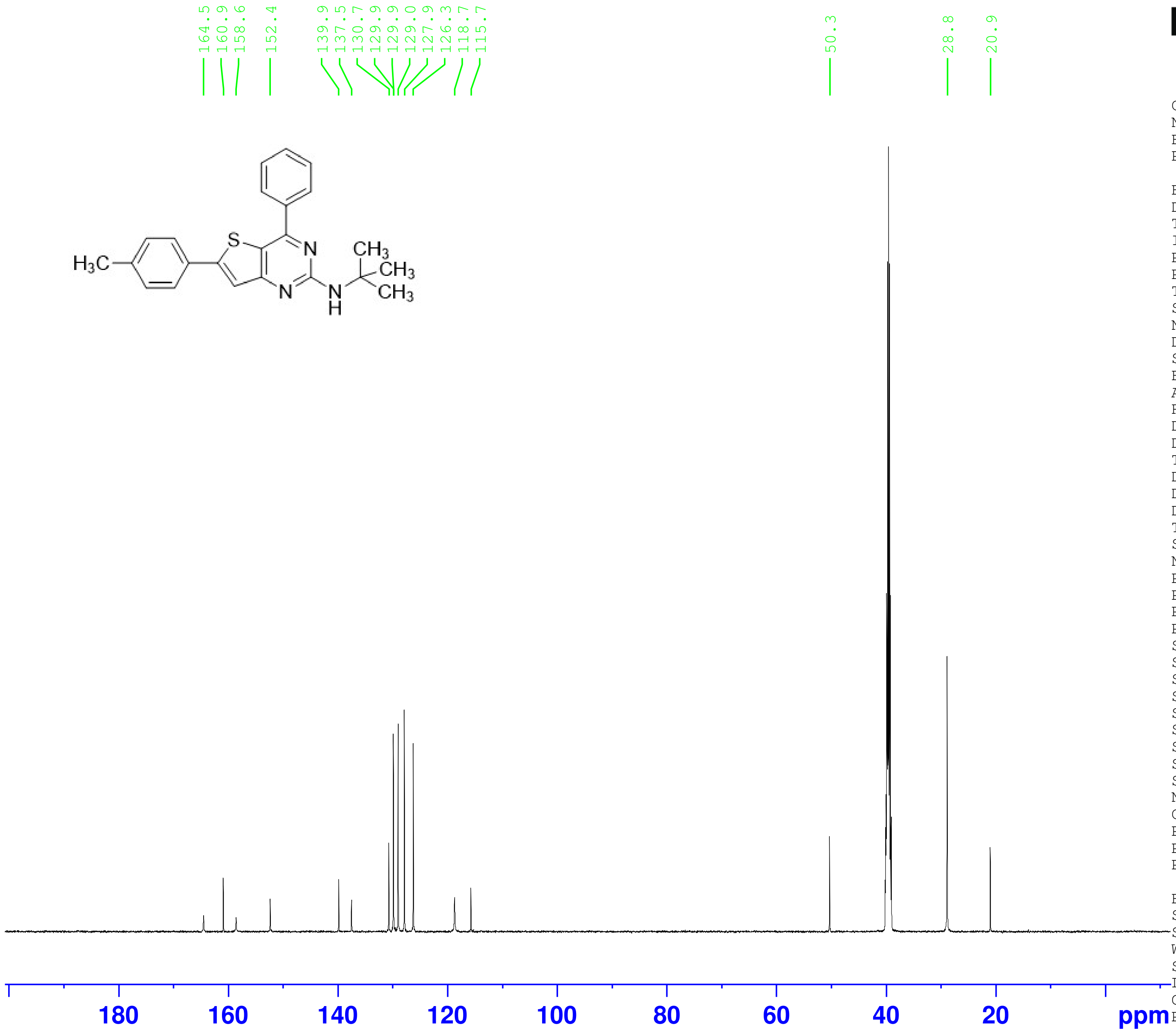

BRUKER

Current Data Parameters

NAME	LAG-8-LP0220-F2F3
EXPNO	15
PROCNO	1

F2 - Acquisition Parameters

Date_	20200922
Time	9.15 h
INSTRUM	Spect
PROBHD	Z136122_0002 (
PULPROG	udeft
TD	16384
SOLVENT	DMSO-H6
NS	701
DS	0
SWH	28846.154 Hz
FIDRES	3.521259 Hz
AQ	0.2839893 sec
RG	322
DW	17.333 usec
DE	18.00 usec
TE	298.0 K
D1	3.00000000 sec
D12	0.00002000 sec
D20	200.00000000 sec
TD0	1
SFO1	125.7810526 MHz
NUC1	13C
P1	10.00 usec
P13	2000.00 usec
P26	500.00 usec
PLW1	26.00000000 W
SPNAM[5]	Crp60comp.4
SPOAL5	0.500
SPOFFS5	0 Hz
SPW5	3.97250009 W
SPNAM[8]	Crp60,0.5,20.1
SPOAL8	0.500
SPOFFS8	0 Hz
SPW8	3.97250009 W
SFO2	500.1720007 MHz
NUC2	1H
CPDPRG[2	waltz16
PCPD2	80.00 usec
PLW2	7.00000000 W
PLW12	0.18200999 W

F2 - Processing parameters

SI	32768
SF	125.7681295 MHz
WDW	EM
SSB	0
LB	2.00 Hz
GB	0
PC	2.00

LP0217-F2 / dmso

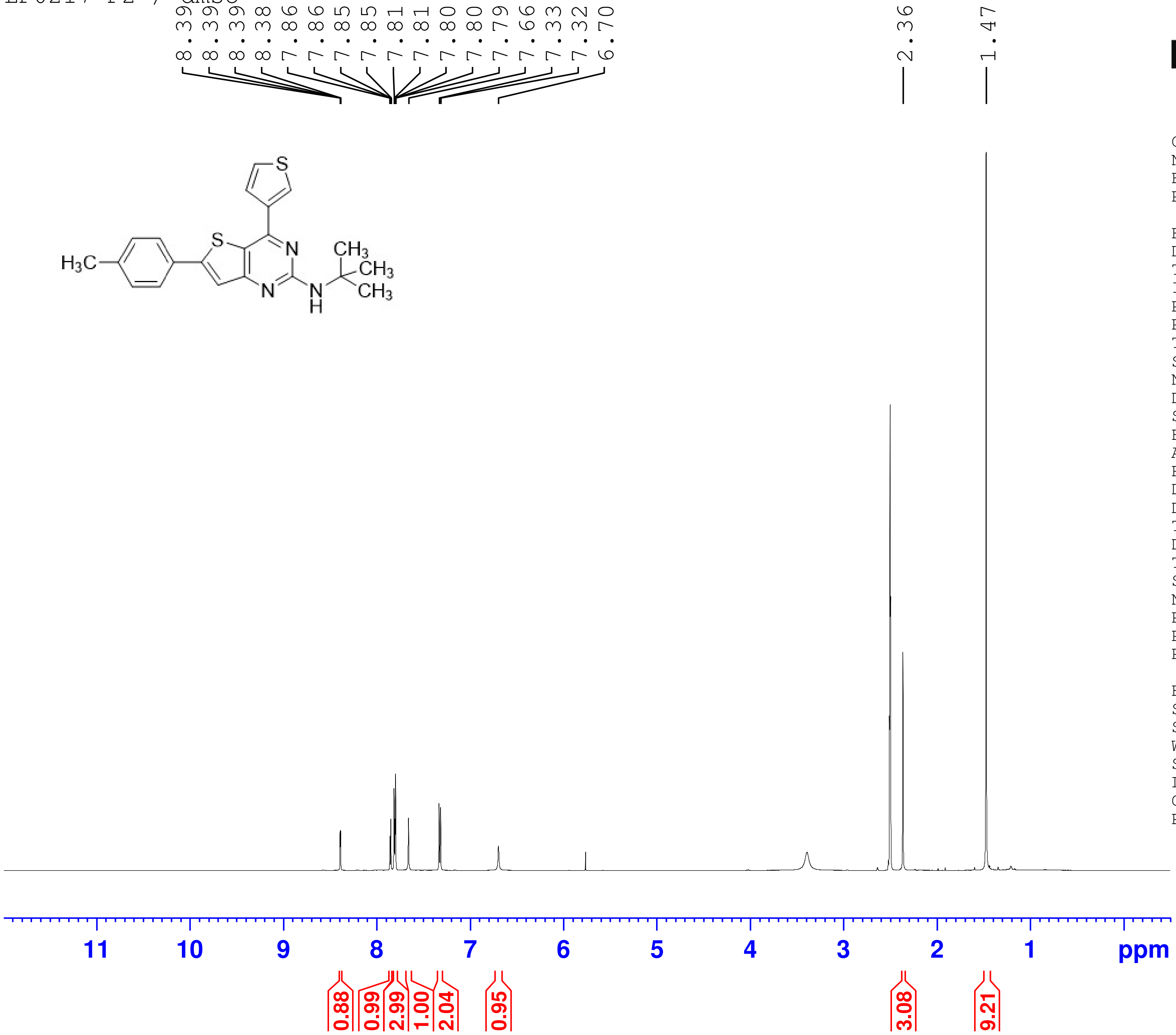

BRUKER

Current Data Parameters

NAME	LAG-7-LP0217-F2
EXPNO	10
PROCNO	1

F2 - Acquisition Parameters

Date_	20200921
Time	11.00 h
INSTRUM	Spect
PROBHD	Z136122_0002 (
PULPROG	zg30
TD	65536
SOLVENT	DMSO
NS	16
DS	2
SWH	10000.000 Hz
FIDRES	0.305176 Hz
AQ	3.2767999 sec
RG	10
DW	50.000 usec
DE	10.00 usec
TE	298.0 K
D1	1.50000000 sec
TD0	1
SFO1	500.1730885 MHz
NUC1	1H
P0	4.30 usec
P1	12.90 usec
PLW1	7.00000000 W

F2 - Processing parameters

SI	65536
SF	500.1700077 MHz
WDW	EM
SSB	0
LB	0.10 Hz
GB	0
PC	2.00

LP0217-F2 / dmso

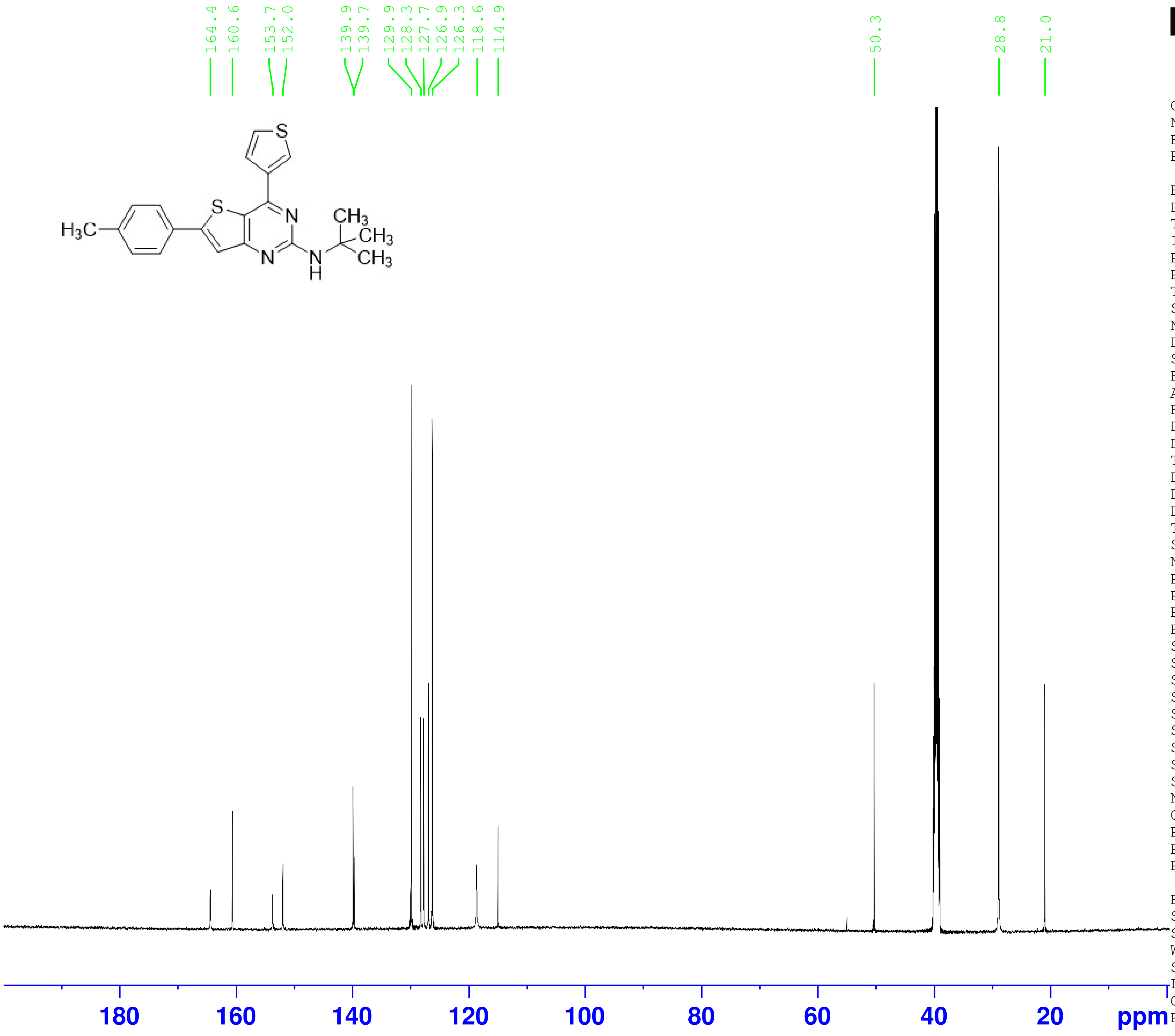

Current Data Parameters
NAME LAG-7-LP0217-F2
EXPNO 12
PROCNO 1

F2 - Acquisition Parameters

Date_	20200921
Time	12.21 h
INSTRUM	Spect
PROBHD	Z136122_0002 (
PULPROG	udeft
TD	16384
SOLVENT	DMSO
NS	637
DS	0
SWH	28846.154 Hz
FIDRES	3.521259 Hz
AQ	0.2839893 sec
RG	322
DW	17.333 usec
DE	18.00 usec
TE	298.0 K
D1	3.00000000 sec
D12	0.00002000 sec
D20	200.00000000 sec
TD0	1
SFO1	125.7810526 MHz
NUC1	13C
P1	10.00 usec
P13	2000.00 usec
P26	500.00 usec
PLW1	26.00000000 W
SPNAM[5]	Crp60comp.4
SPOAL5	0.500
SPOFFS5	0 Hz
SPW5	3.97250009 W
SPNAM[8]	Crp60,0.5,20.1
SPOAL8	0.500
SPOFFS8	0 Hz
SPW8	3.97250009 W
SFO2	500.1720007 MHz
NUC2	1H
CPDPRG[2	waltz16
PCPD2	80.00 usec
PLW2	7.00000000 W
PLW12	0.18200999 W

F2 - Processing parameters

SI	32768
SF	125.7679015 MHz
WDW	EM
SSB	0
LB	2.00 Hz
GB	0
PC	2.00

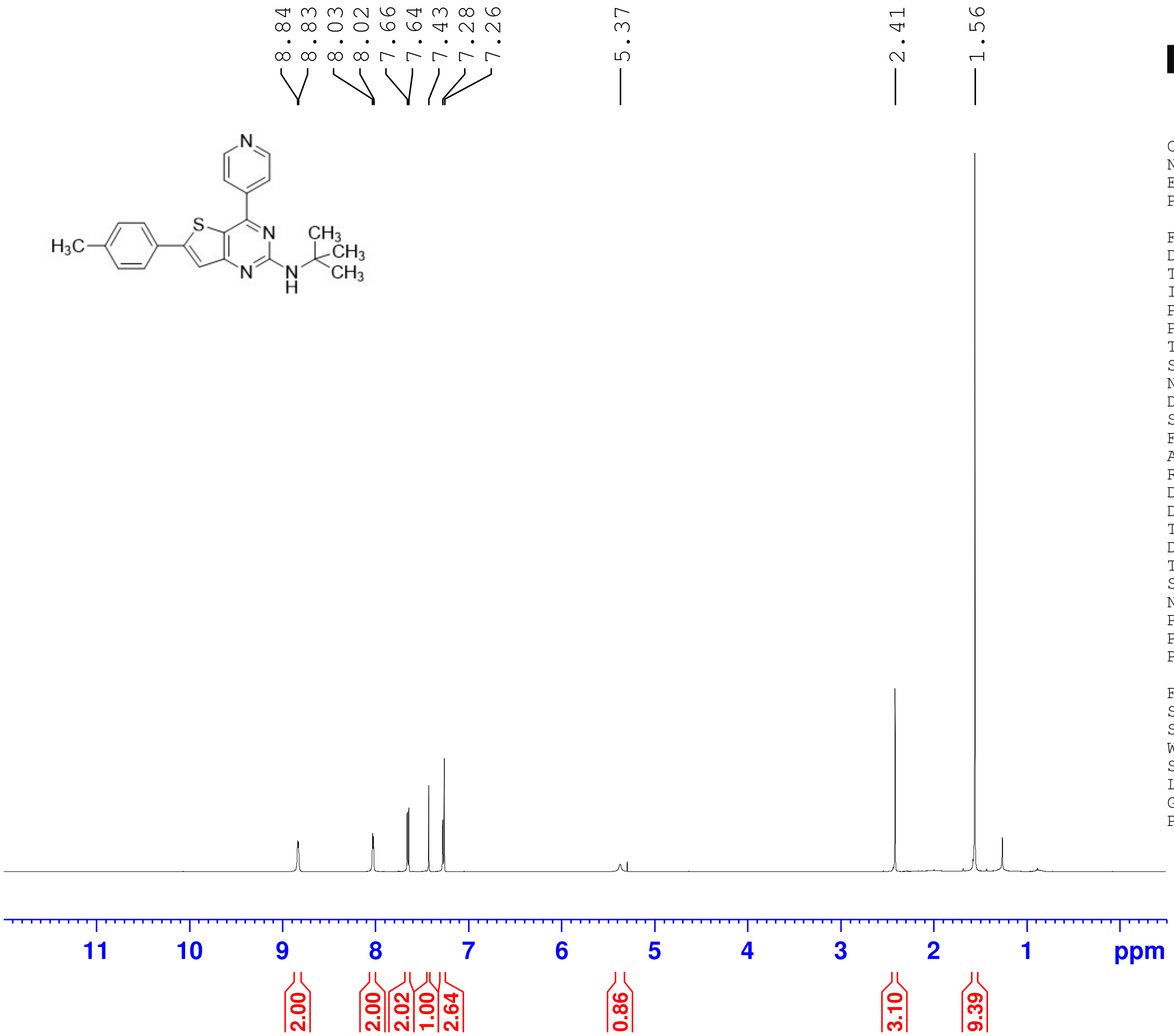

Current Data Parameters

NAME	LAG-169-LP0274
EXPNO	50
PROCNO	1

F2 - Acquisition Parameters

Date_	20220224
Time	17.51 h
INSTRUM	Spect
PROBHD	Z136122_0002 (
PULPROG	zg30
TD	65536
SOLVENT	CDCl3
NS	16
DS	2
SWH	10000.000 Hz
FIDRES	0.305176 Hz
AQ	3.2767999 sec
RG	10
DW	50.000 usec
DE	10.00 usec
TE	298.0 K
D1	1.50000000 sec
TD0	1
SFO1	500.1730885 MHz
NUC1	1H
P0	4.30 usec
P1	12.90 usec
PLW1	7.00000000 W

F2 - Processing parameters

SI	65536
SF	500.1700123 MHz
WDW	EM
SSB	0
LB	0.10 Hz
GB	0
PC	2.00

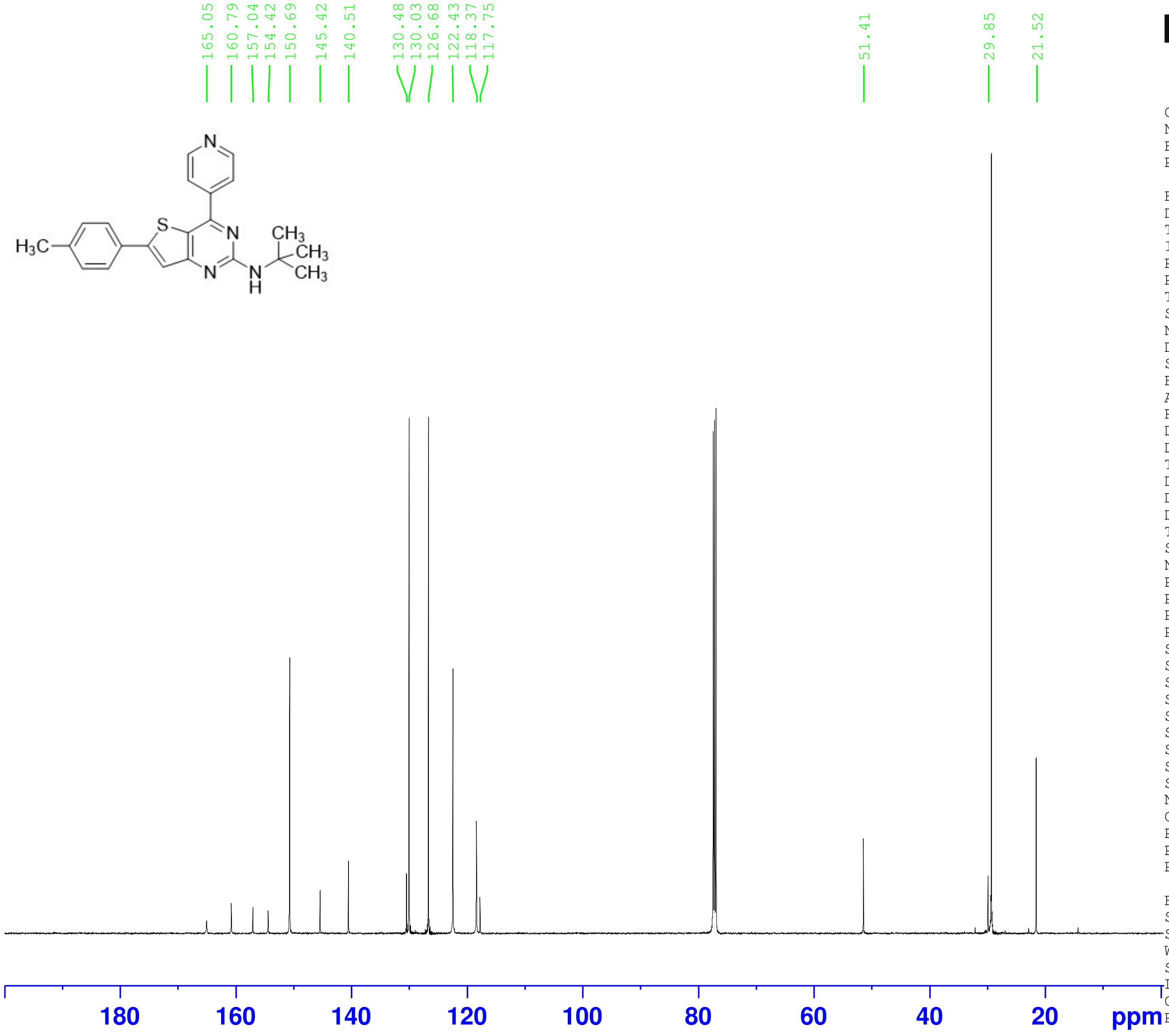

BRUKER

Current Data Parameters
NAME LAG-169-LP0274
EXPNO 53
PROCNO 1

F2 - Acquisition Parameters
Date_ 20220224
Time 21.17 h
INSTRUM Spect
PROBHD Z136122_0002 (
PULPROG udeft
TD 16384
SOLVENT CDCl3
NS 2048
DS 0
SWH 28846.154 Hz
FIDRES 3.521259 Hz
AQ 0.2839893 sec
RG 912
DW 17.333 usec
DE 18.00 usec
TE 298.0 K
D1 3.00000000 sec
D12 0.00002000 sec
D20 200.00000000 sec
TD0 1
SFO1 125.7810526 MHz
NUC1 13C
P1 10.00 usec
P13 2000.00 usec
P26 500.00 usec
PLW1 26.00000000 W
SPNAM[5] Crp60comp.4
SPOAL5 0.500
SPOFFS5 0 Hz
SPW5 3.97250009 W
SPNAM[8] Crp60,0.5,20.1
SPOAL8 0.500
SPOFFS8 0 Hz
SPW8 3.97250009 W
SFO2 500.1720007 MHz
NUC2 1H
CPDPRG[2 waltz16
PCPD2 80.00 usec
PLW2 7.00000000 W
PLW12 0.18200999 W

F2 - Processing parameters
SI 32768
SF 125.7678263 MHz
WDW EM
SSB 0
LB 2.00 Hz
GB 0
PC 2.00

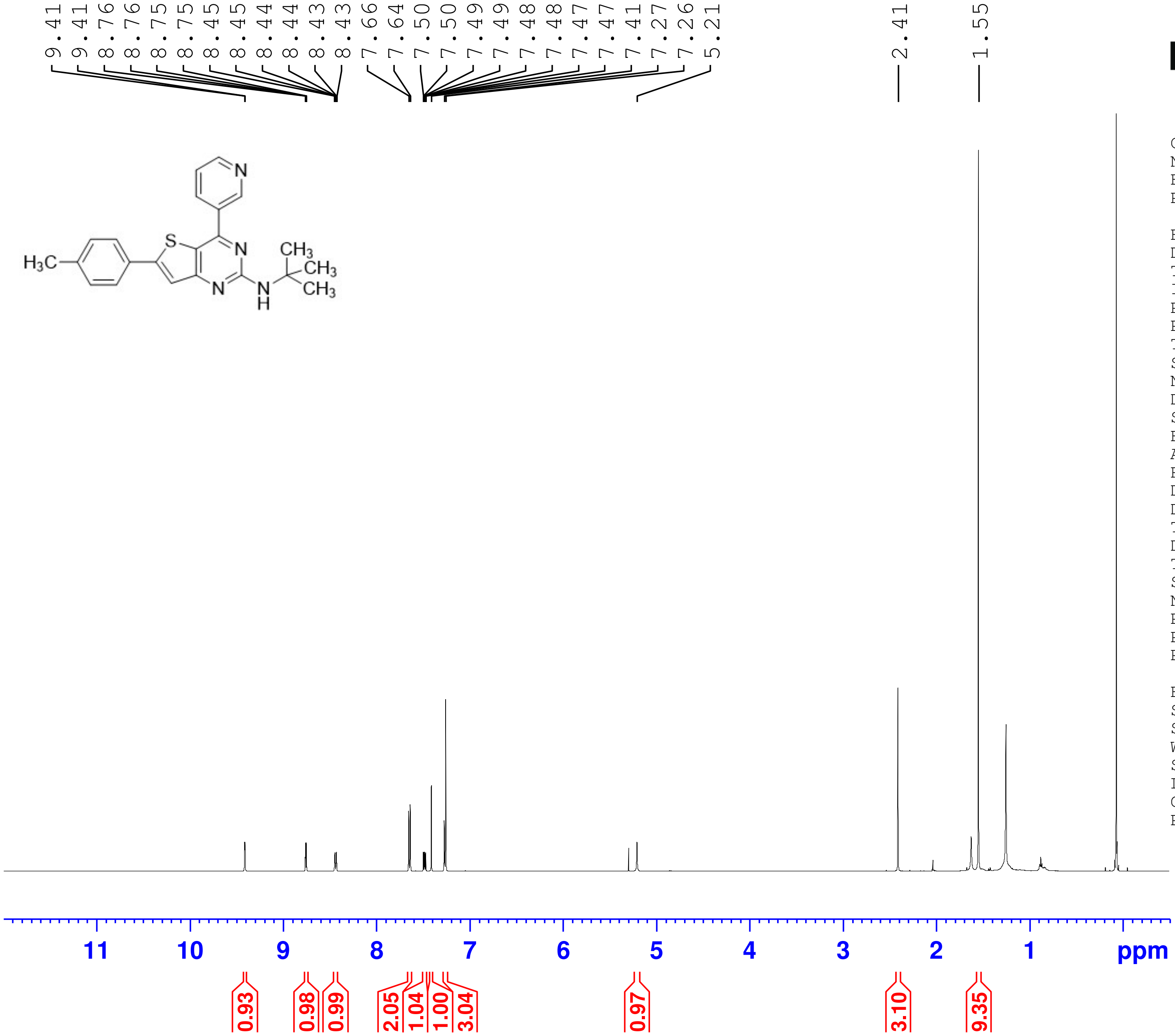

BRUKER

Current Data Parameters
NAME LAG-46-LP0279-F2
EXPNO 10
PROCNO 1

F2 - Acquisition Parameters

Date_	20210123
Time	0.48 h
INSTRUM	Spect
PROBHD	Z136122_0002 (
PULPROG	zg30
TD	65536
SOLVENT	CDCl3
NS	16
DS	2
SWH	10000.000 Hz
FIDRES	0.305176 Hz
AQ	3.2767999 sec
RG	10
DW	50.000 usec
DE	10.00 usec
TE	298.0 K
D1	1.50000000 sec
TD0	1
SFO1	500.1730885 MHz
NUC1	1H
P0	4.30 usec
P1	12.90 usec
PLW1	7.00000000 W

F2 - Processing parameters

SI	65536
SF	500.1700121 MHz
WDW	EM
SSB	0
LB	0.10 Hz
GB	0
PC	2.00

LP0279-F2

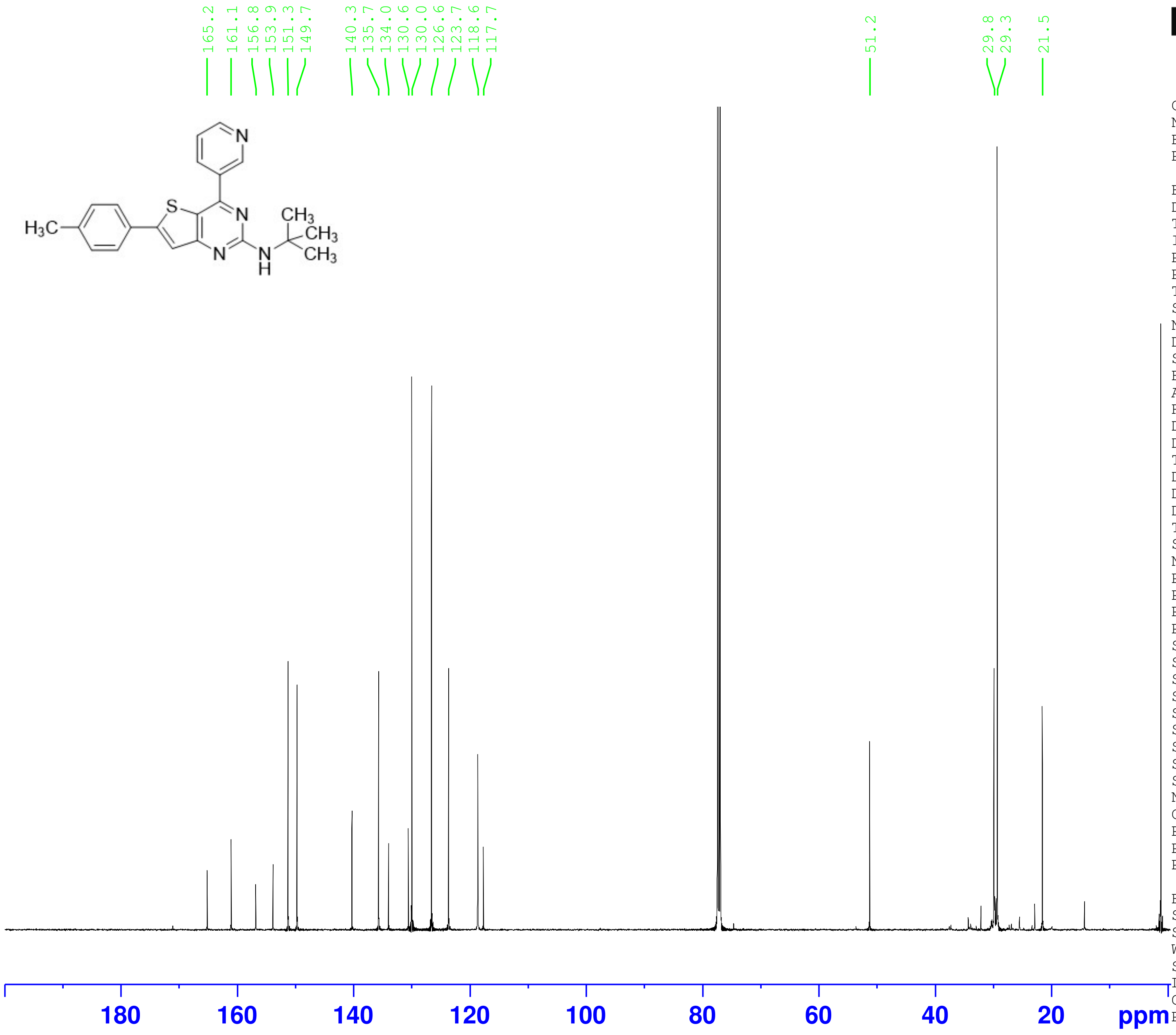

Current Data Parameters
NAME LAG-46-LP0279-F2
EXPNO 14
PROCNO 1

F2 - Acquisition Parameters
Date_ 20210123
Time 5.17 h
INSTRUM Spect
PROBHD Z136122_0002 (
PULPROG udeft
TD 16384
SOLVENT CDCl3
NS 3072
DS 0
SWH 28846.154 Hz
FIDRES 3.521259 Hz
AQ 0.2839893 sec
RG 912
DW 17.333 usec
DE 18.00 usec
TE 298.0 K
D1 3.00000000 sec
D12 0.00002000 sec
D20 200.00000000 sec
TD0 1
SFO1 125.7810526 MHz
NUC1 13C
P1 10.00 usec
P13 2000.00 usec
P26 500.00 usec
PLW1 26.00000000 W
SPNAM[5] Crp60comp.4
SPOAL5 0.500
SPOFFS5 0 Hz
SPW5 3.97250009 W
SPNAM[8] Crp60,0.5,20.1
SPOAL8 0.500
SPOFFS8 0 Hz
SPW8 3.97250009 W
SFO2 500.1720007 MHz
NUC2 1H
CPDPRG[2 waltz16
PCPD2 80.00 usec
PLW2 7.00000000 W
PLW12 0.18200999 W

F2 - Processing parameters
SI 32768
SF 125.7678297 MHz
WDW EM
SSB 0
LB 2.00 Hz
GB 0
PC 2.00

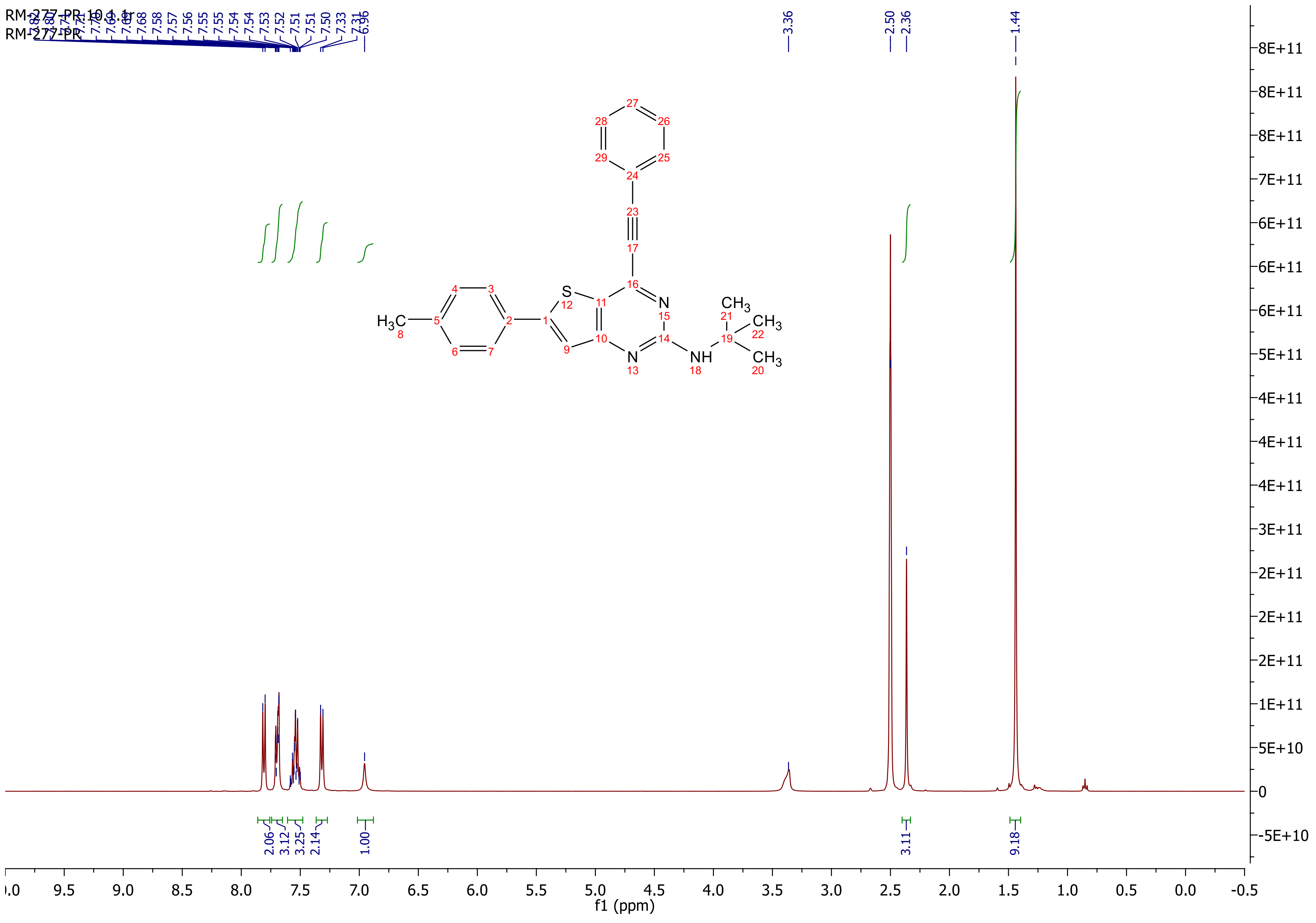

RM-277-PR.10.1.1r
RM-277-PR
7.50
7.33
7.31
6.96
3.36
2.50
2.36
1.44
2.06
3.12
3.25
2.14
1.00
3.11
9.18
f1 (ppm)

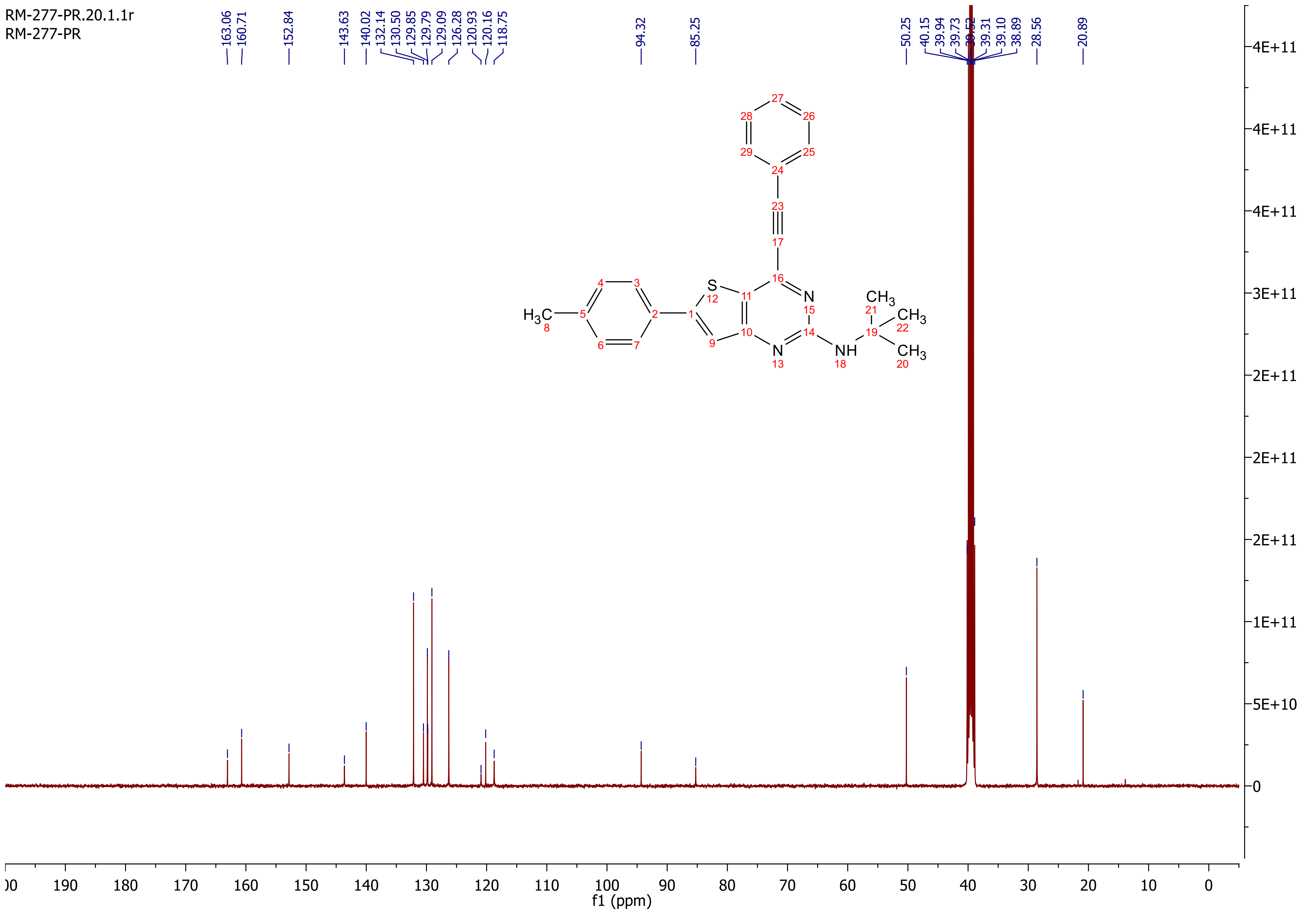

RM-277-PR.20.1.1r
RM-277-PR
163.06
160.71
152.84
143.63
140.02
132.14
130.50
129.85
129.79
129.09
126.28
120.93
120.16
118.75
94.32
85.25
50.25
40.15
39.94
39.73
39.52
39.31
39.10
38.89
28.56
20.89
S
N
NH
H_3C
CH_3
f1 (ppm)

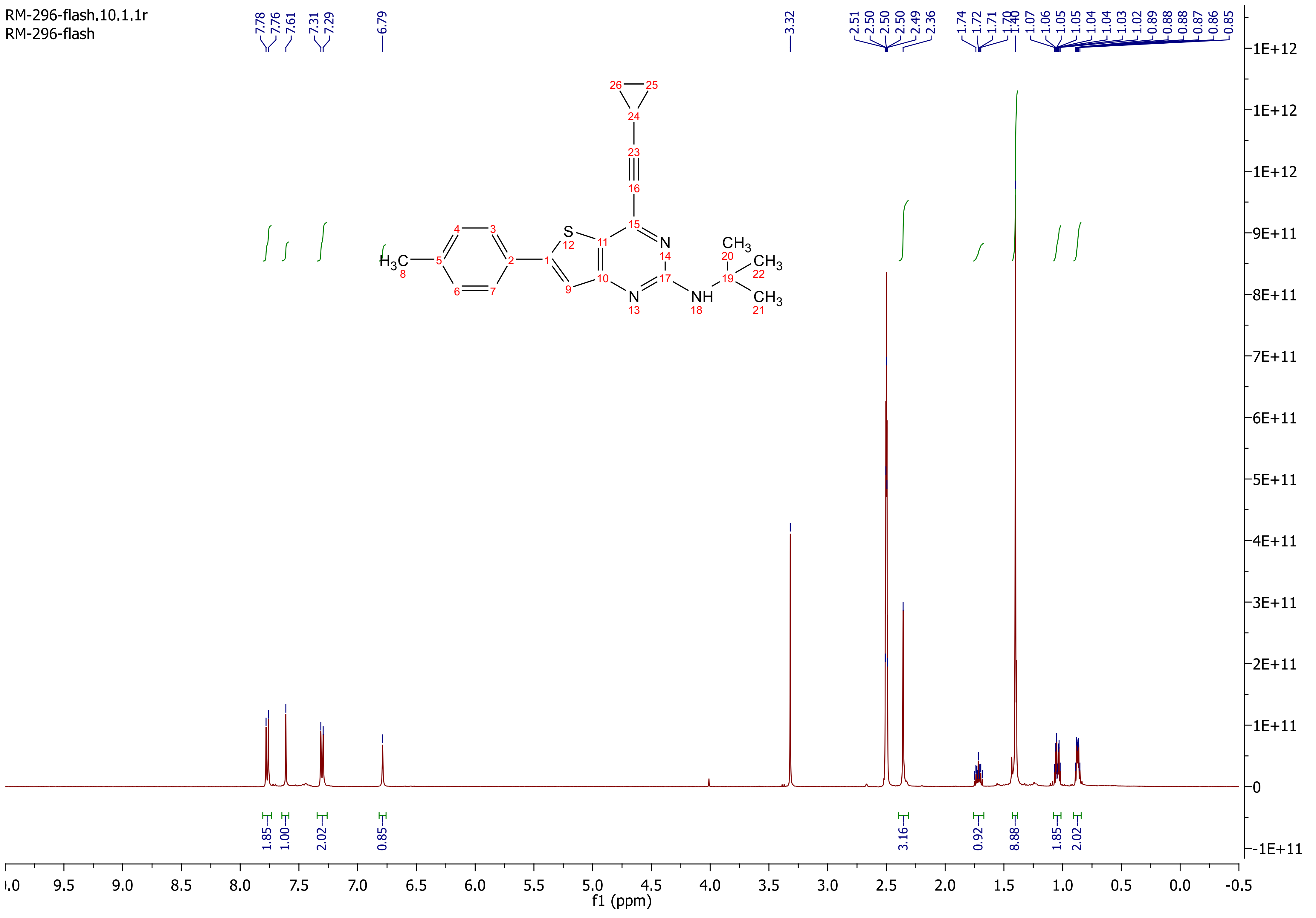
RM-296-flash.10.1.1r
RM-296-flash
7.78
7.76
7.61
7.31
7.29
6.79
3.32
2.51
2.50
2.50
2.50
2.49
2.36
1.74
1.72
1.71
1.70
1.40
1.07
1.06
1.05
1.05
1.04
1.04
1.03
1.02
0.89
0.88
0.88
0.87
0.86
0.85
1.85
1.00
2.02
0.85
3.16
0.92
8.88
1.85
2.02
f1 (ppm)

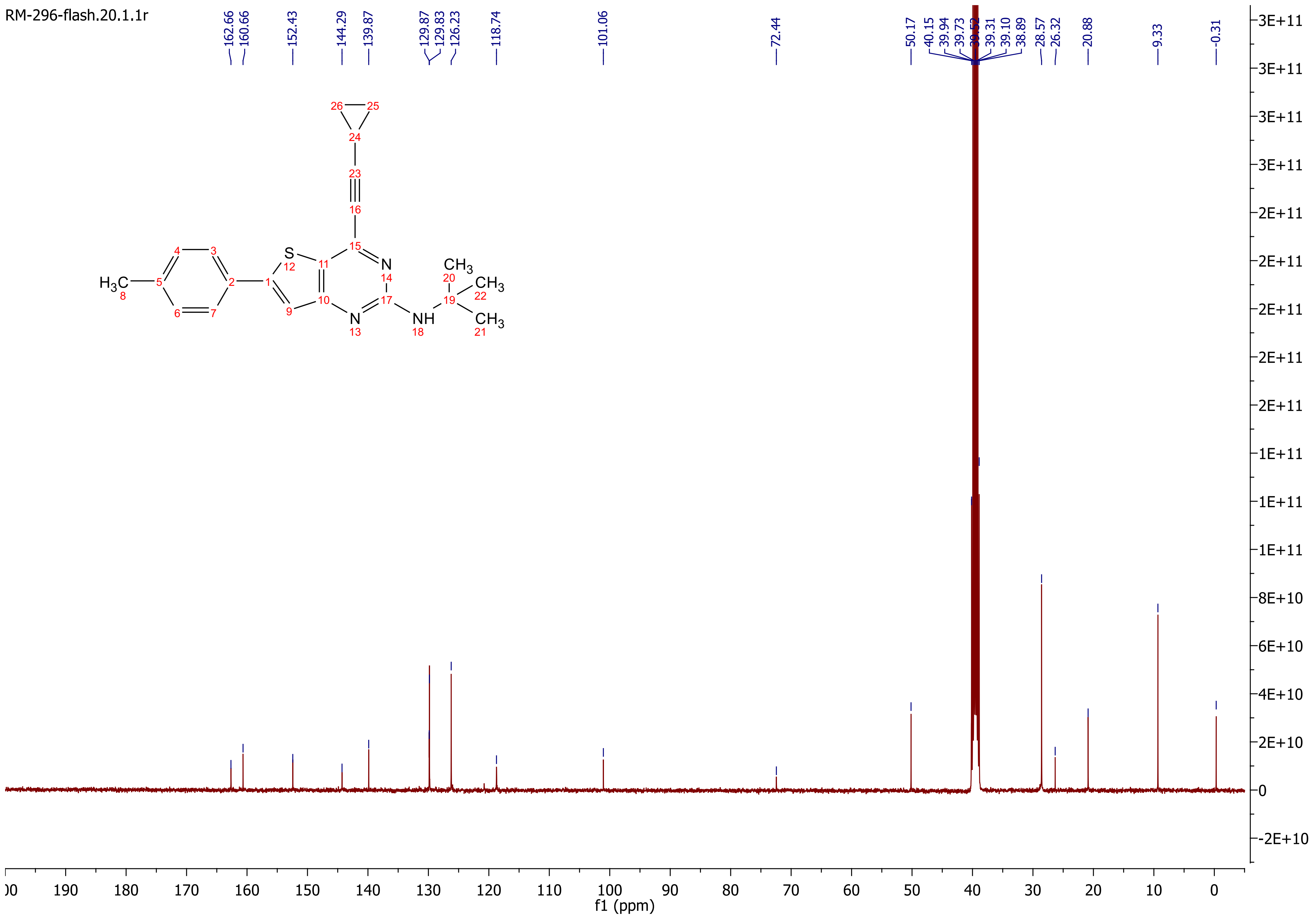

RM-296-flash.20.1.1r
162.66
160.66
152.43
144.29
139.87
129.87
129.83
126.23
118.74
101.06
72.44
50.17
40.15
39.94
39.73
39.52
39.31
39.10
38.89
28.57
26.32
20.88
9.33
-0.31
f1 (ppm)

RM-287-PR.10.1.1r

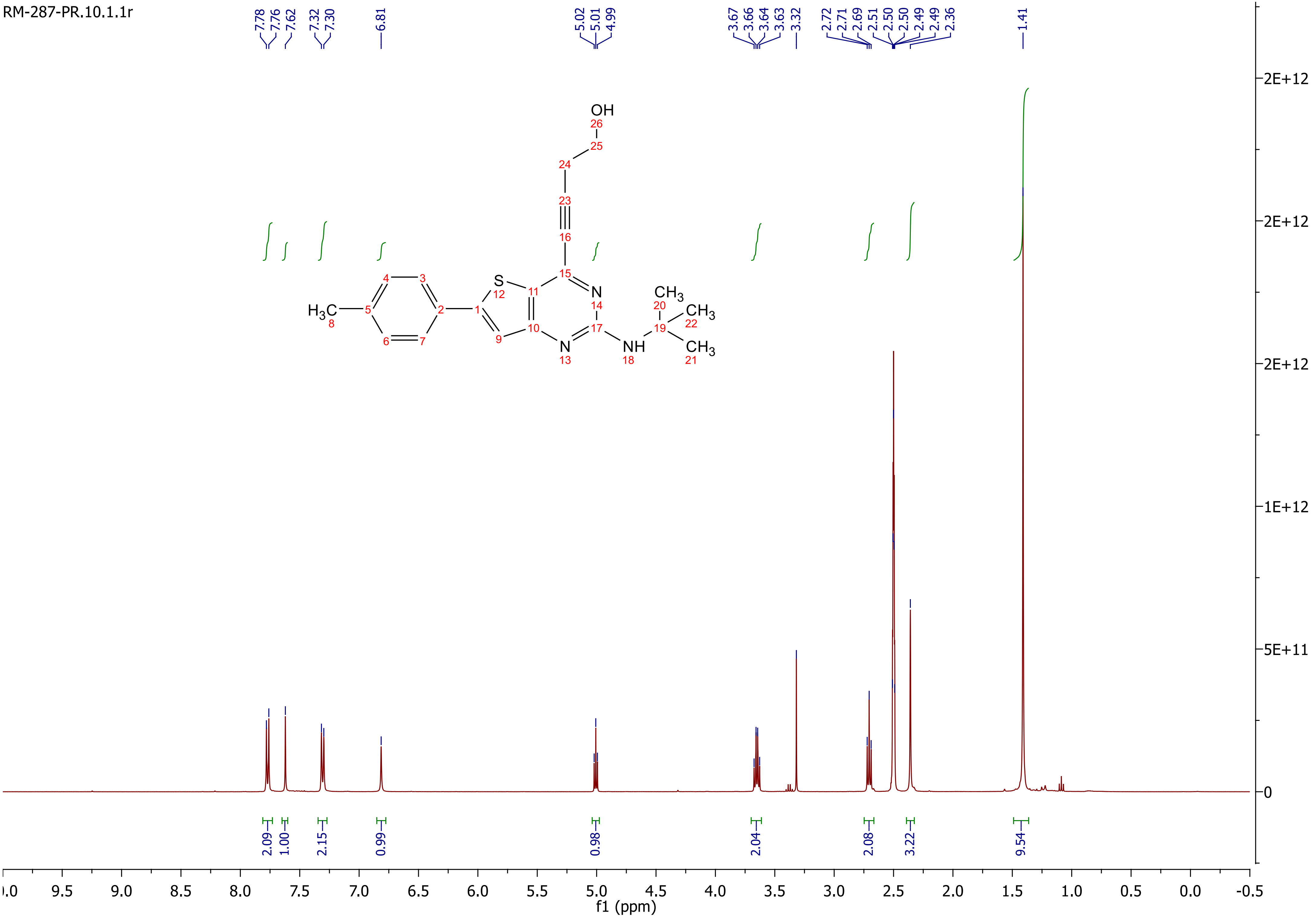

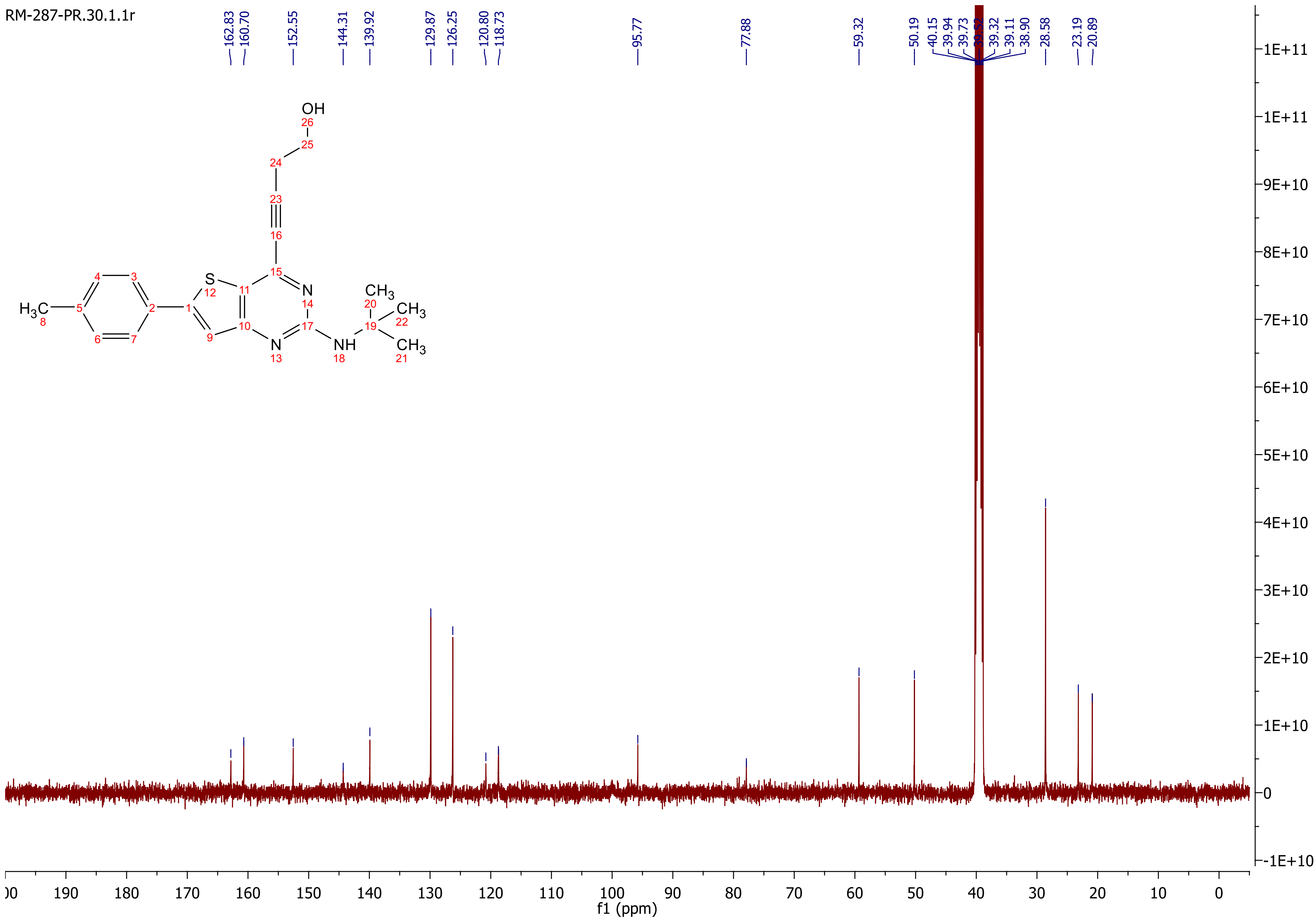
RM-287-PR.30.1.1r
162.83
160.70
152.55
144.31
139.92
129.87
126.25
120.80
118.73
95.77
77.88
59.32
50.19
40.15
39.94
39.73
39.52
39.32
39.11
38.90
28.58
23.19
20.89
OH
S
N
NH
H3C
CH3
1E+11
9E+10
8E+10
7E+10
6E+10
5E+10
4E+10
3E+10
2E+10
1E+10
0
-1E+10
f1 (ppm)

LP0303-F3

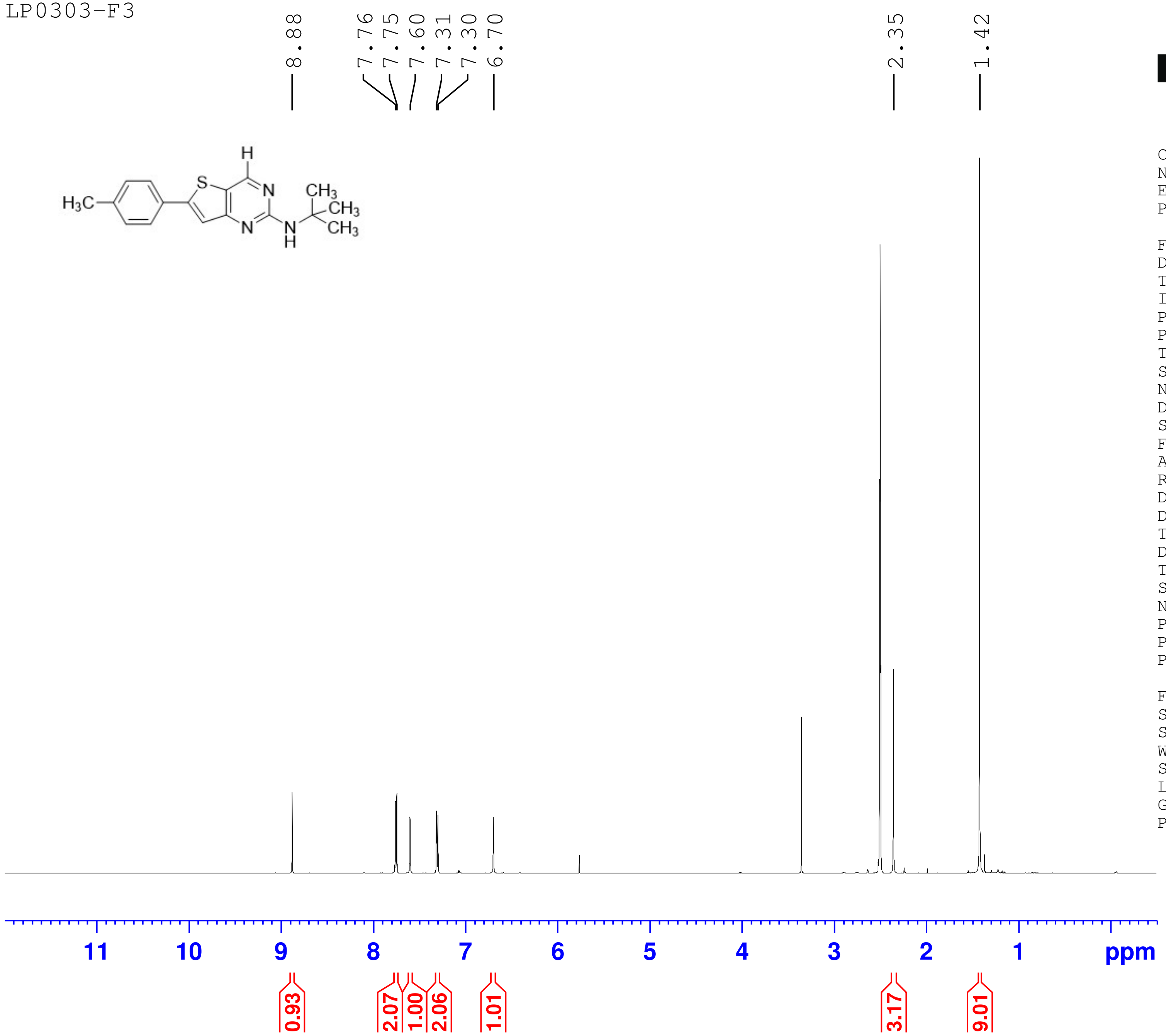

BRUKER

Current Data Parameters
NAME LAG-67-LP0303-F3
EXPNO 10
PROCNO 1

F2 - Acquisition Parameters
Date_ 20210320
Time 15.17 h
INSTRUM Spect
PROBHD Z136122_0002 (
PULPROG zg30
TD 65536
SOLVENT DMSO
NS 16
DS 2
SWH 10000.000 Hz
FIDRES 0.305176 Hz
AQ 3.2767999 sec
RG 10
DW 50.000 usec
DE 10.00 usec
TE 298.0 K
D1 1.50000000 sec
TD0 1
SFO1 500.1730885 MHz
NUC1 1H
P0 4.30 usec
P1 12.90 usec
PLW1 7.00000000 W

F2 - Processing parameters
SI 65536
SF 500.1700107 MHz
WDW EM
SSB 0
LB 0.10 Hz
GB 0
PC 2.00

LP0303-F3

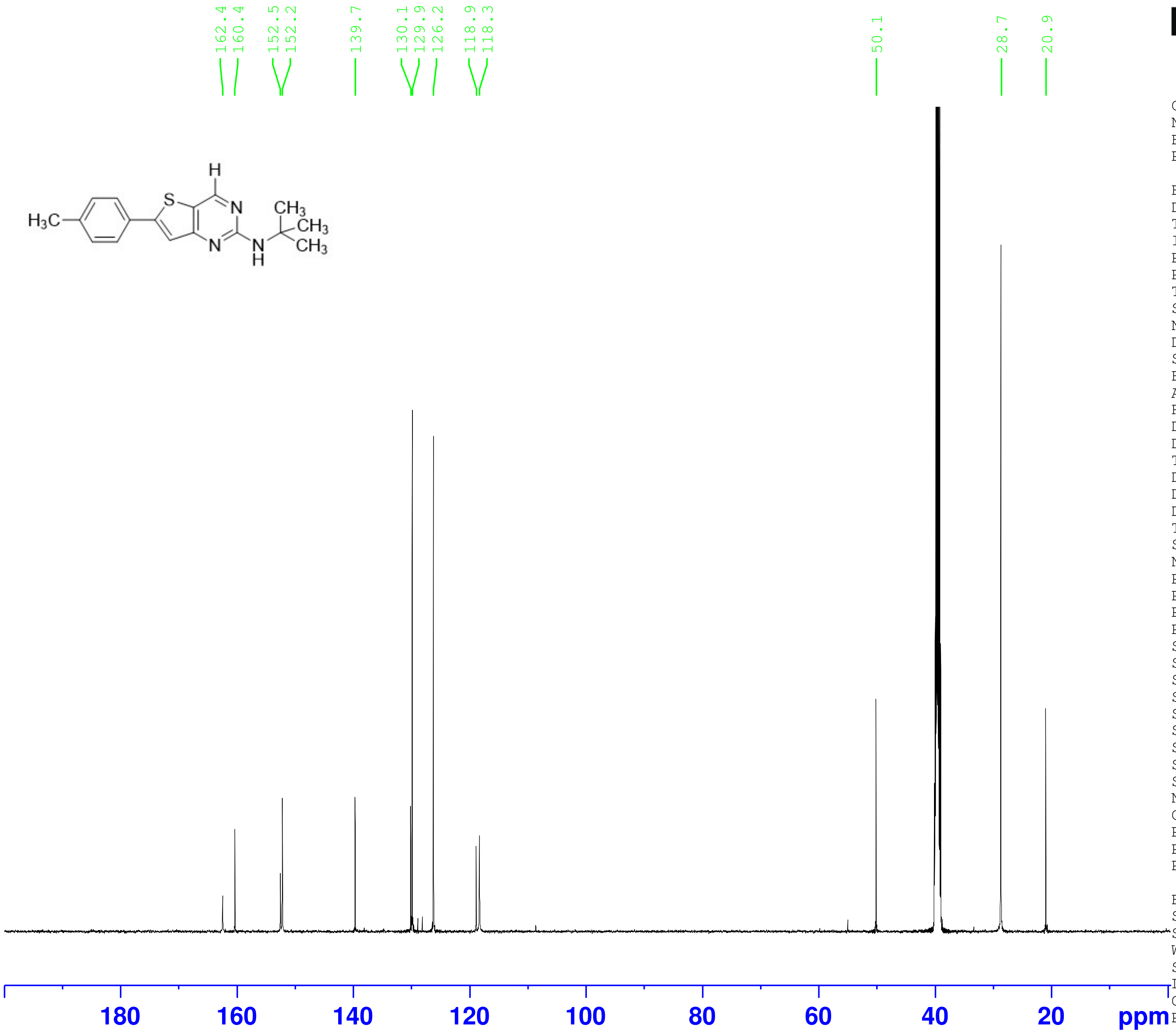

Current Data Parameters

NAME	LAG-67
EXPNO	11
PROCNO	1

F2 - Acquisition Parameters

Date_	20210320
Time	16.30 h
INSTRUM	Spect
PROBHD	Z136122_0002 (
PULPROG	udeft
TD	16384
SOLVENT	DMSO
NS	1024
DS	0
SWH	28846.154 Hz
FIDRES	3.521259 Hz
AQ	0.2839893 sec
RG	912
DW	17.333 usec
DE	18.00 usec
TE	298.0 K
D1	3.00000000 sec
D12	0.00002000 sec
D20	200.00000000 sec
TD0	1
SFO1	125.7810526 MHz
NUC1	13C
P1	10.00 usec
P13	2000.00 usec
P26	500.00 usec
PLW1	26.00000000 W
SPNAM[5]	Crp60comp.4
SPOAL5	0.500
SPOFFS5	0 Hz
SPW5	3.97250009 W
SPNAM[8]	Crp60,0.5,20.1
SPOAL8	0.500
SPOFFS8	0 Hz
SPW8	3.97250009 W
SFO2	500.1720007 MHz
NUC2	1H
CPDPRG[2	waltz16
PCPD2	80.00 usec
PLW2	7.00000000 W
PLW12	0.18200999 W

F2 - Processing parameters

SI	32768
SF	125.7679036 MHz
WDW	EM
SSB	0
LB	2.00 Hz
GB	0
PC	2.00
